# Open randomised trial of the (Arabin) pessary to prevent preterm birth in twin pregnancy with health economics and acceptability: STOPPIT-2—a study protocol

**DOI:** 10.1136/bmjopen-2018-026430

**Published:** 2018-12-06

**Authors:** Jane E Norman, John Norrie, Graeme Maclennan, David Cooper, Sonia Whyte, Sarah Cunningham Burley, Joel B E Smith, Andrew Shennan, Stephen C Robson, Steven Thornton, Mark D Kilby, Neil Marlow, Sarah J Stock, Philip R Bennett, Jane Denton

**Affiliations:** 1 Tommy’s Centre for Maternal and Fetal Health, MRC Centre for Maternal and Fetal Health, University of Edinburgh, Edinburgh, UK; 2 Edinburgh Clinical Trials Unit, University of Edinburgh, Edinburgh, UK; 3 Centre for Healthcare Randomised Trials, University of Aberdeen, Aberdeen, UK; 4 Usher Institute, The University of Edinburgh, Edinburgh, UK; 5 Health Economics Research Centre (HERC), Nuffield Department of Population Health, University of Oxford, Oxford, UK; 6 Tommy’s Centre for Maternal and Fetal Health, King’s College London, London, UK; 7 Institute of Cellular Medicine, University of Newcastle, Newcastle, UK; 8 Barts and the London School of Medicine and Dentistry, Queen Mary University of London, London, UK; 9 Fetal Medicine Centre, Birmingham Women’s and Children’s Foundation Trust and College of Medical and Dental Science, University of Birmingham, Birmingham, UK; 10 Institute for Women’s Health, University College London, London, UK; 11 Department of Surgery and Cancer, Imperial College London, London, UK; 12 Multiple Births Foundation, London, UK

**Keywords:** preterm birth, cervical pessary, twin pregnancy, preterm labour

## Abstract

**Introduction:**

The STOPPIT-2 study aims to determine the clinical utility of the Arabin cervical pessary in preventing preterm birth in women with a twin pregnancy and a short cervix, about which there is current uncertainty. STOPPIT-2 will resolve uncertainty around effectiveness for women with a twin pregnancy and a cervical length of 35 mm or less, define adverse effects, ascertain acceptability and estimate National Health Service costs and savings.

**Methods:**

STOPPIT-2 is a pragmatic multicentre open-label randomised controlled trial. Consenting women with twin pregnancy will have an transvaginal ultrasound scan of their cervical length performed between 18+0 and 20+6 weeks’ gestation by an accredited practitioner: women with a cervical length of ≤35 mm will be eligible for inclusion in the treatment phase of the study. The intervention by the insertion of the Arabin cervical pessary will be compared with standard treatment (no pessary).

The primary outcomes are (obstetric) spontaneous onset of labour for the mother leading to delivery before 34 weeks’ gestation and (neonatal) a composite of specific adverse outcomes or death occurring up to the end of the first 4 weeks after the estimated date of delivery to either or both babies.

We plan to recruit 500 women in the treatment phase of the study. Assuming a treatment effect of 0.6, and background rates of 35% and 18%, respectively, for each of the primary outcomes, our study has 85% power to detect a difference between the intervention and the control groups.

**Analysis:**

Data will be analysed on the intention-to-treat principle.

**Ethics:**

STOPPIT-2 was approved by the South East Scotland Ethics Committee 02 on 29 August 2014, reference number 14/SS/1031 IRAS ID 159610.

**Dissemination:**

Peer reviewed journals, presentations at national and international scientific meetings.

**Trial registration number:**

ISRCTN98835694 and NCT02235181.

**Strengths and limitations of this study**

- Randomised multicentre trial.
- Cervical length measured by accredited individuals.
- Prespecified cervical length threshold for randomisation.
- Larger sample size than previously studied.
- Inability to blind women or caregivers to treatment allocation.

## Introduction

Preterm birth is common in twin pregnancy. The rate of twinning itself is increasing. Twinning is associated with significant death or serious morbidity for the babies, but there are no effective treatments. In 2010, 52.7% of multiple births were preterm, and there is a clear health need for prevention of preterm birth in twins. This excess of prematurity (compared with singletons) leads to neonatal and infant mortality rates of 14.2 and 18.8 per 1000 live births, respectively (over fivefold that of singletons). Progesterone appears ineffective in preventing preterm birth in twins,^1^ and cerclage appears harmful.^2^ Prematurity is thought to account for over 70% of twin neonatal deaths and adversely affects fetal survivors, with increased risks of future respiratory problems, motor and sensory impairment, learning difficulties and social and behavioural difficulties.

The costs to the National Health Service (NHS) of prematurity in twins are considerable. Twins alone account for over 20% of neonatal unit cot stays, a significant excess given they comprise only 2% of all births. Together, the complications of preterm birth result in an estimated annual cost of £2.946 billion to the public purse in England and Wales (2006 prices).^3^ Twins make a major contribution to this. There is a clear expressed need for innovative interventions to reduce preterm birth in both high-income and low-income countries.^4 5^ The 2011 National Institute for Health and Care Excellence (NICE) Multiple Pregnancy Guideline Group noted that bed rest at home or in hospital, progesterone, cervical cerclage and oral tocolytics are all ineffective at preventing preterm birth in twins, concluding that alternative effective interventions are urgently required. The guideline predated the publication of studies on pessary to prevent preterm birth in twins, and there was no attempt to evaluate effectiveness. The Cochrane review on ‘cervical pessary for preventing preterm birth’^6^ identified only one randomised trial (in singletons) that ‘showed the beneficial effect of the cervical pessary in preventing preterm birth’ and called for randomised trials to be performed in multiple pregnancy.^6^

**Figure 1:**
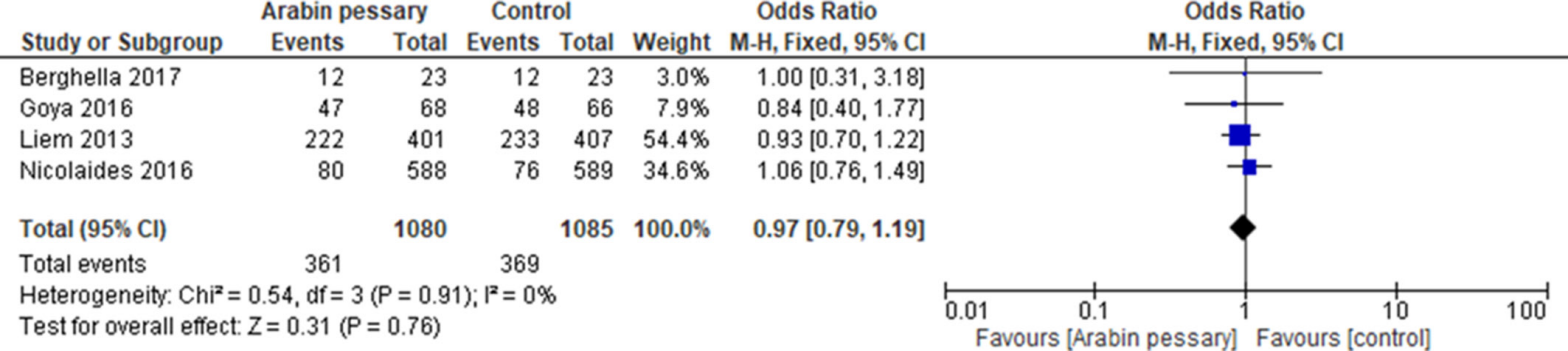
Forest plot of randomised trials to determine the efficacy of a cervical pessary to prevent preterm birth (as defined in each individual study) in twin pregnancy.

Four randomised trials have reported efficacy of cervical pessaries to prevent preterm birth in twins. A summary forest plot on the efficacy of the pessary to prevent preterm birth (using the entire population randomised each study) is shown in the Figure 1. The first trial to be published was the ProTwin study, which randomised 813 women with twin pregnancy to treatment with an Arabin pessary (inserted between 16 and 20 weeks’ gestation) or to standard treatment.^7^ Although the pessary did not reduce preterm birth or the primary neonatal composite outcome overall, both of these events were significantly reduced in the prespecified subgroup of women with a cervical length less than the 25th centile (n=143) with a relative risk (RR) (95% CI) of preterm birth before 32 weeks of 0.49 (95% CI 0.24 to 0.97) and RR (95% CI) of composite neonatal outcome of 0.42 (95% CI 0.19 to 0.91). A subsequent randomised study of 1180 unselected women showed no difference in the rate of the primary outcome of spontaneous birth <34 weeks (13.6% vs 12.9%; relative risk 1.054, 95% CI 0.787 to 1.413; p=0.722).^8^ A preplanned subgroup analysis of 214 women with a cervical length of ≤25 mm also showed no benefit of the pessary (HR 1.256; 95% CI 0.760 to 2.074; p=0.374). These data contrast with a smaller study published the same year, showing that, in women with a cervical length of ≤25 mm, spontaneous preterm birth was less common in the pessary group 11/68 (16.2%) compared with the expectant management group (26/66 (39.4%)).^9^ Lastly, the Prevention of preterm birth with pessary in twins (PoPPT) study of women with twin pregnancy and a cervical length ≤30 mm at 18+0 to 27+6 weeks' gestation randomised 46 women and found no difference in the primary outcome of preterm birth <34 weeks (39% vs 35%; relative risk, 1.13 (95% CI 0.53 to 2.40).^10^

Taken together, these data suggest that the cervical pessary does not work in unselected women but may work in women with a short cervix (at higher risk of preterm birth); consequently, STOPPIT-2 will test the efficacy of the Arabin pessary in women with a short cervix. A cervical length threshold for recruitment of less than or equal to the 30th centile was chosen to maximise the number of women who could benefit. When planning the trial, it was thought that the 30th centile for cervical length would equate to a cervix of ≤30 mm in length, based on a large UK study.^11^ However, a meta-analysis published in the early phases of the pilot for STOPPIT-2,^12^ and data from the first 20 women in STOPPIT-2 itself, suggested that the 30th centile of cervical length in twins at 18–21 weeks’ gestation is 35 mm. A cervical length threshold of ≤35 mm (which we assume to be the 30th centile) was therefore used for eligibility. The quality control measures of the Fetal Medicine Foundation (http://www.fetalmedicine.com), or the Perinatal Quality Foundation (https://clear.perinatalquality.org), are employed to ensure validity of cervical length measurements.

Research priorities identified by NICE include randomised trials to determine ‘[effective interventions] in preventing spontaneous preterm birth in women with twin[s], especially in those at high risk of preterm birth’. Additionally, the Preterm Birth Clinical Study Group of the Royal College of Obstetricians and Gynaecologists (RCOG) identified ‘Efficacy of the Arabin pessary’ among its top five research priorities. We are aware of several other studies in active or planned recruitment comparing a cervical pessary with standard care for prevention of preterm birth or its consequences in women with a preterm birth including NCT03418311 (planned n=672), NCT03058536 (planned n=312), ACTRN12616000875404 (planned n=140), NCT02708264 (planned n=242), NCT02350231 (planned n=100) and NCT01334489 (planned n=352).

After publication of STOPPIT-2 results, we plan to collaborate in subsequent individual patient data meta-analyses and update the economic decision model in light of new evidence. If the cervical pessary is effective in reducing preterm birth in twin pregnancy, it could make a major contribution to human health and ensure a more efficient allocation of resources in the UK NHS. However, we need to determine whether it is effective (and safe), since the evidence to date is not strong or robust enough to support its adoption as a first-line intervention.

## Methods

### Design

STOPPIT-2 is a multicentre open-label randomised controlled trial of the Arabin pessary (CE marked device) versus standard treatment in women with twin pregnancy recruited from NHS antenatal clinics. The study is in two phases: a screening phase, in which women with a short cervix (cervical length of ≤35 mm) are identified, and a treatment phase, in which women with a short cervix will be randomised to either treatment with Arabin pessary or standard treatment. An internal pilot will be performed to assess recruitment rates and to determine feasibility.

### Objectives

#### Primary objective

The primary objective of this study is to test the hypothesis that the Arabin cervical pessary reduces spontaneous preterm birth in women with a twin pregnancy and a short cervix (≤35 mm), reducing adverse neonatal outcomes and healthcare costs. We will also address the hypothesis that the pessary is acceptable to pregnant women. Subgroup analyses will determine effectiveness in women with a cervical length ≤25 mm and in women with a dichorionic pregnancy.

#### Secondary objectives

The secondary objectives are to confirm (using the entire cohort of women undergoing cervical length scanning) the profile of cervical length measurements in women with twin pregnancy in the UK and the positive and negative likelihood ratios of a variety of cervical length thresholds to determine spontaneous preterm birth before 34 weeks’ gestation.

### Randomisation

Randomisation to Arabin pessary or standard treatment is performed by the central randomisation facility at the study data centre using a web portal. The allocation sequence will be generated by computer. Participants will be assigned a unique study identifier, and staff will be required to enter minimal patient details prior to randomisation. Randomisation will be minimised with a random element rather than stratified given the properties of minimisation in ensuring comparability between the groups. Minimisation variables include booking hospital, cervical length and chorionicity (mono or dichorionic) but are not limited to these. The study is open (ie, not masked), so that both the participant and the investigator will know which treatment has been allocated. The investigator will inform the woman of treatment allocation after randomisation.

### Population

We will recruit pregnant women who meet the study eligibility criteria and who are booked for delivery at one of the participating sites. Case notes will be reviewed by the local clinical or research teams prior to recruitment. We anticipate that all eligible women expecting twins and who are attending for antenatal care in each of the sites will be invited to participate. Eligible women will normally be informed of the study at routine antenatal appointments. The following inclusion and exclusion criteria will apply.

Inclusion criteria (all must apply):

- Presenting with twin pregnancy (monochorionic or dichorionic).
- Gestation established by scan at ≤16 weeks according to NICE guidelines.
- Aged 16 years or older.
- Wishing to participate in both the screening and randomisation phase of the study.
- Known chorionicity (as defined by first trimester ultrasound screening).

Exclusion criteria – screening phase (none must apply):

- Unable to give written informed consent.
- Known significant congenital structural or chromosomal fetal anomaly at the time of inclusion.
- Existing or planned cervical cerclage in the current pregnancy.
- Existing or planned (prior to 20+6 weeks’ gestation) treatment for twin to twin transfusion syndrome in the current pregnancy.
- Suspected or proven rupture of the fetal membranes at the time of recruitment.
- Bulging fetal membranes at the time of recruitment.
- Singleton pregnancy or higher order multiple pregnancies and/or women who have had any fetal death (ie, fetal heart and previously detected) in the index pregnancy (prior to randomisation).
- Known sensitivity, contraindication or intolerance to silicone.
- Involved in a clinical trial of an investigational medicinal product, phase 1 study or investigating a treatment for the prevention of preterm birth.
- Monochorionic monoamniotic pregnancy.
- Heavy bleeding due to a low-lying placenta prior to randomisation.

Inclusion criteria – screening phase:

- Cervical length ≤35 mm at 18+0–20+6 weeks’ gestation confirmed by an accredited clinician.

Exclusion criteria – treatment phase (none must apply):

- Cervical length >35 mm at 18+0–20+6 weeks’ gestation.
- Cervical length not measured at 18+0–20+6 weeks’ gestation.
- Bulging fetal membranes at the time of pessary insertion.
- Suspected or proven rupture of the fetal membranes at the time of pessary insertion.

To assess eligibility for the treatment phase, all women who fulfil the inclusion criteria for the screening phase will undergo ultrasound measurement of cervical length between 18+0 and 20+6 weeks’ gestation by an accredited operator (see later). If convenient, cervical length measurement may be done at the time of the routine fetal anomaly scan. In centres where cervical length measurements are part of routine clinical care women may be consented after the cervical length and will not be asked to have a further TV scan, provided:

- The transvaginal scan measurement was taken by an accredited operator (completed cervical length education and review (CLEAR, USA) or Fetal Medicine Foundation cervical length training) and is delegated by the principal investigator (PI).
- The TV scan was conducted between 18+0 and 20+6 weeks’ gestation.
- The woman is eligible (as per the above inclusion/exclusion criteria) and the pregnancy is not ≥21 weeks’ gestation.

Images of the cervical length measurements will be anonymised and uploaded to the electronic case report form (eCRF).

Women with a cervical length of ≤35 mm will continue to the treatment phase, following verbal reconfirmation of intent to participate and randomised to either the intervention or the comparator group as described below.

### Enrolment and adherence of participating centres

We will recruit from UK NHS and European hospitals. Sufficient centres will be involved to ensure that our planned sample size is achieved. Regular meetings will be held with clinicians and research staff from recruiting centres to maintain study enthusiasm and to achieve our planned sample size.

### Intervention

The intervention is the Arabin pessary, inserted through the vagina and around the cervix between 18+0 and 20+6 weeks’ gestation and given in addition to standard care. Video instructions for pessary insertion are available on the Arabin website (https://dr-arabin.de/produkt/arabin-cerclage-pessary-perforated/?lang=en). There are no prohibited cotreatments. A trained member of the local research team will insert the pessary.

### Comparator

The comparator is standard care. There are no prohibited cotreatments.

### Outcomes (women recruited to screening and treatment phase)

There are two primary outcomes: an obstetric and a neonatal primary outcome.

- Obstetric primary outcome: all births before 34+0 weeks following the spontaneous onset of labour. Preterm prelabour rupture of membranes <34 weeks with or without contractions will also be included in this definition of spontaneous onset of labour.^13^ Iatrogenic delivery due to maternal or fetal conditions will not be considered to fulfil the criteria for the primary outcome.
- Neonatal primary outcome: the primary neonatal outcome is a composite of adverse outcomes including stillbirth or neonatal death, periventricular leukomalacia, early respiratory morbidity (defined as any need for supplemental oxygen >30%, continuous positive airway pressure (CPAP) or intratracheal ventilation or surfactant replacement therapy within the first week of life), intraventricular haemorrhage, necrotising enterocolitis and proven sepsis all measured up to 28 days after the expected date of delivery.

Obstetric

Key outcomes

- Mean gestation at delivery.
- Adverse events: infection and cervical trauma.
- Incidence of all births before 37+0 weeks’ gestation.
- Acceptability of the pessary as determined by participant questionnaire.
- Experience of pessary removal.

Other outcomes

- Incidence of all births before each of 28+0, 32+0, and 34+0 weeks’ gestation.
- Preterm birth before 34 weeks’ gestation preceded by preterm premature membrane rupture.
- Incidence of all births before 28, 32, 34 and 37 weeks preceded by the spontaneous onset of labour.
- Method of delivery (in three categories: spontaneous vaginal delivery or vaginal breech, forceps or ventouse and caesarean section).
- Duration of labour overall and of each of the first and second stages of labour.
- Duration of stay in hospital.
- Other adverse events including haemorrhage, tachycardia, vaginal injury and other trauma.
- Serious maternal adverse events up to 28 days after discharge from the hospital.

Key neonatal secondary outcomes

- Incidence of each of the individual components of the primary neonatal outcome.
- Median weight (g) of the newborn at birth.
- Death of live-born babies within the first 28 days after birth.
- Discrete episodes of bloodstream or central nervous system (CNS) infection (positive blood or cerebro spinal fluid (CSF) culture).
- Within first 72 hours.
- Between 72 hours and discharge.

Other neonatal outcomes

- Birth weight centile (for gestation) within 4 weeks after expected date of delivery.
- Death of live-born babies within the first 28 days after estimated date of delivery (EDD).
- Cord pH.
- Apgar score at 1 min and 5 min.
- Need for resuscitation.
- Need for surfactant administration.
- Bronchopulmonary dysplasia.
- Retinopathy of prematurity (stage 1 or more).
- Retinopathy of prematurity requiring surgery (laser or cryotherapy).
- Necrotising enterocolitis (medical or surgical treatment of confirmed cases).
- Twin to twin transfusion (Quintero stage 1 or more).
- Days of respiratory support (either mechanical ventilation or CPAP).
- Days of oxygen therapy.
- Daily level of care.
- Seizures requiring therapy.
- Hyperbilirubinaemia requiring exchange transfusion.
- Periventricular leukomalacia/echogenicity.

Other outcomes include:

- Acceptability and experience of the pessary as determined by participant questionnaire issued at 36 weeks’ gestation.
- The joint distribution of the cumulative hospital costs and the primary clinical outcome.
- Cervical length measurements for all participants to determine the likelihood ratios for short cervix and spontaneous preterm birth.

We will also collect information on use of the following therapies to allow sensitivity analyses in future individual patient data meta-analyses:

- Progestogens (Intramuscular, IM or vaginal) after 16 weeks’ gestation.
- Cervical suture postrandomisation.
- Tocolytics.
- Steroids.
- Magnesium sulfate.
- Antibiotics.
- Arabin pessary in the screened-only women.

### Outcomes (women recruited to screening phase only)

For women recruited to the screening phase but who are not randomised, we will collect maternal and neonatal outcomes as described above. We will not collect information on cotreatments (other than those described above), adverse events, acceptability or costs.

### Criteria for discontinuing intervention

Participation is voluntary. A woman has the right to discontinue or withdraw at any time for any reason. The investigator also has the ability to discontinue a participant. The reason and circumstances will be documented in the eCRF. Unless a participant asks to completely withdraw from the study, those who discontinue treatment will be asked to continue to participate in study visits to facilitate analysis under the intention to treat principle.

If treatment is suspended or stopped due to a serious adverse reaction, the local PI will arrange for follow-up visits or telephone calls until the event has resolved or stabilised or until the study has ended, whichever is sooner. In order to avoid bias and/or missing adverse events, outcome data will be collected (including data from the patient record) and used in the analysis.

We will note whether participants have adhered to the treatment assignation. In the intervention arm, we will define adherence as women who have a pessary in situ at 28 weeks’ gestation (whether it has been out and been reinserted during that time). Women who deliver before 28 weeks will count as adherent if they had their pessary until 7 days before delivery. Those in the standard care arm will be considered adherent unless they had a pessary inserted.

### Visits

Study visits will be conducted at approximately 4 weekly intervals (plus or minus 1 week) following randomisation until 36 weeks, when the pessary will be removed. The study visits will be carried out on those randomised to the pessary and those undergoing standard treatment. Study visits could be face to face or by phone or other means of communication, for example, via letter or email. Pregnancy and fetal well-being will be reviewed at these visits. Additionally, we will collect the following outcomes:

- Any pregnancy complications (adverse events) including bleeding.
- Experience of the pessary during the previous 4 weeks (if applicable).
- Hospital admissions (serious adverse events) – reasons and duration.

### Pessary removal

The pessary will be removed at 35^+0^–36^+6^ weeks’ gestation. The participant will be asked to complete a short questionnaire about her experience and (if appropriate) of having the pessary. For those allocated to pessary treatment, a vaginal examination will be conducted, and the pessary will be removed. Information will be collected on the ease with which removal was achieved. If the pessary is removed before 35 weeks, we will record the indication.

### Ancillary and post-trial care

Care of participants in parallel to and beyond the trial will be provided within the UK National Health service. There will be no no-fault compensation for those who suffer harm from trial participation.

### Qualitative data

A nested qualitative study involving pregnant women will explore their views and experiences of methods of recruitment and the consent process, their understanding and expectations of trial participation, including randomisation and the screening component, and experiences of the intervention. We will interview women who:

- Consented, were screened but found to have a long cervix (up to 10).
- Consented, were screened, have a short cervix and were randomised and allocated to the control group (up to 10).
- Consented, were screened, have a short cervix and were randomised and allocated to the treatment group (up to 20).

Up to 15 interviews will be conducted with a range of healthcare professionals involved in the trial in order to explore their views and experiences of the process.

### Baseline characteristics (screening and treatment phase)

In addition to the outcomes, we will collect the following baseline characteristics on all women:

Age

Height

Weight

Cervical length

Current smoking

Current alcohol

Obstetric history: parity, miscarriage

Medical conditions: hypertension, Insulin dependent diabetes, respiratory disease, cardiac disease

Neurological disease, skin condition, thrombophilia, current pregnancy

Fetal anomaly scan: twin 1, twin 2 (whether performed and the result)

Amniocentesis: twin 1, twin 2 (whether performed and the result)

Data collection forms are available on the STOPPIT-2 website at https://w3.abdn.ac.uk/hsru/STOPPIT2/Public/DownloadPage.aspx.

### Consent

Written informed consent will be obtained from all participating women. Model information sheet and consent forms are available on the study website at https://w3.abdn.ac.uk/hsru/STOPPIT2/Public/DownloadPage.aspx. Women will be given information about the study by the investigator team at each study site, invited to consider participation and will be given adequate time (at least 24 hours) to read the information sheet. If the woman waives this opportunity but still wishes to participate, consent may be taken after a shorter time. Details of the consent process, including the name of the doctor confirming eligibility and the member of the study team taking consent will be recorded in the woman’s notes and in the eCRF. Women undergoing ultrasound measurement of the cervix will be asked to complete a consent form prior to the procedure, which will cover both screening and treatment phases of the study. This will minimise inconvenience to the women (reducing the number of visits required to the hospital) and minimise the time undergoing vaginal examination. We will seek permission for future long-term follow-up of the women and babies via record linkage into national databases (health, social and educational) although such follow-up is beyond the scope of the study described here.

### Data analysis (screening phase data)

We will use cervical length measurements at baseline screening, gestation at delivery, whether delivery was preceded by spontaneous onset of labour and/or preterm premature membrane rupture to determine positive and negative likelihood ratios for spontaneous preterm birth before 34 weeks for a variety of cervical length thresholds. Women treated with the pessary will be excluded from this analysis.

### Data analysis (treatment phase data)

The statistical analysis will be according to the intention to treat principle; all participants will remain in their allocated group for analysis. Statistical significance will be at the 5% level with corresponding 95% CIs derived. Randomised groups will be described at baseline and follow-up using mean (SD), median (IQR) and counts (with percentages) where appropriate.

For the primary obstetric and neonatal outcomes, logistic regression with a fixed effect for the minimisation covariate chorionicity and a random effect for centre will be used to obtain the ORs of the treatment effect, along with the 95% CI and associated p value. In the case of the composite neonatal outcome, the components will also be reported individually to show which elements are driving the primary outcome. In the case of skewed recruitment resulting in small centres, a regional effect will be considered in place of a centre effect.

Continuous secondary outcomes will be analysed using linear regression, adjusting for chorionicity. Binary categorical secondary outcomes will be analysed using logistic regression as per the primary outcomes.

Secondary outcomes with more than two categories will be analysed using multinomial logistic regression.

Analysis of fetal outcomes will allow for clustering within twins by fitting mother as a random effect in a mixed effects logistic regression model.

We will undertake predefined subgroup analyses of the primary outcome by monochorionicity, cervical length (≤25 mm and ≤28 mm). In the subgroup analyses, statistical significance will be at the 1% level with corresponding 99% CIs.

It is not anticipated that primary outcome data will be missing. If data are missing, this is likely to be due to a miscarriage, stillbirth, neonatal or maternal death and therefore it will not be imputed.

A post hoc comparison will also be performed between participants who had membrane rupture and those who did not.

A per-protocol analysis (including those in the intervention arm who are adherent, and excluding those in the control arm who have a pessary inserted) will also be performed.

No formal interim analyses are planned.

The statistical analysis plan is included as online supplementary appendix 1 to this paper.

10.1136/bmjopen-2018-026430.supp1 Supplementary file 1

### Data management

The primary source of data arise from medical notes. These data are entered directly from source, by study site staff, into the eCRF. The eCRF has been developed in accordance with CHaRT software development standards at the University of Aberdeen. Data entry is carried out remotely by authorised study personnel. Accuracy of entry and the storage of source documentation is the responsibility of site study personnel. Validation checks will be set up and run in real time on the eCRF. Checks will include missing data, out of range values, illogical entries and invalid responses. Users will be informed of data issues when the eCRF page is saved providing users with the opportunity to correct the data immediately, where possible. The eCRF system will generate a missing data query for all items not completed at the time. Cross-form checks comparing data items across different forms will be created, where possible or written as reports.

### Data analysis (health economic data)

The economic component will consider the joint distribution of the cumulative hospital costs and the primary clinical outcome from the perspective of the UK NHS. Generalised linear models will be used to estimate the costs between the two treatment groups. The cost estimates and primary clinical outcome will be combined within a cost-effectiveness analysis to calculate the incremental cost per preterm birth prevented. The length of stay distributions will be valued using a per diem unit cost derived from NHS reference costs.^13^ Monetary values will be attached to the labour costs for healthcare practitioners associated with each treatment pathway using standard NHS pay and price estimates.^14 15^ No discounting of hospital costs and clinical outcomes will be undertaken for the within-trial economic component. Flexible parametric regression models will be used to explore the dependency between cost and the primary clinical outcome as well as heterogeneous treatment effects within a sensitivity analysis to inform a decision-analytic model. A decision model will be developed to estimate the longer term resource consequences and health outcomes of preterm birth following twin pregnancy. Model parameter uncertainty will be addressed using probabilistic sensitivity analysis summarised using the cost-effectiveness acceptability curve at differing willingness to pay thresholds.

### Data analysis (qualitative research)

The nested qualitative interview study will be analysed using an inductive cross-sectional thematic approach described by Mason.^16^ This approach involves applying indexing coding to the entire data set providing the means to identify patterns and themes in the data from each arm of the study. The use of qualitative software will also help to facilitate a collaborative approach to the analysis thereby ensuring consistency and rigour.^17^ This method has been chosen as it will help to identify how women experience all aspects of the study in the context of their current pregnancy that builds up a descriptive and conceptual interpretation of the findings that maintains the integrity of each research participant’s accounts. The same analytical approach will be taken for the data generated through interviews with healthcare professionals.

### Study monitoring

On site, trial monitoring will not be conducted on behalf of the cosponsors. Remote monitoring to verify eligibility, consent, staff training and data entry quality will be performed. The research involves the use of a CE marked device used for its intended purpose. Audits may be carried out by individual site R&D departments as per the Research Governance Framework; these audits are independent of the sponsors.

A trial steering committee (TSC) and a data monitoring committee (DMC) will be appointed. The membership and charter for each is included in online supplementary appendix 2 and supplementary appendix 3. The committees will meet not less than annually, or more often if issue arises. There are no formal stopping rules: the TSC and DMC charters state that ‘The TSC, based on recommendations from the DMC, may recommend early termination of the trial or modification of the study design in the event of a clear accumulating data or on the basis of information available from other sources or on safety grounds’. ‘Recommendations to amend the protocol or conduct of the study made by the DMC will be considered and either accepted or rejected by the TSC. The TSC will be responsible for deciding whether to continue or to stop the trial based on the DMC recommendations’.

10.1136/bmjopen-2018-026430.supp2 Supplementary file 2

10.1136/bmjopen-2018-026430.supp3 Supplementary file 3

### Confidentiality of data

All records such as laboratory specimens, questionnaires and evaluation forms will be identified in a manner designed to maintain participant confidentiality. All records will be kept in a secure storage area with limited access. Collection of participant identifiers is performed only to ensure compliance with governance procedures (eg, confirmation of informed consent). Paper records with identifying information will be kept in a locked cabinet with limited access. Electronic identifying information will be kept in a separate trial database and will not be revealed as part of routine data analysis or in any data sharing exercise. Clinical information will not be released without the written permission of the participant. The investigator and study site staff involved with this study will not disclose or use for any purpose other than performance of the study, any data, record or other unpublished, confidential information disclosed to those individuals for the purpose of the study. Prior written agreement from the sponsor or its designee must be obtained for the disclosure of any said confidential information to other parties.

### Data protection

All Investigators and study site staff involved with this study must comply with the requirements of the Data Protection Act 1998 and subsequent General Data Protection Regulations 2018 with regard to the collection, storage, processing and disclosure of personal information and will uphold the Act’s core principles. Access to collated participant data will be restricted to those clinicians treating the participants, representatives of the sponsor(s) and representatives of regulatory authorities. Computers used to collate the data will have limited access measures via user names and passwords.

Published results will not contain any personal data that could allow identification of individual participants.

### Sample size calculation

We plan to recruit 2500 women to the SCREENING phase and randomise 500 women in the treatment phase. We anticipate that around 58 sites (largely UK NHS sites) will be involved.

Women with a short cervix are at higher risk of preterm birth and may benefit most from pessary treatment. We aim to treat women with a cervical length ≤35 mm, which we believe to be around the 30th centile. Assuming this to be correct, a sample size of 1850 screened was initially identified to generate 555 eligible for randomisation in the treatment phase. However, following reanalysis of data (masked to treatment allocation) in September 2017 after 29 months of screening (with data on 1214 women screened), we estimated that to randomise 550 women, the sample size of screening needed to increase to 2500. Although we will encourage women to participate in the screening phase only if they wish also to participate in the treatment phase, we have allowed for a further 10% drop out after screening, so that we now aim to randomise 500 women in the treatment phase.

We plan to randomise 500 women (250 in each group). We assume a relative risk of the primary obstetric outcome (spontaneous preterm labour leading to preterm birth before 34 weeks) in the pessary group of 0.6. We believe our relative risk reduction is conservative, given a relative risk of 0.49 for delivery before 32 weeks^7^ and 0.47 for delivery before 34 weeks (21% vs 42%) (S Liem, personal communication, 2013) in the ProTwin study. We anticipate that 35% of women in the control group will deliver before 34 weeks. We believe that this is a conservative estimate, given that a systematic review indicated that 34.9% of women with a cervical length of 35 mm or less (when scanned at 20 weeks’ gestation) will deliver preterm before 32 weeks.^12^ Assuming a baseline rate of 35% and a relative risk of 0.6, a sample size of 500 has 94% power to detect a difference at the 5% significance level. If the preterm birth rate before 34 weeks is only 30%, the power drops to 88%. Both allow for losses to follow-up and imperfect compliance.

For the primary neonatal outcome, Liem showed an effect size of 0.42 at the child level and an incidence of 24% in the control group.^7^ We have powered our study for a relative risk of 0.6 for the primary neonatal outcome. Assuming prevalence rates as in Liem, our study would have 97% power. In practice, our postscreening groups of women with cervical length ≤35 mm is probably a lower risk than in the Liem group (less than 38 mm), given comparisons of the rate of preterm delivery in each of the control groups. Hence, if we assume a lower rate of the neonatal primary outcome of (say) 18%, we still have 88.4% power to detect a relative risk of 0.6 in the Arabin pessary group. Such a calculation assumes analysis at the child level is appropriate for the neonatal outcome.

For the subgroup of women with cervical length of ≤25 mm, the anticipated rate of the primary obstetric outcome in the control group is 82/159 (51%).^11^ The study has 85% power to detect a relative risk of 0.6 in this group, with a sample size of 234 (25 mm was the 14th centile in the To *et al*).

### Patient and public involvement (PPI)

The importance of the research question was identified by two major groups, both involving PPI.

The first was the NICE guideline on multiple pregnancy, published in 2011, which had embedded PPI.

The guideline generated the following research recommendation: ‘What interventions are effective in preventing spontaneous preterm birth in women with twin and triplet pregnancies, especially in those at high risk of preterm birth? The second group was the Clinical Study Group from the RCOG, which has extensive lay and patient involvement. ‘The use of the Arabin pessary for preventing preterm birth’ is one of five research priorities identified in late 2012.

There was PPI in the development of the protocol at grant submission stage, including in choice of the outcome measures. Specifically, the CEO of a charity focusing on preterm birth (Jane Denton, Multiple Births Foundation) who was a coapplicant on this project was and involved in the study design.

Patients and the public are also involved in the TSC for this study, which oversaw the conduct of the study: both through participation of individual patients and through participation of Keith Read from Tamba (The Twins and Multiple Births Association) and two individual patients. Although PPI were not directly involved in individual patient recruitment, they provided helpful comments on the patient information sheet used for this purpose. PPIs were members of the ethics committee who assessed the research burden associated with participating in this study.

Individual study participants will be sent a summary of study findings, when the main study is published. We also make use of our connections with the charities Tommy’s, the Multiple Births Foundation and the Twins and Multiple Births Association to disseminate information.

### Dissemination

A study report will be prepared in accordance with Good clinical practice (GCP) guidelines and submitted to the funder National institute for health research (NIHR) for publication in the NIHR journals library. A summary report will be submitted for publication in a peer review journal. Data will be used for publication and presentation at scientific meetings. Authorship guidelines are attached at online supplementary appendix 4.

10.1136/bmjopen-2018-026430.supp4 Supplementary file 4

### Access to data

The chief investigator (JEN) and all study statisticians (JN, GM and DC) will have full access to the final dataset data during analysis. Access will be provided to any other author on request. There are contractual limitations on disclosures.

### Data deposition

We aim to deposit the data in a shared depository. We do not anticipate providing access to the statistical analysis code.

### Protocol amendments

Version 1 of the STOPPIT 2 protocol dated 22 July 2014 was approved by the ethics committee on 29 August 2014. A list of protocol amendments is included in an online supplementary appendix 5. It is not anticipated that any further substantive amendments will be made, other than addition or closure of sites. Protocol amendments are notified to and approved by the sponsor, funder and research ethics committee and communicated to investigators after approval. The most recent approved version of the protocol is version 4, dated 10 January 2018. One final amendment of the protocol will be submitted to address minor updates made during the preparation of this protocol paper.

10.1136/bmjopen-2018-026430.supp5 Supplementary file 5

## Supplementary Material

Reviewer comments

Author's manuscript
