# Supplementary material for: Open randomised trial of the (Arabin) pessary to prevent preterm birth in twin pregnancy with health economics and acceptability: STOPPIT-2—a study protocol
Source: BMJ Open. 2018 Dec 6;8(12):e026430. doi: 10.1136/bmjopen-2018-026430 (PMC6286620; doi:10.1136/bmjopen-2018-026430)
Supplement: Supplementary file 1 [file bmjopen-2018-026430supp001.pdf]

STOPPIT-2

An open randomised trial of the Arabin pessary to prevent preterm birth in twin pregnancy, with health economics and acceptability – STOPPIT-2.

Statistical Analysis Plan
Treatment Phase
Version 1.0

Prepared by:

David Cooper

(CHaRT Statistician)

Signed: David J. Cooper

Date: 30.8.2018

Approved by:

Professor Jane Norman

(Chief Investigator)

Signed: [signature]

Date: 30th August 2018

Protocol version 5 23/8/2018

Contents

1 Study Objectives

1.1 Primary objective

1.2 Secondary objective

2 Study Design

2.1 Randomisation and blinding

3 Outcome Measures

3.1 Primary outcome

3.2 Secondary outcomes

3.3 Timing of outcome measurements

3.4 Adverse events

4. Sample Size and Power Calculations

5. Statistical Methods

5.1 Primary outcome

5.2 Secondary outcomes

5.3 Subgroup analyses

5.4 Missing data

6. Dummy Tables

7. References

List of Tables

1. Baseline clinical characteristics
2. Experience of device fitting
3. Experience of device through trial
4. Experience of device at removal
5. Primary obstetric outcome of birth before 34+0 weeks – overall and by subgroup of chorionicity and cervical length
6. Secondary obstetric outcomes – gestational age at delivery, duration of labour and duration of stay in hospital
7. Secondary obstetric outcomes – method of delivery, incidence of births before 28+0, 32+0, 34+0 and 37+0 weeks, adverse events
8. Primary composite neonatal outcome and incidence of individual components
9. Secondary neonatal outcomes – continuous outcomes
10. Secondary neonatal outcomes – categorical outcomes
11. Safety issues

1. Study Objectives

1.1 Primary objective

The primary objective of this study is to test if the Arabin cervical pessary reduces the risk of spontaneous preterm labour leading to preterm birth in women with a cervical length less than or equal to 35mm, who are expecting twins.

1.2 Secondary objectives

The secondary objectives of this study are to use the entire cohort of women undergoing cervical length scanning to identify the profile of cervical length measurement in women with twin pregnancy in the UK. The study also aims to obtain the positive and negative likelihood ratios of spontaneous preterm birth before 34 weeks gestation for a variety of cervical length thresholds. A separate plan will be created to describe this analysis.

2. Study Design

STOPPIT-2 is a multicentre, open label, randomised controlled trial of the Arabin pessary versus standard treatment (no additional treatment other than what would normally be received) in women with twin pregnancy.

The study has two phases:-
A *screening* phase in which women with a short cervix (cervical length of ≤ 35mm) will be identified;
A *treatment* phase in which women with a short cervix will be randomised to treatment with Arabin pessary or standard treatment.
Women will be seen in the antenatal clinic setting.

2.1 Randomisation and blinding

Randomisation will be carried out between 18 weeks + 0 days gestation and 20 weeks + 6 days gestation. Women with a short cervix (≤ 35mm) who agree to participate in the treatment phase of the study will be randomised to either treatment with the Arabin cervical pessary or to standard care (no additional treatment). Randomisation will be performed by the central randomisation facility based within CHaRT.

Randomisation will be minimised with a random element rather than stratified given the properties of minimisation in ensuring comparability between the groups. The minimisation variables are centre and chorionicity (monochorionic or dichorionic).

Neither the participant nor the investigator are blinded to which treatment has been allocated. The investigator will inform the woman of treatment allocation after randomisation.

3. Outcome Measures

3.1 Primary outcomes

- *Obstetric*: all births before 34 weeks + 0 days gestation following the spontaneous onset of labour. Pre-term prelabour rupture of membranes before 34 weeks with or without contractions is also included in the definition of spontaneous onset of labour.
- *Neonatal*: a composite of adverse outcomes including any death from trial entry to 28 days post expected date of delivery (miscarriage, stillbirth, neonatal or postneonatal death), periventricular

leukomalacia, early respiratory morbidity (defined as any need for supplemental oxygen >30%, CPAP [continuous positive airway pressure], or intra-tracheal ventilation or surfactant replacement therapy within the first week of life), intraventricular haemorrhage, necrotizing enterocolitis and proven sepsis. All these will be measured up to 28 days after the expected date of delivery determined by the first trimester ultrasound.

3.2 Secondary outcomes

Obstetric:

Key outcomes

- Mean gestation at delivery
- Adverse events: infection, cervical trauma
- Incidence of all births before 37+0 weeks gestation
- Acceptability of the pessary as determined by participant questionnaire
- Experience of pessary removal

Other outcomes

- Incidence of all births before each of 28+0, 32+0, and 34+0 weeks gestation
- Preterm birth before 34+0 weeks gestation preceded by preterm premature membrane rupture
- Incidence of all births before each of 28+0, 32+0, 34+0 and 37+0 weeks gestation preceded by spontaneous onset of labour
- Method of delivery (in three categories: spontaneous vaginal delivery or vaginal breech, forceps or ventouse, caesarean section)
- Duration of labour overall, and of each of the first and second stages of labour
- Duration of stay in hospital
- Other adverse events including haemorrhage, tachycardia, vaginal injury, other trauma
- Serious maternal adverse events up to 28 days after discharge from the hospital

Key *Neonatal secondary outcomes:*

- Incidence of each of the individual components of the primary neonatal outcome
- Median weight (g) of the newborn at birth
- Death of live born babies within the first 28 days after birth
- Discrete episodes of bloodstream or CNS infection (positive blood or CSF culture)
    - Within first 72 hours
    - Between 72 hours and discharge

Other neonatal outcomes

- Birth weight centile (for gestation) within 4 weeks after expected date of delivery
- Death of live born babies within the first 28 days after EDD
- Cord pH
- Apgar score at 1 and 5 minutes
- Need for resuscitation
- Need for surfactant administration

- Bronchopulmonary dysplasia
- Retinopathy of prematurity (stage 1 or more)
    - Retinopathy of prematurity requiring surgery (laser or cryotherapy)
- Necrotising enterocolitis, (medical or surgical treatment of confirmed cases
- Twin to twin transfusion (Quintero stage 1 or more)

- Days of respiratory support [either mechanical ventilation or CPAP]
- Days of oxygen therapy
- Daily level of care.
- Seizures requiring therapy.
- Hyperbilirubinaemia requiring exchange transfusion.
- Periventricular leukomalacia / echogenicity

4. Sample Size and Power Calculations

We plan to recruit 2500 women to the SCREENING phase and randomise 500 women in the TREATMENT phase. We anticipate that around 58 sites (largely UK NHS sites) will be involved.

We aim to treat women with a cervical length less than or equal to 35mm, which we believe to be around the 30th centile. Assuming this to be correct, a sample size of 1850 women screened was initially identified as the number of women required, to generate 555 women eligible for randomisation in the TREATMENT PHASE. However, re-analysis of data (masked to treatment allocation) in September 2017 after 29 months of screening (with data on 1214 women screened), we estimated that to randomise 550 women, the sample size of screening needed to increase to 2500. Although we will encourage women to participate in the screening phase only if they wish also to participate in the treatment phase, we have allowed for a further 10% drop out after screening, so that we aim to randomize 500 women in the treatment phase.

We plan to randomise 500 women of 500 (250 in each group). We assume a relative risk of the primary obstetric outcome (spontaneous preterm labour leading to preterm birth before 34 weeks) in the Arabin pessary group of 0.6. We believe our relative risk reduction is conservative, given a relative risk of 0.49 for delivery before 32 weeks [20] and 0.47 for delivery before 34 weeks (21% v 42%)(Liem S, personal communication) in the ProTwin study. We anticipate that 35% of women in the control group will deliver before 34 weeks. Again we believe that this is a conservative estimate, given that a systematic review indicated that 34.9% of women with a cervical length of 35mm or less (when scanned at 20 weeks gestation) will deliver preterm before 32 weeks (Kindinger LM et al 2015). Assuming a baseline rate of 35% and a relative risk of 0.6, a sample size of 500 has 94% power to detect a difference at the 5% significance level between the groups. If the preterm birth rate before 34 weeks is only 30%, power drops to 88%. Both allow for losses to follow up and imperfect compliance.
For the primary neonatal outcome, Liem showed an effect size of 0.42 at the child level, and an incidence of 24% in the control group. We have powered our study for a relative risk of 0.6 for the primary neonatal outcome. Assuming prevalence rates as in Liem, our study would have 97% power. In practice, our post screening groups of women with cervical length less than or equal to 35mm group is probably at somewhat lower risk than the Liem less than 38mm group (given comparisons of the rate of preterm delivery in each of the control groups). Hence if we assume a lower rate of the neonatal primary outcome of (say) 18% we still have 88.4% power to detect a relative risk of 0.6 in the Arabin pessary group. Such a calculation assumes analysis at the child level is appropriate for the neonatal outcome.

For the subgroup of women with cervical length of less than or equal to 25mm, the anticipated rate of the primary obstetric outcome in the control group is 82/159 (51%) [10]. The study has 85% power to detect a

relative risk of 0.6 in this group, with a sample size of 234 (25mm was the 14th centile in the To et al) 0.48 x sample size in < 30mm group).

5. Statistical Methods

The primary statistical analysis will be according to the intention to treat principle in which all participants will remain in their allocated group for analysis. An additional per protocol analysis will also be performed.

Statistical significance will be at the 5% level with corresponding 95% confidence intervals (CI) derived.

Randomised groups will be described at baseline and follow-up using means (with standard deviations), medians (with inter-quartile ranges) and counts (with percentages) where appropriate.

5.1 Primary outcomes

For the primary obstetric and neonatal outcomes, logistic regression with a fixed effect for the minimisation covariate chorionicity and a random effect for centre will be used to obtain the odds ratios of the treatment effect, along with the 95% confidence interval and associated p-value. In the case of the composite neonatal outcome the components will also be reported individually to show which elements are driving the primary outcome. In the case of skewed recruitment resulting in small centres a regional effect will be considered in place of a centre effect.

5.2 Secondary outcomes

Continuous secondary outcomes will be analysed using linear regression, adjusting for chorionicity. Binary categorical secondary outcomes will be analysed using logistic regression as per the primary outcomes.

Secondary outcomes with more than two categories will be analysed using multinomial logistic regression.

Analysis of fetal outcomes will allow for clustering within twins by fitting mother as a random effect in a mixed effects logistic regression model.

5.3 Subgroup analyses

Predefined subgroup analyses of the primary outcome by monochorionicity (no or yes), by cervical length (≤ 25mm) and also by cervical length ≤ 28mm will be undertaken.

A post-hoc comparison will also be performed between participants who had membrane rupture and those who did not.

In the subgroup analyses statistical significance will be at the 1% level with corresponding 99% confidence intervals (CI).

5.4 Missing Data

It is not anticipated that primary outcome data will be missing and if it is this is likely due to a miscarriage, stillbirth, neonatal or maternal death and therefore it will not be imputed.

6. Dummy Tables

Table 1: Baseline characteristics

**Characteristic**	**Level**	**Arabin pessary N =**	**Standard treatment N =**
Age	Mean (sd)		
	Min-Max		
Height	Mean(sd)		
	Min-Max		
Weight	Mean (sd)		
	Min-Max		
Cervical length	Mean (sd)		
	Min-Max		
Current Smoking	Yes, n (%)		
Current alcohol	Yes, n (%)		
Obstetric History			
Parity	>0, n (%)		
Miscarriage	>0, n (%)		
Medical conditions	>0, n (%)		
Hypertension	Yes, n (%)		
Insulin dependent diabetes	Yes, n (%)		
Respiratory disease	Yes, n (%)		
Cardiac disease	Yes, n (%)		
Neurological disease	Yes, n (%)		
Skin condition	Yes, n (%)		
Thrombophilia	Yes, n (%)		
Current Pregnancy			
Fetal anomaly scan: Twin 1	Normal – n (%)		
	Defined abnormality – n (%)		
	Uncertain abnormality – n (%)		
	Not done – n (%)		
Fetal anomaly scan: Twin 2	Normal – n (%)		
	Defined abnormality – n (%)		
	Uncertain abnormality – n (%)		
	Not done – n (%)		
Amniocentesis: Twin 1	Abnormal – n (%)		
Amniocentesis: Twin 2	Abnormal – n (%)		

Table 2: Experience of device fitting

**Arabin pessary**<br>**N =**	**At fitting**
Maternal experience of having device fitted – n (%)	
Painless	
Slightly uncomfortable	
Uncomfortable	
Very uncomfortable	
Worst pain imaginable	
Too painful to allow insertion	
Clinical team experience of fitting device – n (%)	
Easy	
Moderately easy	
Neither easy nor difficult	
Difficult	
Very difficult	
Impossible	

Table 3: Experience of device through trial

Arabin pessary<br>N =	36 week questionnaire
I could feel the pessary – n(%)	
Never	
A few times	
At least once every week	
Every day	
All the time	
I found the pessary uncomfortable – n(%)	
Never	
A few times	
At least once every week	
Every day	
All the time	
I found the pessary painful – n(%)	
Never	
A few times	
At least once every week	
Every day	
All the time	
I had vaginal discharge – n(%)	
Never	
A few times	
At least once every week	

Every day<br>All the time<br><br>*I had vaginal bleeding – n(%)*<br>Never<br>A few times<br>At least once every week<br>Every day<br>All the time<br><br>*I had to use panty liners/sanitary towels because of the vaginal discharge/bleeding– n(%)*<br>Never<br>A few times<br>At least once every week<br>Every day<br>All the time<br><br>*The vaginal discharge was – n(%)*<br>The same as before the pessary<br>A little more than before the pessary<br>A lot more than before the pessary<br><br>*The vaginal bleeding was – n(%)*<br>The same as before the pessary<br>A little more than before the pessary<br>A lot more than before the pessary<br><br>*The vaginal discharge was – n(%)*<br>The same as in my last pregnancy<br>A little more than in my last pregnancy<br>A lot more than in my last pregnancy<br>This is my first pregnancy<br><br>*The vaginal bleeding was – n(%)*<br>The same as in my last pregnancy<br>A little more than in my last pregnancy<br>A lot more than in my last pregnancy<br>This is my first pregnancy<br><br>*Any other side effects experienced with the pessary*	

Table 4: Experience of device removal

	**At removal**
Maternal experience of having device removed– n (%)<br>Painless<br>Slightly uncomfortable<br>Uncomfortable<br>Very uncomfortable	

Worst pain imaginable<br><br>*Clinical team experience of removing device – n (%)*<br>Easy<br>Moderately easy<br>Neither easy nor difficult<br>Difficult<br>Very difficult	

Table 5: Primary obstetric outcome of birth before 34+0 weeks – overall and by subgroup of chorionicity and cervical length

**Outcome**	**Arabin pessary N =**	**Standard treatment N =**	**Odds ratio (95% CI)**	**p-value**
All pregnancies				
Proportion of women delivering before 34 weeks – n (%)				*
Monochorionic pregnancy and cervical length ≤ 25mm				
Proportion of women delivering before 34 weeks – n (%)				
Dichorionic pregnancy and cervical length ≤ 25mm				
Proportion of women delivering before 34 weeks – n (%)				
Monochorionic pregnancy and cervical length ≤ 28mm				
Proportion of women delivering before 34 weeks – n (%)				
Dichorionic pregnancy and cervical length ≤ 28mm				
Proportion of women delivering before 34 weeks – n (%)				

* refers to p-value for proportion in Arabin pessary versus standard treatment group from logistic regression analysis adjusting for chorionicity

Table 6: Secondary obstetric outcomes – gestational age at delivery, duration of labour and duration of stay in hospital

**Outcome – mean (sd)**	**Arabin pessary N =**	**Standard treatment N =**	**Mean difference (99% CI)**	**p-value ***
Gestational age at delivery (weeks)				
Duration of labour stage 1 (mins)				
Duration of labour stage 2 (mins)				
Duration of labour overall (mins)				
Duration of hospital stay (days)				

* refers to p-value for proportion in Arabin pessary versus standard treatment group from linear regression analysis adjusting for chorionicity

Table 7: Secondary obstetric outcomes – method of delivery, incidence of births before 28+0, 32+0, 34+0 and 37+0 weeks, adverse events

**Outcome – n (%)**	**Arabin pessary N =**	**Standard treatment N =**	**Odds ratios (99% CI)**	**p-value ***
Method of delivery				
Spontaneous vaginal delivery				
Vaginal breech				
Forceps or ventouse				
Caesarian section				
Incidence of all births				
Before 28+0 weeks				
Before 32+0 weeks				
Before 34+0 weeks				
Before 37+0 weeks				
Incidence of births – preceded by spontaneous onset of labour				
Before 28+0 weeks				
Before 32+0 weeks				
Before 34+0 weeks				
Before 37+0 weeks				
Incidence of birth before 34+0 weeks preceded by PPMR				
Adverse events				
Infection				
Haemorrhage				
Tachycardia				
Significant maternal adverse event up to 28 days after discharge from hospital				

PPMR – preterm premature membrane rupture
* refers to p-value for proportion in Arabin pessary versus standard treatment group from logistic regression analysis adjusting for chorionicity or using proportional odds analysis

Table 8: Primary composite neonatal outcome and incidence of individual components

**Outcome – n (%)**	**Arabin pessary N =**	**Standard treatment N =**	**Odds ratio (95% CI)**	**p-value**
Composite neonatal outcome				
Individual components				
Stillbirth or neonatal death				
Periventricular leukomalacia				
Early respiratory morbidity				
Intraventricular haemorrhage				
Necrotizing enterocolitis				
Proven sepsis				

Data refer to all twins with the 95% CI and p-values adjusted for clustering within twins

Table 9: Secondary neonatal outcomes – continuous outcomes

**Outcome – mean (sd)**	**Arabin pessary N =**	**Standard treatment N =**	**Mean difference (99% CI)**	**p-value**
Birth weight centile within 4 weeks after expected d.o.d.				
Weight of newborn at birth				
Cord pH				
Agpar score at 1 minute				
Agpar score at 5 minutes				
Days of oxygen therapy				
Level of care days				

d.o.d. – date of delivery
Data refer to all twins with the 99% CI and p-values adjusted for clustering within twins

Table 10: Secondary neonatal outcomes – categorical outcomes

**Outcome – n (%)**	**Arabin pessary N =**	**Standard treatment N =**	**Odds ratio (99% CI)**	**p-value**
Need for resuscitation				
Fetal or neonatal death within the first 28 days after birth				
Need for surfactant administration				
Bronchopulmonary dysplasia				
Necrotising enterocolitis				
Discrete episodes of bloodstream or CNS infection				
Daily level of care				
Rate of major adverse neonatal outcomes before discharge from hospital				

Data refer to all twins with the 99% CI and p-values adjusted for clustering within twins

Table 11: Safety Issues – p-value from Fisher's Exact test on subjects

**Type**	**Arabin pessary N =**	**Standard treatment N =**	**p-value from exact test**
Mother died			
Intrauterine death			
Neonatal death			
Involved or prolonged inpatient maternal hospitalisation			
Involved persistent/significant maternal disability/incapacity			
Life threatening			
Chorioamnionitis or intrauterine infection			
Congenital anomaly/birth defect			

7. REFERENCES

1. To, M. S., E. B. Fonseca, F. S. Molina, A. M. Cacho and K. H. Nicolaides (2006). "Maternal characteristics and cervical length in the prediction of spontaneous early preterm delivery in twins." American journal of obstetrics and gynecology 194(5): 1360-1365.
2. Liem S et al (2013). “Cervical pessaries for prevention of preterm birth in women with a multiple pregnancy (ProTWIN): a multicentre, open-label randomised controlled trial”. The Lancet 382(9901): 1341-1349.
3. Norman, J. E., F. Mackenzie, P. Owen, H. Mactier, K. Hanretty, S. Cooper, A. Calder, G. Mires, P. Danielian, S. Sturgiss, G. MacLennan, G. Tydeman, S. Thornton, B. Martin, J. G. Thornton, J. P. Neilson and J. Norrie (2009). "Progesterone for the prevention of preterm birth in twin pregnancy (STOPPIT): a randomised, double-blind, placebo-controlled study and meta-analysis." Lancet 373(9680): 2034-2040.
4. Kindinger LM, Poon LC, Cacciatore S, MacIntyre DA, Fox NS, Schuit E, Mol B, Liem S, Lim A, Serra V, Perales A, Hermans F, Darzi A, Bennett P, Nicolaides KH, Teoh TG (2015). The prediction of preterm birth in twin pregnancy: an individual patient level meta-analysis. BJOG in press.
