# Supplementary material for: Open randomised trial of the (Arabin) pessary to prevent preterm birth in twin pregnancy with health economics and acceptability: STOPPIT-2—a study protocol
Source: BMJ Open. 2018 Dec 6;8(12):e026430. doi: 10.1136/bmjopen-2018-026430 (PMC6286620; doi:10.1136/bmjopen-2018-026430)
Supplement: Supplementary file 2 [file bmjopen-2018-026430supp002.pdf]

STOPPIT-2 study

Trial Steering Committee Charter

Version 2.0 Date 22nd July 2015

**Study Title:**	An open randomised trial of the Arabin pessary to prevent preterm birth in twin pregnancy, with health economics and acceptability – STOPPIT-2.
**Chief Investigator:**	**Professor Jane Norman**
**REC Number:**	**14/SS/031**
**Sponsor:**	University of Edinburgh & NHS Lothian

Approval Signatures:

The following individuals, by providing their signatures, indicate their understanding of and willingness to comply with the roles and responsibilities assigned to them in this Charter.

1. TSC independent Chair:

________________	________________	_ _ / _ _ /_ _ _ _
PRINT NAME	SIGNATURE	DATE

2. TSC independent Member:

________________	________________	_ _ / _ _ /_ _ _ _
PRINT NAME	SIGNATURE	DATE

3. TSC independent Member:

________________	________________	_ _ / _ _ /_ _ _ _
PRINT NAME	SIGNATURE	DATE

4. Chief Investigator:

________________	________________	_ _ / _ _ /_ _ _ _
PRINT NAME	SIGNATURE	DATE

5. Trial Statistician:

________________	________________	_ _ / _ _ /_ _ _ _
PRINT NAME	SIGNATURE	DATE

CONTENTS

CONTENTS........ 1
1. Introduction 3
2. Membership of the TSC........ 3
2.1. Members:........ 4
2.2. Agreements 6
2.3. Indemnity........ 6
2.4. Payments........ 6
3. Responsibilities 6
4. Interaction between TSC and other study committees........ 8
5. Scheduling, Quorum, and Organization of Meetings........ 8
5.1. Attendance at meetings 9
5.2. Non-attendance at meetings........ 9
5.3. Report for the TSC........ 9
5.4. Contents of the TSC Report........ 10
6. Decision making........ 10
6.1. Recommendations........ 10
6.2. TSC records........ 11
7. Trial closure........ 11
9. Publication........ 12
10. References: 12

Abbreviations and glossary

CI	Chief Investigator
DMC	Data Monitoring Committee
eCRF	Electronic case report form
GCP	Good clinical practice
ISRCTN	International standard randomised controlled trial number
TSC	Trial Steering Committee
PMG	Project Management Group

1. Introduction

The role of the Trial Steering Committee (TSC) is to act as the oversight body for this trial on behalf of the Sponsors and the Trial Funder and to ensure that the trial is conducted according to the guidelines for Good Clinical Practice (GCP), Research Governance Framework for Health and Social Care and all relevant regulations and local policies.

The background to this trial and the trial objectives are described in the protocol. The purpose of this document is to describe the membership, terms of reference, roles, responsibilities, authority, decision-making and relationships of the Trial Steering Committee (TSC) for this trial, including the timing of meetings, methods of providing information to and from the TSC, frequency and format of meetings and relationships with other trial committees. The charter also describes the procedures for ensuring confidentiality and proper communication to and from the TSC and an outline of the content of the reports to be provided to the TSC.

2. Membership of the TSC

The STOPPIT-2 Trial TSC is an independent multidisciplinary group that, collectively, have experience/expertise in the management of patients with condition(s) relevant to study and anticipated adverse effects and in the conduct and monitoring of randomised clinical trials. The role of the TSC is to provide oversight for the trial and provide advice through its independent Chair to the Project Management Group (PMG) on all aspects of the trial.

2.1. Members

(Names redacted from this version)

INDEPENDENT (VOTING) MEMBERS

TSC Independent Chair
Neonatologist

TSC Independent Member
Statistician

TSC Independent Member
Obstetrician

TSC Independent Member:
Charity lead

TSC Independent Member
Patient Advisory Group (PAG) Representative

TSC Independent Member
Patient Advisory Group (PAG) Representative

NON-INDEPENDENT MEMBERS

Representative of the Sponsor (Non-Voting)

Chief Investigator
Professor Jane Norman

The Queen's Medical Research Institute
47 Little France Crescent
Edinburgh
EH16 4TJ
Tel: 0131 242 2694

Email:jane.norman@ed.ac.uk;

The Facilitator (Non-Voting)

Members of the Project Management Group (PMG) who may be invited to attend as non-voting members:

Trial Manager

Trial Statistician

NB: Other members of the Project Management Group (PMG) are invited at the discretion of the TSC Chair.

FUNDERS CONTACT:

Programme manager

2.2. Agreements

TSC independent members will be asked to formally acknowledge their agreement to join the group by signing the TSC charter indicating that they:

(1) agree to be a member of the TSC

(2) agree with the contents of this Charter

(3) and have declared any potential conflicts of interest.

TSC members should not have any apparent financial, scientific or intellectual conflict of interest (see tables 1 and 2) that could prevent them from objectively reviewing the study protocol, interim and final data and giving advice to the PMG. TSC members should disclose to the Chair any other conflicts they consider relevant. Any members who develop significant conflicts of interest during the course of the trial should inform the Chair and may need to consider resigning from the TSC.

2.3. Indemnity

The University of Edinburgh insurance indemnifies TSC members for their work on the committee.

2.4. Payments

Standard travel and subsistence costs only will be paid to members of the TSC. Representatives of the Patient Advisory Group (PAG) may receive payments for their time in attending meetings of the TSC as appropriate to their role as a member of the PAG. No other payments or rewards will be given to members.

3. Responsibilities

The TSC members, on behalf of the Sponsor and Funder, will have overall responsibility for the conduct of the trial and for safeguarding the rights, safety and wellbeing of participants. The Chair has previous experience of serving on trial committees and experience of Chairing meetings, and will facilitate and summarise discussions. Responsibilities of the TSC include:

- Reviewing selection/recruitment/retention of participants and their management.
- Finalising and reviewing study protocol and other study documentation.
- Determine if amendments to the protocol or changes to study conduct are required and deciding on changes to these and to study conduct in general. Any changes to trial documentation or conduct must be notified to the TSC.

- Reviewing adherence to the protocol by Investigators and participants.
- Assessing the impact and relevance of external evidence.
- Assessing integrity and completeness of data collected.
- Monitoring the overall conduct of the trial, ensuring that it follows the standards set out in the guidelines of GCP, assessing the safety and efficacy of the interventions, recruitment figures and completion of trial assessments.
- Reviewing, commenting and making decisions on extension requests.
- Reviewing the recommendations of the DMC (Data Monitoring Committee) (if applicable) and/or other study committees and suggesting appropriate action to the PMG.
- Monitoring the progress of study/trial and deciding on appropriate action in order to maximise the chances of completing it within the agreed timelines.
- Considering new information relevant to the study e.g. results from other studies that may have a bearing to the conduct of the study and deciding on appropriate action.

Additional responsibilities may include:

1. Endorsing the annual report to the funder and ethics (if required).
2. Approving proposed protocol amendments or new trial sub-studies.
3. Approving requests for early release of (subsets of) data.
4. Approving external applications for the use of stored samples (if applicable).
5. Approving presentation of results during the trial or soon after closure.
6. Approving strategies to improve recruitment or follow-up.

4. Interaction between TSC and other study committees

The responsibilities of each trial committee are outlined in the protocol and in the respective Charters. The relationships between these groups are summarised in Figure 1 below.

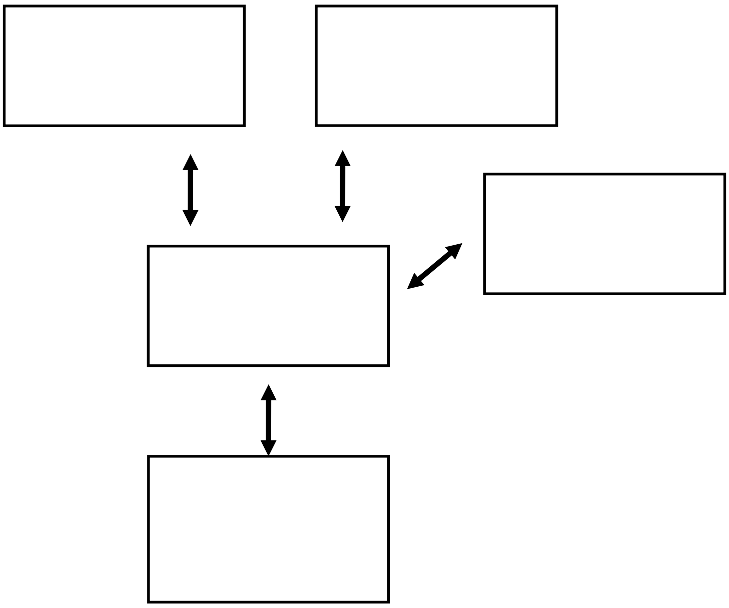

5. Scheduling, Quorum, and Organization of Meetings

The responsibility for calling and organising TSC meetings lies with the Chief Investigator in association with the Chair. The Chair, assisted by the Chief Investigator, is responsible for facilitating the meetings and summarising discussions. The Facilitator will be responsible for the organisation of meetings and should be copied into all communications with and between the TSC.

A meeting of the TSC is considered quorate if at least four independent members, including the Chair (unless otherwise agreed), take part, plus a member of the trial team.

The TSC will aim to meet in person at least yearly. The TSC should be available to provide independent advice as required, not just when meetings are scheduled. Major trial issues may need to be dealt with between meetings, by phone or by email. The TSC should maintain confidentiality of all information it receives. Members should not discuss confidential issues from their involvement in the study until the primary results have been published.

5.1. Attendance at meetings

Meetings will usually be limited to the TSC members (Section 2), observers from the sponsors, invited PMG members and the Facilitator. Other attendees may be invited for all or part of the meeting by the TSC. The observers are not members of the TSC but may be invited to provide expert input; other observers will be at the discretion of the TSC and the Facilitator but may include members of the PMG other than the CI.

Effort will be made to ensure that all members can attend. The Facilitator will work for a date that enables this. The CI must try to attend all meetings, especially if major actions are expected. Members who cannot attend in person should be encouraged to participate by teleconference. TSC members who will not be able to attend the meeting may pass comments to the TSC Chair via the Facilitator for consideration during the discussions. If, at short notice, any TSC members cannot attend then the TSC may still meet if at least four independent members, including the Chair (unless otherwise agreed), will be present, plus also a member of the trial team. If the TSC is considering a major action after such a meeting the TSC Chair should communicate with the absent members, including the CI, as soon after the meeting as possible to check they agree. If they do not, a further teleconference should be arranged with the full TSC.

5.2. Non-attendance at meetings

If an independent member does not attend a meeting or provide comments when requested between meetings, it will be ensured that the independent member is available for the next meeting. If an independent member does not attend the next meeting or provide comments when next requested, they will be asked if they wish to remain part of the TSC. If an independent member does not attend a third meeting, strong consideration will be given to replacing this member.

The TSC membership is for the duration of the trial. If any members leave the TSC, the PMG and TSC should provide recommendations for replacements promptly.

5.3. Report for the TSC

A report will be prepared by the trial manager on behalf of the PMG containing accrual and any matters affecting the trial. Additionally, the material may include a report *from* the DMC, requests *from* the PMG or draft publications. No trial outcome measure data will be presented by arm unless explicitly authorised by the DMC. Where relevant, accrual, compliance with follow-up and adherence to treatment may be presented by centre. The TSC will receive the report at least 1 week but preferably 2 weeks before any meetings. Different procedures may apply to teleconference meetings.

TSC members are expected to store securely copies of the reports to and from the TSC, agenda and minutes, as well as copies of communications between meetings. All documentation should be considered confidential.

5.4. Contents of the TSC Report

An outline example of the contents of the TSC report is given below:

- Major protocol amendments
- Patient screening
- Eligibility violations
- Protocol deviations/violations by investigators or participants
- Study accrual by month/total
- Completeness and quality of data collected/eCRF return
- Quality controls
- Safety reporting
- Any matters affecting the trial
- Compliance by patients to clinic visit
- Latest DMC recommendations

6. Decision making

All potential independent TSC members will have the opportunity to comment on the protocol as early as possible. Before recruitment begins the trial will have undergone review by the Sponsor/Funder (e.g. peer review for public sector trials), scrutiny by other trial committees and a research ethics committee. Therefore, if a potential independent TSC member has major reservations about the trial (e.g. the protocol, the logistics, ethical concerns) they should report these to the Chief Investigator and may decide not to accept the invitation to join. TSC members will be constructively critical of the ongoing trial, but also supportive of aims and methods of the trial.

6.1. Recommendations

On consideration of the information presented at these meetings, the TSC should provide recommendations of appropriate action in writing to the PMG who will be responsible for implementing any actions. The role of the Chair is to summarise discussions and encourage consensus. Therefore it is best for the chair to give his/ her own opinion last. It is important that the implications (ethical, statistical, practical and financial) for the trial be considered before any decision is made. Decisions and recommendations by the TSC should be unanimous, if not a vote may be taken.

The TSC should inform the PMG if:

- There are concerns about the safety of participants, recruitment, the integrity of trial data or adherence to the protocol.
- The TSC may also provide feedback to the DMC and where appropriate to the Sponsor/Funder.

The DMC may make recommendations to the TSC based on the interim data. Identification and circulation of published external evidence (e.g. from other trials/ systematic reviews) is not the responsibility of the TSC members; it is a responsibility of the PMG. However, the TSC should continue to be made aware of other data that may impact on the trial.

The TSC is the oversight body for the trial. However, the TSC should have good reason(s) before deciding not to accept requests from the PMG and recommendations from the DMC. If there are serious problems or concerns with the TSC decision following a DMC recommendation, a joint meeting of the TSC and DMC should be held. The information to be shown would depend upon the action proposed and each committee’s concerns. Depending on the reason for the disagreement confidential data may have to be revealed to all or some of those attending such a meeting: this would be minimised where possible. The meeting will ideally be Chaired by an external expert who is not directly involved with the trial.

6.2. TSC records

The Minutes of the meeting including key points and actions will be prepared by the facilitator. The Facilitator will keep a central record of all minutes, reports and correspondence by the TSC in the Trial Master File. These minutes will describe the proceedings and include the recommendations of the TSC. All members of the TSC must agree the minutes and these will be signed off by the TSC Chair on behalf of all members. Minutes will be circulated to all TSC members, the PMG, the Sponsor and, if appropriate, the Trial Funder. Approved Minutes will also be filed in the Trial Master File.

7. Trial closure

The TSC, based on recommendations from the DMC, may recommend early termination of the trial or modification of the study design in the event of a clear accumulating data or on the basis of information available from other sources or on safety grounds. Possible decisions include:-

1. No action needed, trial continues as planned.

2. Early stopping due, for example, to overwhelming evidence benefit or evidence of harm of a treatment or external evidence (this should generally involve a recommendation from the DMC to unblind the TSC to these data).
3. Modifying target recruitment, or pre-analysis follow-up, based on any change to the assumptions underlying the original trial sample size calculation (but not on any emerging differences).
4. Sanctioning and/or proposing protocol changes.

9. Publication

Manuscripts that arise from the trial will be shared with the TSC and members will be able to comment. The TSC members and their affiliations will be acknowledged in reports of the trial.

10. References

The charter was developed using:

1. MRC Clinical Trials Unit template TSC Charter version 1.02, 13-Mar-2006
2. CCTU/GD018 Version No.1 Approved: 21/03/2013 http://www.cuh.org.uk/sites/default/files/research/CCTU_GD018%20Trial%20Steering%20Committee%20or%20Study%20Steering%20Committee%20Guidance.pdf accessed 24/04/2014
3. MRC Guidelines for GCP in clinical trials (1998) issued 01/Sep/2006 http://www.mrc.ac.uk/Utilities/Documentrecord/index.htm?d=MRC002416 accessed 24/04/2014
4. MHRA GCP Guide (2012)
5. Conroy et al.: Trial steering committees for randomised controlled trials: updating and redeveloping guidance and terms of reference informed by current practice and experience. Trials 2013 14 (Suppl 1):P128.

Table 1: Potential competing interests for independent members

- Stock ownership in any commercial companies involved
- Stock transaction in any commercial company involved (if previously holding stock)
- Consulting arrangements with the Sponsor/Funder
- Frequent speaking engagements on behalf of the intervention
- Career tied up in a product or technique assessed by trial
- Hands-on participation in the trial
- Involvement in the running of the trial
- Emotional involvement in the trial
- Intellectual conflict e.g. strong prior belief in the trial's experimental arm
- Involvement in regulatory issues relevant to the trial procedures
- Involvement in the writing up of the main trial results in the form of authorship

Table 2: Potential competing interests for non-independent members

- Stock ownership in any commercial companies involved
- Stock transaction in any commercial company involved (if previously holding stock)
- Consulting arrangements with the Sponsor/Funder
- Frequent speaking engagements on behalf of the intervention
- Intellectual conflict e.g. strong prior belief in the trial's experimental arm
