# Supplementary material for: Open randomised trial of the (Arabin) pessary to prevent preterm birth in twin pregnancy with health economics and acceptability: STOPPIT-2—a study protocol
Source: BMJ Open. 2018 Dec 6;8(12):e026430. doi: 10.1136/bmjopen-2018-026430 (PMC6286620; doi:10.1136/bmjopen-2018-026430)
Supplement: Supplementary file 3 [file bmjopen-2018-026430supp003.pdf]

STOPPIT-2

Data Monitoring Committee Charter

**Study Title:**	An open randomised trial of the Arabin pessary to prevent preterm birth in twin pregnancy, with health economics and acceptability – STOPPIT-2.
**Chief Investigator:**	**Professor Jane Norman**
**REC number:**	**14/SS/031**
**Sponsor:**	University of Edinburgh & NHS Lothian

Approval Signatures:

The following individuals, by providing their signatures, indicate their understanding of and willingness to comply with the roles and responsibilities assigned to them in this Charter.

1. DMC Chair:

________________	__________________	_ _/ _ _/_ _ _ _
PRINT NAME	SIGNATURE	DATE

2. DMC Member:

________________	__________________	_ _/ _ _/_ _ _ _
PRINT NAME	SIGNATURE	DATE

3. DMC Member:

________________	__________________	_ _/ _ _/_ _ _ _
PRINT NAME	SIGNATURE	DATE

4. Chief Investigator:

________________	__________________	_ _/ _ _/_ _ _ _
PRINT NAME	SIGNATURE	DATE

5. [Trial/Unblinded] Statistician/Analyst:

________________	__________________	_ _/ _ _/_ _ _ _
PRINT NAME	SIGNATURE	DATE

Table of Contents

1. Introduction 4
2. Primary responsibilities of the DMC 4
3. Membership of the DMC 4
   3.1 Members 4
   3.2 Conflicts of interest 5
4. Timing and purpose of the DMC meetings 5
   4.1 Organisational meeting and early safety/trial integrity reviews 5
   4.2 DMC meetings 6
5. Procedures to ensure confidentiality & proper communication 6
   5.1 Closed sessions 6
   5.2 Open sessions 7
   5.3 Open and Closed reports 7
   5.4 Minutes of the DMC meeting 7
   5.5 Recommendations to the Trial Steering Committee 8
6. Statistical monitoring guidelines 8
7. Content of the DMC's open and closed reports 9
   7.1 Open statistical report: outline 9
   7.2 Closed statistical report: outline 9
8. DMC Payments 10
9. References 10

1. Introduction

This Charter is for the Data Monitoring Committee (DMC) for the STOPPIT-2 An open randomised trial of the Arabin pessary to prevent preterm birth in twin pregnancy, with health economics and acceptability. This study aims to determine whether the Arabin cervical pessary prevents preterm birth in women with a twin pregnancy and a short cervix. Women with a twin pregnancy are at high risk of preterm labour. The extra risk of preterm labour in twin pregnancy is the main reason why twin babies are five times more likely to die, both within the first month or the first year of life. Preterm babies who survive are at increased risk of long term disability, and often require extensive and costly health care, so if preterm birth could be prevented in twins this would be extremely beneficial.

The Charter will define the primary responsibilities of the DMC, its membership, and the purpose and timing of its meetings. The Charter will also provide the procedures for ensuring confidentiality and proper communication, the statistical monitoring guidelines to be implemented by the DMC, and an outline of the content of the Open and Closed Reports that will be provided to the DMC.

2. Primary responsibilities of the DMC

The DMC will be responsible for safeguarding the interests of trial participants, assessing the safety of the interventions during the trial, reviewing external evidence with an impact on risk/benefit balance and for monitoring the overall conduct of the clinical trial. The DMC will provide recommendations about safety, modifying or continuing the trial to the Trial Steering Committee (TSC). To contribute to enhancing the integrity of the trial, the DMC may also formulate recommendations relating to the selection, recruitment, or retention of participants, or their management, or to improving their adherence to protocol-specified regimens and retention of participants, and the procedures for data management and quality control.

The DMC will be advisory to the TSC. The TSC will be responsible for promptly reviewing the DMC recommendations, to decide whether to continue or terminate the trial, and to determine whether amendments to the protocol or changes in study conduct are required.

3. Membership of the DMC

3.1 Members

The DMC is an independent multidisciplinary group consisting of clinicians, statisticians and methodologists that, collectively, have experience/expertise in the management of patients with the condition relevant to study and anticipated adverse effects and in the conduct and monitoring of

randomised clinical trials. University of Edinburgh insurance indemnifies DMC members for their work on the committee.

DMC: Chair (Independent statistician)

DMC: Independent Clinical Investigator (Obstetrician)

DMC: Independent Clinical Investigator (Neonatologist)

DMC: (non-Independent) statistician/analyst (TBC)

(Note – names removed in this version to be published)

3.2 Conflicts of interest

Independent DMC membership is restricted to individuals free of relevant significant conflicts of interest. The source of these conflicts may be financial, scientific or regulatory in nature. Individuals who fulfil any of the following criteria are automatically disqualified from membership: the study investigators, individuals with conflicts of interest as determined by the Chair of the DMC.

The DMC members will disclose to fellow members any consulting agreements or financial interests they have with Edinburgh University, NHS or the manufacturing license holder of an investigational medicinal product/medical device subject to investigation during the study. The DMC will be responsible for deciding whether these consulting agreements or financial interests materially impact their objectivity.

The DMC members will be responsible for advising fellow members of any changes in these consulting agreements and financial interests that occur during the course of the trial. Any DMC member who thinks they may have developed a relevant significant conflict of interest during the course of the trial must declare this. If the other DMC members consider it to be a relevant significant conflict of interest, the member should resign from the DMC.

DMC membership is normally for the duration of the clinical trial. If any member leaves the DMC during the course of the trial, the sponsor, in consultation with the TSC and/or investigators will promptly appoint their replacement.

4. Timing and purpose of the DMC meetings

4.1 Organisational meeting and early safety/trial integrity reviews

This charter was prepared early in the course of the trial (January 2015) by the trial sponsors, trial chief investigator, trial statistician, to be approved by the DMC at their first meeting. At the first meeting the DMC will provide an advisory review of scientific and ethical issues relating to study design and conduct, discuss the Charter for the role and functioning of the DMC, and the format

and content of the Open and Closed Reports that will be used to present trial results at future DMC meetings.

4.2 DMC meetings

The 1st meeting of the DMC has been scheduled before recruitment of the first participant, to discuss the protocol, the charter and the remit of the DMC, and to have the opportunity to clarify any aspects with the principal investigators, including the format of DMC reports from the Study Data Centre at the CHaRT Clinical Trials Unit.

Future meetings will be held at times to be determined by the DMC e.g. after a certain number of patients have been recruited, generally by teleconference, but face-to-face if necessary. The date of each meeting will be made available to the [unblinded/trial] statistician/analyst. The trial chief investigator and trial statistician/analyst will be available for an open session at the beginning of the meeting, and will be available at the end of the meeting to answer any urgent questions.

5. Procedures to ensure confidentiality & proper communication

To enhance the integrity and credibility of the trial, procedures will be implemented to ensure the DMC has sole access to emerging information from the clinical trial regarding safety data, aggregated by treatment arm. [An exception will be made to permit access to an unblinded statistician who will be responsible for creating the closed report and sending it to the DMC.] The sponsors will provide the chair of the DMC with information on any serious unexpected adverse device reactions to the study treatment, and will also be responsible for satisfying the standard requirements for reporting of relevant events to the appropriate authorities.

At the same time, procedures will be implemented to ensure proper communication is achieved between the DMC and the trial investigators and sponsor. To provide a forum for exchange of information among various parties that share responsibility for the successful conduct of the trial, a format for Open Sessions and Closed Sessions will be implemented. The intent of this format is to enable the DMC to preserve confidentiality of the unblinded results while at the same time providing opportunities for interaction between the DMC and others who have valuable insights into trial-related issues.

All materials, discussions and proceedings of the DMC are completely confidential.

5.1 Closed sessions

Sessions involving only DMC membership (but often including the unblinded statistician as well, as a non-voting member) called Closed Sessions will be held to allow discussion of confidential data from the clinical trial, including information about the safety of interventions. In order to ensure that the DMC will be fully informed in its primary mission of safeguarding the interest of participants, the DMC will be unblinded in its assessment of safety data. During these sessions, the DMC will

develop a consensus on its list of recommendations, including that relating to whether the trial should continue.

DMC members and all other participants in the closed section of DMC meetings and the production of unblinded reports are expected to maintain confidentiality, and will refrain from revealing to the TSC, or any other party, information that would lead to compromising the integrity of the trial unless such release is required to protect patient safety.

5.2 Open sessions

In order to allow the DMC to have adequate access to information provided by the trial investigators, or by members of the regulatory authorities, a joint session between these individuals and DMC members (called an Open Session) will be held before the Closed Session. If necessary, a further Open Session can be held, on request either in the middle or end of the Closed Session. Open sessions give the DMC an opportunity to query these individuals about issues that have arisen during their review in the initial Closed Session. With this format, important interactions are facilitated through which problems affecting trial integrity can be identified and resolved. These individuals will either be present at the DMC meeting or be provided a telephone link.

5.3 Open and Closed reports

For each DMC meeting, Open and Closed Reports will be provided (see Section 7 for outlines of the content of these reports). Open Reports, available to all who attend the DMC meeting, will include data on recruitment and pooled data on baseline characteristics, eligibility violations, completeness of follow-up and compliance. The trial manager and the trial statistician/analyst will supervise preparation of these Open Reports.

Closed Reports, available only to those attending the Closed Sessions of the DMC meeting, will include the same information plus adverse events by allocation. The trial statistician/analyst will produce the Closed Reports to the DMC.

The Open and Closed Reports should provide information that is as accurate as possible at the time of preparation, with follow-up that is as complete as possible to within approximately one month of the date of the DMC meeting. The Reports should be provided to DMC members at least one week prior to the date of the meeting.

5.4 Minutes of the DMC meeting

The [DMC Chair/other designated DMC member] will prepare minutes of the DMC meetings. Two sets will be prepared: The Open Minutes and the Closed Minutes.

The Open Minutes will describe the proceedings in the Open Session of the DMC meeting, and will summarize all recommendations by the DMC. Since these minutes will be circulated to the trial co-

chief investigators, it is essential that these minutes do not unblind any results, if the DMC is not recommending early termination.

The Closed Minutes will describe the proceedings from all sessions of the DMC meeting, including the listing of recommendations by the Committee. Because it is likely that these minutes will contain unblinded information, it is important that they are not made available to anyone outside the DMC. Rather, copies will be kept by [the DMC chair/other designated DMC member]. These will be sent to the trial manager and archived at the time of study closure.

5.5 Recommendations to the Trial Steering Committee

At each meeting of the DMC during the conduct of the trial, the DMC will make a recommendation to the TSC. Possible recommendations include:

- Trial continues as per protocol
- Early termination of the trial (or a recommendation for the TSC to review unblinded data), if significant safety issues arise
- Extending recruitment or extending follow-up

The recommendation(s) will be based primarily on safety considerations and will be guided by statistical monitoring guidelines defined in this Charter. Should the DMC decide to recommend early termination of the trial, a full vote of the independent DMC members will be required. All members of the DMC will be required for this to be quorate. In the event of a split vote, the decision will go with the majority vote, but a report should be provided to the TSC, detailing the split. This report should not include unblinded data unless deemed necessary by the DMC. This information should be forwarded to the trial chief investigator as rapidly as possible.

The TSC is jointly responsible with the DMC for safeguarding the interests of participants and for the conduct of the trial. Recommendations to amend the protocol or conduct of the study made by the DMC will be considered and either accepted or rejected by the TSC. The TSC will be responsible for deciding whether to continue or to stop the trial based on the DMC recommendations. The DMC will be notified of all changes to the protocol or to study conduct. The DMC concurrence will be sought on all substantive recommendations or changes to the protocol or study conduct prior to their implementation.

6. Statistical monitoring guidelines

During the period of recruitment into the study, information that is available on safety outcomes such as serious adverse device events believed to be related to treatment will be supplied, in strictest confidence, to all members of the DMC, along with any other analyses that the DMC may request.

7. Content of the DMC's open and closed reports

7.1 Open statistical report outline:

i. Report Information (milestones/gannt chart, date of data lock etc.)

ii. Protocol amendments and update on study documentation

iii. Site/central monitoring report

iv. Adherence to protocol (list of protocol deviations/violations)

v. Recruitment update/uptake rate

vi. Completeness of data collected (reporting missing data 'forms' overall and by site)

vii. Quality of data collected (reporting no. of queries/worst 'offenders' likewise)

viii. Safety reporting - list of Serious Adverse Events/SUSARs

ix. Withdrawals (i.e. adherence to intervention & retention)

   a. from intervention (plus reasons)

   b. from trial data collection i.e. losses to follow-up (plus reasons)

x. Other issues

xi. Recent relevant publications

7.2 Closed statistical report outline:

i. Report Information (date of data point/lock)

ii. Baseline comparability (for minimisation factors by group)

iii. Interim results for primary outcome(s) and selected secondary outcomes (either safety or process)

iv. Safety reporting – Unexpected Serious Adverse Device events/USADE (by group)

v. Safety reporting - list of Serious Adverse Events/SUSARs (to review event rates)

vi. Withdrawals (adherence to intervention & retention) (by group)

   i. from intervention (plus reasons)

   ii. trial data collection & follow-up (plus reasons)

8. DMC Payments

DMC members will be reimbursed for travel and accommodation. No other payments or rewards are anticipated but their contribution will be acknowledged where relevant in publications.

9. References

1. DAMOCLES Study Group. A proposed charter for clinical trial data monitoring committees: helping them to do their job well. The Lancet 2005; vol 365: 711-722.
