# Supplementary material for: Open randomised trial of the (Arabin) pessary to prevent preterm birth in twin pregnancy with health economics and acceptability: STOPPIT-2—a study protocol
Source: BMJ Open. 2018 Dec 6;8(12):e026430. doi: 10.1136/bmjopen-2018-026430 (PMC6286620; doi:10.1136/bmjopen-2018-026430)
Supplement: Supplementary file 4 [file bmjopen-2018-026430supp004.pdf]

STOPPIT 2 Publication guidelines

All relevant contributors will be acknowledged (by name where possible) in ensuing publications. Authorship will be assigned according to journal or ICMJE guidelines (see http://www.icmje.org/).

Briefly, ICMJE suggest that authorship credit should be based on: 1) substantial contributions to conception and design, acquisition of data, or analysis and interpretation of data; 2) drafting the article or revising it critically for important intellectual content; and 3) final approval of the version to be published. Authors should meet conditions 1, 2, and 3.

According to these guidelines therefore the sole activity of recruitment of participants or supervision of recruitment of participants, whilst crucially important, will qualify for contributorship, but is unlikely to qualify for authorship. The original grant applicants will qualify for authorship, as long as they continue to be involved in the study, and can also therefore satisfy conditions 2 and 3 above.

Final decisions on publication and authorship will be made by the trial steering committee.
