# Supplementary material for: Open randomised trial of the (Arabin) pessary to prevent preterm birth in twin pregnancy with health economics and acceptability: STOPPIT-2—a study protocol
Source: BMJ Open. 2018 Dec 6;8(12):e026430. doi: 10.1136/bmjopen-2018-026430 (PMC6286620; doi:10.1136/bmjopen-2018-026430)
Supplement: Supplementary file 5 [file bmjopen-2018-026430supp005.pdf]

Appendix 5 **PROTOCOL REVISIONS**

PROTOCOL VERSION	DATE	REASON FOR UPDATE	SUBSTANTIAL AMENDMENT NUMBER	SUMMARY OF CHANGES
**2**	02nd June 2015	Text reformatted and clarified, information updated Questionnaires created and updated	**1**	**General updates/reformatting**<br>Clarification of secondary endpoints (obstetric/neonatal)<br>**Additional clinical information to be collected, to allow sensitivity analyses in future individual patient data meta-analyses**<br>Internal pilot phase interviews refined and made specific<br>Inclusion/exclusion criteria updated<br>Clarification of withdrawal<br>Pessary criteria updated<br>Ultrasound accreditation method updated<br>Qualitative interviews/questionnaires and Health Economics questionnaires included and information updated<br>PAG information updated<br>Study monitoring procedures updated<br>Trial Steering Committee details updated<br>Data Monitoring Committee details updated<br>Secondary end point definitions added (Appendix 5)
**3**	5th August 2015	Change of measurement which defines “short cervix” and confers eligibility for randomisation	**2**	Change of measurement which defines a short cervix from ≤ 30mm to ≤ 35mm. The anticipated population centile (≤ 30th centile) is unchanged.
**4**	10th January 2018	Clarification of text, modification of the data collection and safety	**5**	• Clarification of Co-enrolment<br>• Modification of data collection to include hospitals, which are not participating STOPPIT2 sites<br>• Safety reporting guidance modified for clarity.<br>• Increase in the number of participants from 1850 to 2500 for cervical length scanning to facilitate randomisation which remains at 500.
