# Supplementary material for: Open randomised trial of the (Arabin) pessary to prevent preterm birth in twin pregnancy with health economics and acceptability: STOPPIT-2—a study protocol
Source: BMJ Open. 2018 Dec 6;8(12):e026430. doi: 10.1136/bmjopen-2018-026430 (PMC6286620; doi:10.1136/bmjopen-2018-026430)
Supplement: Author's manuscript [file bmjopen-2018-026430.draft_revisions.pdf]

BMJ Open is committed to open peer review. As part of this commitment we make the peer review history of every article we publish publicly available.

When an article is published we post the peer reviewers' comments and the authors' responses online. We also post the versions of the paper that were used during peer review. These are the versions that the peer review comments apply to.

The versions of the paper that follow are the versions that were submitted during the peer review process. They are not the versions of record or the final published versions. They should not be cited or distributed as the published version of this manuscript.

BMJ Open is an open access journal and the full, final, typeset and author-corrected version of record of the manuscript is available on our site with no access controls, subscription charges or pay-per-view fees (http://bmjopen.bmj.com).

If you have any questions on BMJ Open's open peer review process please email editorial.bmjopen@bmj.com

BMJ Open

An open randomised trial of the (Arabin) pessary to prevent preterm birth in twin pregnancy, with health economics and acceptability – STOPPIT-2.

Journal:	*BMJ Open*
Manuscript ID	bmjopen-2018-026430
Article Type:	Protocol
Date Submitted by the Author:	14-Sep-2018
Complete List of Authors:	Norman, Jane; University of Edinburgh<br>Norrie, John; Edinburgh Clinical Trials Unit, University of Edinburgh No. 9, Bioquarter<br>Maclennan, Graeme; University of Aberdeen, Health Services Research Unit<br>Cooper, David; University of Aberdeen, Health Services Research Unit<br>Whyte, Sonia; The University of Edinburgh<br>CunninghamBurley, Sarah; University of Edinbrugh, Public Health Sciences<br>Smith, Joel; University of Oxford, Health Economics Research Centre, Nuffield Department of Population Health<br>Shennan, Andrew; Kings College London, Maternal and Fetal Research Unit<br>Robson, Stephen; NIHR CRN North East and North Cumbria<br>Thornton, Steven; Queen Mary University of London<br>Kilby, Mark; Birmingham Women's and Children's NHS Foundation Trust<br>Marlow, Neil; University College London, Institute of Child Health<br>Stock, Sarah; University of Edinburgh MRC Centre for Reproductive Health, Tommy's Centre for Maternal and Fetal Health; University of Western Australia School of Women's and Infant's Health,<br>Bennett, Philip; Imperial College London Department of Surgery and Cancer<br>Denton, Jane; Bryan Multiple Births Centre, Director of the Multiple Births Foundation
Keywords:	preterm birth, cervical pessary, twin pregnancy, preterm labour

SCHOLARONE™
Manuscripts

Title: An open randomised trial of the (Arabin) pessary to prevent preterm birth in twin pregnancy, with health economics and acceptability – STOPPIT-2.

Authors: Norman JE[1], Norrie J[2], MacLennan G[3], Cooper D[3], Whyte S[1], Cunningham Burley SC[4], Smith JBE[5], Shennan A[6], Robson SC[7], Thornton S[8], Kilby MD[9], Marlow N[10], Stock S[4], Bennett PR[11], Denton J[12]

Affiliations

1. Tommy's Centre for Maternal and Fetal Health, MRC Centre for Maternal and Fetal Health, University of Edinburgh, Edinburgh, UK
2. Edinburgh Clinical Trials Unit, University of Edinburgh, Edinburgh, UK
3. Centre for Healthcare Randomised Trials, University of Aberdeen, Aberdeen, UK
4. Usher Institute, University of Edinburgh, Edinburgh, UK
5. Health Economics Research Centre (HERC), Nuffield Department of Population Health, University of Oxford, Oxford, UK
6. Tommy's Centre for Maternal and Fetal Health, King's College London, London, UK
7. Institute of Cellular Medicine, University of Newcastle, Newcastle, UK
8. Barts and the London School of Medicine and Dentistry, Queen Mary University of London, London, UK
9. Fetal Medicine Centre, Birmingham Women's & Children's Foundation Trust and College of Medical and Dental Science, University of Birmingham, Birmingham, UK
10. Institute for Women's Health, University College London, London, UK
11. Imperial College London, London, UK
12. Multiple Births Foundation, London, UK

Corresponding author:
Professor Jane E Norman
Tommy's Centre for Maternal and Fetal Health,
MRC Centre for Maternal and Fetal Health
University of Edinburgh
The Queen's Medical Research Institute
47 Little France Crescent
Edinburgh
EH16 4TJ
Tel: +44 (0) 131 242 2694
Email: jane.norman@ed.ac.uk

Keywords: preterm birth, cervical pessary, twin pregnancy, preterm labour

Word count (excluding title page, abstract, references, figures, tables): 6010

For peer review only

Abstract:

Introduction

The STOPPIT-2 study aims to determine the clinical utility of the Arabin cervical in preventing preterm birth in women with a twin pregnancy and a short cervix, about which there is current uncertainty[1 2 3]. STOPPIT-2 will resolve uncertainty around effectiveness for women with a twin pregnancy and a cervical length of 35mm or less, define adverse effects, ascertain acceptability and estimate NHS costs and savings.

Methods

STOPPIT-2 is a pragmatic multicentre open label randomised controlled trial. Consenting women with twin pregnancy will have an transvaginal ultrasound scan of their cervical length performed between 18+0 to 20+6 weeks gestation by an accredited practitioner: women with a cervical length of ≤ 35mm will be eligible for inclusion in the treatment phase of the study. The intervention by the insertion of the Arabin cervical pessary will be compared with standard treatment (no treatment).

The primary outcomes are (obstetric) spontaneous onset of labour for the mother leading to delivery before 34 weeks gestation and (neonatal) a composite of specific adverse outcomes or death occurring up to the end of the first four weeks after the estimated date of delivery to either or both babies.

We plan to recruit 500 women in the treatment phase of the study. Assuming a treatment effect of 0.6, and background rates of 35% and 18% respectively for each of the primary outcomes, our study has 85% power to detect a difference between the intervention and the control groups.

Analysis

Data will be analysed on the intention to treat principle.

Ethics

STOPPIT-2 was approved by the South East Scotland Ethics Committee 02 on 29th August 2014, reference number 14/SS/1031 IRAS ID 159610.

Dissemination

Peer reviewed journals, presentations at national and international scientific meetings.

Registration.

ISRCTN and NCT websites on 3/12/14 and 9/9/14, ref nos ISRCTN98835694, NCT02235181.

For peer review only

Strengths and limitations of the study

Strengths

- Randomised multicentre trial
- Cervical length measured by accredited individuals
- Prespecified cervical length threshold for randomisation
- Larger sample size than previously studied

Limitations

- Inability to blind women or caregivers to treatment allocation

Introduction

Preterm birth is common in twin pregnancy. The rate of twinnng itself is increasing. Twinning is associated with significant death or serious morbidity for the babies but there are no effective treatments. In 2010, 52.7% of multiple births were preterm and there is a clear health need for prevention of preterm birth in twins. This excess of prematurity (compared with singletons) leads to neonatal and infant mortality rates of 14.2 and 18.8 per 1,000 live births respectively (over fivefold that of singletons). Progesterone appears ineffective in preventing preterm birth in twins [4] and cerclage appears harmful [5]. Prematurity is thought to account for over 70% of twin neonatal deaths and adversely affects fetal survivors, with increased risks of future respiratory problems, motor and sensory impairment, learning difficulties, and social and behavioural difficulties.

The costs to the NHS of prematurity in twins are considerable. Twins alone account for over 20% of neonatal unit cot stays, a significant excess given they comprise only 2% of all births. Together, the complications of preterm birth result in an estimated annual cost of £2.946 billion to the public purse in England and Wales (2006 prices) [6]. Twins make a major contribution to this. There is a clear expressed need for innovative interventions to reduce preterm birth in both high and low income countries [7, 8]. The 2011 NICE Multiple Pregnancy Guideline Group noted that bed rest at home or in hospital, progesterone, cervical cerclage and oral tocolytics are all ineffective at preventing preterm birth in twins, concluding that alternative effective interventions are urgently required. The guideline predated the publication of studies on pessary to prevent preterm birth in twins, and there was no attempt to evaluate effectiveness. The Cochrane review on “cervical pessary for preventing preterm birth” [9] identified only one randomised trial (in singletons) which “showed the beneficial effect of the cervical pessary in preventing preterm birth”, and called for randomised trials to be performed in multiple pregnancy [9].

Four randomised trials have been reported efficacy of cervical pessaries to prevent preterm birth in twins. A summary forest plot on the efficacy of the pessary to prevent preterm birth (using the entire population randomised each study) is shown in the Figure. The first trial to be published was the ProTwin study, which randomised 813 women with twin pregnancy to treatment with an Arabin pessary (inserted between 16 and 20 weeks gestation) or to standard treatment[10]. Although the pessary did not reduce preterm birth or the primary neonatal composite outcome overall, both of these events were significantly reduced in the prespecified subgroup of women with a cervical length less than the 25th centile (n=143) with a RR (95% CI) of preterm birth before 32 weeks of 0.49 (0.24-0.97) and RR (95%) of composite neonatal outcome of 0.42 (0.19 - 0.91). A subsequent randomised study of1,180 unselected women showed no difference in the rate of the primary outcome of spontaneous birth <34 weeks (13.6% vs. 12.9%; relative risk 1.054, 95% confidence interval [CI] 0.787-1.413; p=0.722) [1]. A preplanned subgroup analysis of 214 women with a cervical length of ≤ 25 mm also showed no benefit of the pessary (hazard ratio, 1.256; 95% CI, 0.760- 2.074; P =0.374). These data contrast with a smaller study published the same year, showing that, in women with a cervical length of ≤ 25 mm, spontaneous preterm birth was less common in the pessary group 11/68 (16..2%) compared with the expectant management group (26/66 (39.4%)[11]. Lastly, the POPT study of women with twin pregnancy and a cervical length </= 30 mm at 18 + 0 to 27 + 6 weeks' gestation randomised 46 women and found no difference in the primary outcome of preterm birth < 34 weeks (39% vs 35%; relative risk, 1.13 (95% CI, 0.53-2.40)[3].

Taken together, these data suggests that the cervical pessary does not work in unselected women, but may work in women with a short cervix; (at higher risk of preterm birth); consequently STOPPIT-2 will test the efficacy of the Arabin pessary in women with a short cervix. A cervical length threshold for recruitment of less than or equal to the 30th centile was chosen to maximise the number of women

For peer review only - http://bmjopen.bmj.com/site/about/guidelines.xhtml

who could benefit. When planning the trial, it was thought that the 30th centile for cervical length would equate to a cervix of less than or equal to 30mm in length, based on a large UK study [12]. However, a meta-analysis published in the early phases of the pilot for STOPPIT-2 [13], and data from the first 20 women in STOPPIT-2 itself, suggested that the 30th centile of cervical length in twins at 18-21 weeks gestation is 35mm. A cervical length threshold of less than or equal to 35mm (which we assume to be the 30th centile) was therefore used for eligibility. The quality control measures of the Fetal Medicine Foundation (http://www.fetalmedicine.com), or the Perinatal Quality Foundation (https://clear.perinatalquality.org), are employed to ensure validity of cervical length measurements.

Research priorities identified by NICE include randomised trials to determine "[effective interventions] in preventing spontaneous preterm birth in women with twin[s] .., especially in those at high risk of preterm birth". Additionally, the Preterm Birth Clinical Study Group of the RCOG identified "Efficacy of the Arabin pessary" amongst its top five research priorities. We are aware of several other studies in active or planned recruitment comparing a cervical pessary with standard care for prevention of preterm birth or its consequences in women with a preterm birth including NCT03418311 (planned n=672), NCT03058536 (planned n=312), ACTRN12616000875404 (planned n=140), NCT02708264 (planned n=242), NCT02350231 (planned n=100), and NCT01334489 (planned n=352).

After publication of STOPPIT-2 results, we plan to collaborate in subsequent individual patient data meta-analyses and update the economic decision model in light of new evidence. If the cervical pessary is effective in reducing preterm birth in twin pregnancy, it could make a major contribution to human health and ensure a more efficient allocation of resources in the UK NHS. However, we need to determine whether it is effective (and safe), since the evidence to date is not strong or robust enough to support its adoption as a first line intervention.

Methods

Design

STOPPIT-2 is a multicentre open label randomised controlled trial of the Arabin pessary (CE marked Device) versus standard treatment in women with twin pregnancy recruited from NHS antenatal clinics. The study is in two phases – a SCREENING phase, in which women with a short cervix (cervical length of ≤ 35mm) are identified, and a TREATMENT phase, in to which women with a short cervix will be randomised to either treatment with Arabin pessary or Standard treatment. An internal pilot will be performed to assess recruitment rates and to determine feasibility.

Objectives

Primary Objective

The primary objective of this study is to test the hypothesis that the Arabin cervical pessary reduces spontaneous preterm birth in women with a twin pregnancy and a short cervix (less than or equal to 35mm), reducing adverse neonatal outcomes and health care costs. We will also address the hypothesis that the pessary is acceptable to pregnant women. Subgroup analyses will determine effectiveness in women with a cervical length less than or equal to 25mm, and in women with a dichorionic pregnancy.

Secondary Objectives

The secondary objectives are to confirm (using the entire cohort of women undergoing cervical length scanning) the profile of cervical length measurements in women with twin pregnancy in the UK, and the positive and negative likelihood ratios of a variety of cervical length thresholds to determine spontaneous preterm birth before 34 weeks gestation.

Randomisation

Randomisation to Arabin pessary or standard treatment is performed by the central randomisation facility at the study data centre using a web portal. The allocation sequence will be generated by computer. Participants will be assigned a unique study identifier and staff will be required to enter minimal patient details prior to randomisation. Randomisation will be minimised with a random element rather than stratified given the properties of minimisation in ensuring comparability between the groups. Minimisation variables include booking hospital, cervical length and chorionicity (mono or dichorionic) but are not limited to these. The study is open (i.e. not masked), so that both the

For peer review only - http://bmjopen.bmj.com/site/about/guidelines.xhtml

participant and the Investigator will know which treatment has been allocated. The investigator will inform the woman of treatment allocation after randomisation.

Population
We will recruit pregnant women who meet the study eligibility criteria and who are booked for delivery at one of the participating sites. Case notes will be reviewed by the local clinical or research teams prior to recruitment. We anticipate that all eligible women expecting twins and who are attending for antenatal care in each of the sites will be invited to participate. Eligible women will normally be informed of the study at routine antenatal appointments. The following inclusion and exclusion criteria will apply.

Inclusion criteria (all must apply)
- Presenting with twin pregnancy (monochorionic or dichorionic).
- Gestation established by scan at ≤16 weeks according to NICE guidelines.
- Aged 16 years or older
- Wishing to participate in both the SCREENING and RANDOMISATION phase of the study
- Known chorionicity (as defined by first trimester ultrasound screening)

Exclusion criteria – screening phase (none must apply)
- Unable to give written informed consent
- Known significant congenital structural or chromosomal fetal anomaly at the time of inclusion
- Existing or planned cervical cerclage in the current pregnancy
- Existing or planned (prior to 20+6 weeks gestation) treatment for twin to twin transfusion syndrome in the current pregnancy
- Suspected or proven rupture of the fetal membranes at the time of recruitment
- Bulging fetal membranes at the time of recruitment
- Singleton pregnancy or higher order multiple pregnancies and/or women who have had any fetal death (i.e. fetal heart, previously detected) in the index pregnancy (prior to randomisation).
- Known sensitivity, contraindication or intolerance to silicone
- Involved in a clinical trial of an investigational medicinal product (CTIMP), phase 1 study or investigating a treatment for the prevention of pre-term birth
- Monochorionic monoamniotic pregnancy
- Heavy bleeding due to a low lying placenta prior to randomisation

Inclusion criteria – screening phase
- Cervical length ≤ 35mm at 18+0 – 20+6 weeks gestation confirmed by an accredited clinician

Exclusion criteria – treatment phase (none must apply)
- Cervical length > 35mm at 18+0 – 20+6 weeks gestation.
- Cervical length not measured at 18+0 – 20+6 weeks gestation.
- Bulging fetal membranes at the time of pessary insertion.
- Suspected or proven rupture of the fetal membranes at the time of pessary insertion.

To assess eligibility for the TREATMENT phase, all women who fulfil the inclusion criteria for the SCREENING phase will undergo ultrasound measurement of cervical length between 18+0 and 20+6 weeks gestation by an accredited operator (see later). If convenient, cervical length measurement may be done at the time of the routine fetal anomaly scan. In centres where cervical length measurements are part of routine clinical care women may be consented after the cervical length and will not be asked to have a further TV scan, provided:
- The transvaginal scan measurement was taken by an accredited operator (completed CLEAR [USA] or Fetal Medicine Foundation cervical length training) and is delegated by the PI
- The TV scan was conducted between 18+0 and 20+6 weeks gestation,
- The woman is eligible (as per the above inclusion/ exclusion criteria) and the pregnancy is not ≥ 21 weeks gestation.

Images of the cervical length measurements will be anonymised and uploaded to the eCRF.

For peer review only

Women with a cervical length of ≤ 35mm will continue to the TREATMENT phase, following verbal reconfirmation of intent to participate and randomised to either the intervention or the comparator group, described below.

Enrolment and adherence of participating centres
We will recruit from UK NHS and European hospitals. Sufficient centres will be involved to ensure that our planned sample size is achieved. Regular meetings will be held with clinicians and research staff from recruiting centres to maintain study enthusiasm and to achieve our planned sample size.

Intervention
The intervention is the Arabin pessary, inserted through the vagina and around the cervix between 18+0 and 20+6 weeks gestation, and given in addition to standard care. Video instructions for pessary insertion are available on the Arabin website[1]. There are no prohibited co-treatments. A trained member of the local research team will insert the pessary.

Comparator
The comparator is standard care. There are no prohibited co-treatments.

Outcomes (Women recruited to screening and treatment phase)
There are two primary outcomes, an obstetric and a neonatal primary outcome.

- Obstetric primary outcome: all births before 34+0 weeks following the spontaneous onset of labour. Preterm prelabour rupture of membranes < 34 weeks with or without contractions will also be included in this definition of spontaneous onset of labour [14]. Iatrogenic delivery due to maternal or fetal conditions will not be considered to fulfil the criteria for the primary outcome.
- Neonatal primary outcome: the primary neonatal outcome is a composite of adverse outcomes including stillbirth or neonatal death, periventricular leukomalacia, early respiratory morbidity (defined as any need for supplemental oxygen >30%, CPAP [continuous positive airway pressure] or intra-tracheal ventilation or surfactant replacement therapy within the first week of life), intraventricular haemorrhage, necrotising enterocolitis and proven sepsis all measured up to 28 days after the expected date of delivery.

Obstetric
Key outcomes

- Mean gestation at delivery
- Adverse events: infection, cervical trauma
- Incidence of all births before 37+0 weeks gestation
- Acceptability of the pessary as determined by participant questionnaire
- Experience of pessary removal

Other outcomes

- Incidence of all births before each of 28+0, 32+0, and 34+0 weeks gestation
- Preterm birth before 34 weeks gestation preceded by preterm premature membrane rupture
- Incidence of all births before 28, 32, 34 and 37 weeks preceded by the spontaneous onset of labour
- Method of delivery (in three categories: spontaneous vaginal delivery or vaginal breech, forceps or ventouse, caesarean section)
- Duration of labour overall, and of each of the first and second stages of labour
- Duration of stay in hospital
- Other adverse events including haemorrhage, tachycardia, vaginal injury, other trauma
- Serious maternal adverse events up to 28 days after discharge from the hospital

Key Neonatal secondary outcomes:

- Incidence of each of the individual components of the primary neonatal outcome
- Median weight (g) of the newborn at birth
- Death of live born babies within the first 28 days after birth
- Discrete episodes of bloodstream or CNS infection (positive blood or CSF culture)

---
[1] https://dr-arabin.de/produkt/arabin-cerclage-pessary-perforated/?lang=en

For peer review only

- Within first 72 hours
- Between 72 hours and discharge

Other neonatal outcomes

- Birth weight centile (for gestation) within 4 weeks after expected date of delivery
- Death of live born babies within the first 28 days after EDD
- Cord pH
- Apgar score at 1 and 5 minutes
- Need for resuscitation
- Need for surfactant administration
- Bronchopulmonary dysplasia
- Retinopathy of prematurity (stage 1 or more)
- Retinopathy of prematurity requiring surgery (laser or cryotherapy)
- Necrotising enterocolitis, (medical or surgical treatment of confirmed cases
- Twin to twin transfusion (Quintero stage 1 or more)
- Days of respiratory support [either mechanical ventilation or CPAP]
- Days of oxygen therapy
- Daily level of care.
- Seizures requiring therapy.
- Hyperbilirubinaemia requiring exchange transfusion.
- Periventricular leukomalacia / echogenicity

Other outcomes include:

- Acceptability and experience of the pessary as determined by participant questionnaire issued at 36 weeks gestation
- The joint distribution of the cumulative hospital costs and the primary clinical outcome.
- Cervical length measurements for all participants to determine the likelihood ratios for short cervix and spontaneous preterm birth.

We will also collect information on use of the following therapies to allow sensitivity analyses in future individual patient data meta-analyses

- Progestogens (IM or vaginal) after 16 weeks gestation
- Cervical suture post randomisation
- Tocolytics
- Steroids
- Magnesium sulphate
- Antibiotics
- Arabin pessary in the screened only women

Outcomes (women recruited to screening phase only).
For women recruited to the screening phase but are not randomised we will collect maternal and neonatal outcomes as described above. We will not collect information on co-treatments (other than those described above), adverse events, acceptability or costs.

Criteria for discontinuing intervention
Participation is voluntary. A woman has the right to discontinue or withdraw at any time for any reason. The Investigator also has the ability to discontinue a participant. The reason and circumstances will be documented in the electronic case report form. Unless a participant asks to completely withdraw from the study, those who discontinue treatment will be asked to continue to participate in study visits, to facilitate analysis under the intention to treat principle.

If treatment is suspended or stopped due to a serious adverse reaction, the local Principal Investigator will arrange for follow-up visits or telephone calls until the event has resolved or stabilised or until the study has ended, whichever is sooner. In order to avoid bias and/or missing adverse events, outcome data will be collected (including data from the patient record) and used in the analysis.

We will note whether participants have adhered to the treatment assignation. In the intervention arm, we will define adherence as women who have a pessary in situ at 28 weeks gestation (whether or not

For peer review only

it has been out and been reinserted during that time). Women who deliver before 28 weeks, will count as adherent if they had their pessary until 7 days before delivery. Those in the standard care arm will be considered adherent unless they had a pessary inserted.

Visits
Study visits will be conducted at approximately four weekly intervals (plus or minus one week) following randomisation until 36 weeks, when the pessary will be removed. The study visits will be carried out on those randomised to the pessary and those undergoing standard treatment. Study visits could be face to face or by phone or other means of communication, e.g. via letter or email. Pregnancy and fetal well-being will be reviewed at these visits. Additionally, we will collect the following outcomes:

- Any pregnancy complications (adverse events) including bleeding.
- Experience of the pessary during the previous 4 weeks (if applicable)
- Hospital admissions (Serious Adverse Events) – reasons and duration

Pessary removal
The pessary will be removed at 35^{+0} - 36^{+6} weeks gestation. The participant will be asked to complete a short questionnaire about her experience and (if appropriate) of having the pessary. For those allocated to pessary treatment, a vaginal examination will be conducted and the pessary removed. Information will be collected on the ease with which removal was achieved. If the pessary is removed before 35 weeks, we will record the indication.

Ancillary and post trial care
Care of participants in parallel to and beyond the trial will be provided within the UK National Health service. There will be no no-fault compensation for those who suffer harm from trial participation.

Qualitative data
A nested qualitative study involving pregnant women will explore their views and experiences of methods of recruitment and the consent process, their understanding and expectations of trial participation, including randomisation and the screening component, and experiences of the intervention. We will interview women who:

- Consented, were screened but found to have a long cervix (up to 10)
- Consented, were screened, have a short cervix and were randomised and allocated to the control group (up to 10)
- Consented, were screened, have a short cervix and were randomised and allocated to the treatment group (up to 20).

Up to 15 interviews will be conducted with a range of health care professionals involved in the trial in order to explore their views and experiences of the process.

Baseline characteristics (screening and treatment phase)
In addition to the outcomes, we will collect the following baseline characteristics on all women:
Age
Height
Weight
Cervical length
Current Smoking
Current alcohol

Obstetric History: Parity, Miscarriage
Medical conditions: Hypertension, Insulin dependent diabetes, Respiratory disease, Cardiac disease Neurological disease, Skin condition, Thrombophilia, Current Pregnancy
Fetal anomaly scan: Twin 1, Twin 2 (whether performed and the result)
Amniocentesis: Twin 1, Twin 2 (whether performed and the result)

Data collection forms are available on the STOPPIT-2 website at https://w3.abdn.ac.uk/hsru/STOPPIT2/Public/DownloadPage.aspx

For peer review only

Consent
Written informed consent will be obtained from all participating women. Model information sheet and consent forms are available on the study website at https://w3.abdn.ac.uk/hsru/STOPPIT2/Public/DownloadPage.aspx. Women will be given information about the study by the investigator team at each study site, invited to consider participation and will be given adequate time (at least 24 hours) to read the information sheet. If the woman waives this opportunity but still wishes to participate, consent may be taken after a shorter time. Details of the consent process, including the name of the doctor confirming eligibility and the member of the study team taking consent will be recorded in the woman's notes and in the eCRF. Women undergoing ultrasound measurement of the cervix will be asked to complete a consent form prior to the procedure, which will cover both SCREENING and TREATMENT phases of the study. This will minimise inconvenience to the women (reducing the number of visits required to the hospital) and minimise the time undergoing vaginal examination. We will seek permission for future long term follow up of the women and babies via record linkage into national databases (health, social, and educational) although such follow up is beyond the scope of the study described here.

Data Analysis (SCREENING phase data)
We will use cervical length measurements at baseline screening, gestation at delivery, whether delivery was preceded by spontaneous onset of labour and / or preterm premature membrane rupture to determine positive and negative likelihood ratios for spontaneous preterm birth before 34 weeks for a variety of cervical length thresholds. Women treated with the pessary will be excluded from this analysis.

Data Analysis (TREATMENT phase data)
The statistical analysis will be according to the intention to treat principle; all participants will remain in their allocated group for analysis. Statistical significance will be at the 5% level with corresponding 95% confidence intervals (CI) derived. Randomised groups will be described at baseline and follow-up using mean (standard deviation), median (inter-quartile range) and counts (with percentages) where appropriate.

For the primary obstetric and neonatal outcomes, logistic regression with a fixed effect for the minimisation covariate chorionicity and a random effect for centre will be used to obtain the odds ratios of the treatment effect, along with the 95% confidence interval and associated p-value. In the case of the composite neonatal outcome the components will also be reported individually to show which elements are driving the primary outcome. In the case of skewed recruitment resulting in small centres a regional effect will be considered in place of a centre effect.

Continuous secondary outcomes will be analysed using linear regression, adjusting for chorionicity. Binary categorical secondary outcomes will be analysed using logistic regression as per the primary outcomes.

Secondary outcomes with more than two categories will be analysed using multinomial logistic regression.

Analysis of fetal outcomes will allow for clustering within twins by fitting mother as a random effect in a mixed effects logistic regression model.

We will undertake predefined subgroup analyses of the primary outcome by monochorionicity, cervical length (≤ 25mm and ≤ 28mm. In the subgroup analyses, statistical significance will be at the 1% level with corresponding 99% confidence intervals (CI).

It is not anticipated that primary outcome data will be missing. If data are missing, this is likely to be due to a miscarriage, stillbirth, neonatal or maternal death and therefore it will not be imputed.

A post-hoc comparison will also be performed between participants who had membrane rupture and those who did not.

A per protocol analysis (including those in the intervention arm who are adherent, and excluding those in the control arm who have a pessary inserted) will also be performed.

For peer review only

No formal interim analyses are planned.

The statistical analysis plan is included as Appendix 1 to this paper.

Data management

The primary source of data arise from medical notes. These data are entered directly from source, by study site staff, into the eCRF (electronic case report form). The eCRF has been developed in accordance with CHaRT software development standards at the University of Aberdeen. Data entry is carried out remotely by authorised study personnel. Accuracy of entry and the storage of source documentation is the responsibility site study personnel. Validation checks will be set up and run in real time on the eCRF. Checks will include missing data, out of range values, illogical entries and invalid responses. Users will be informed of data issues when the eCRF page is saved providing users with the opportunity to correct the data immediately, where possible. The eCRF system will generate a missing data query for all items not completed at the time. Cross form checks comparing data items across different forms will be created, where possible or written as reports.

Data Analysis (Health economic data)

The economic component will consider the joint distribution of the cumulative hospital costs and the primary clinical outcome from the perspective of the UK NHS.NHS. Generalised linear models will be used to estimate the costs between the two treatment groups. The cost estimates and primary clinical outcome will be combined within a cost-effectiveness analysis to calculate the incremental cost per preterm birth prevented. The length of stay distributions will be valued using a per diem unit cost derived from NHS reference costs[13]. Monetary values will be attached to the labour costs for healthcare practitioners associated with each treatment pathway using standard NHS pay and price estimates[15, 16]. No discounting of hospital costs and clinical outcomes will be undertaken for the within-trial economic component. Flexible parametric regression models will be used to explore the dependency between cost and the primary clinical outcome as well as heterogeneous treatment effects within a sensitivity analysis to inform a decision-analytic model. AA decision model will be developed to estimate the longer term resource consequences and health outcomes of preterm birth following twin pregnancy. Model parameter uncertainty will be addressed using probabilistic sensitivity analysis summarised using the cost-effectiveness acceptability curve at differing willingness to pay thresholds..

Data Analysis (Qualitative research)

The nested qualitative interview study will be analysed using an inductive cross-sectional thematic approach described by Mason[17]. This approach involves applying indexing coding to the entire data set providing the means to identify patterns and themes in the data from each arm of the study. The use of qualitative software will also help to facilitate a collaborative approach to the analysis thereby ensuring consistency and rigour[18]. This method has been chosen as it will help to identify how women experience all aspects of the study in the context of their current pregnancy that builds up a descriptive and conceptual interpretation of the findings that maintains the integrity of each research participant's accounts. The same analytical approach will be taken for the data generated through interviews with health care professionals.

Study monitoring

On site, trial monitoring will not be conducted on behalf of the Co-Sponsors. Remote monitoring to verify eligibility, consent, staff training and data entry quality will be performed. The research involves the use of a CE marked device used for its intended purpose. Audits may be carried out by individual site R&D departments as per the Research Governance Framework, these audits are independent of the sponsors.

A trial steering committee (TSC) and a data monitoring committee (DMC) will be appointed. The membership and charter for each is included in Appendices 2 and 3. The committees will meet not less than annually, or more often if issue arise. There are no formal stopping rules: the TSC and DMC charters state that "The TSC, based on recommendations from the DMC, may recommend early termination of the trial or modification of the study design in the event of a clear accumulating data or on the basis of information available from other sources or on safety grounds". "Recommendations to amend the protocol or conduct of the study made by the DMC will be considered and either accepted

For peer review only

or rejected by the TSC. The TSC will be responsible for deciding whether to continue or to stop the trial based on the DMC recommendations".

Confidentiality of data

All records such as laboratory specimens, questionnaires and evaluation forms, will be identified in a manner designed to maintain participant confidentiality. All records will be kept in a secure storage area with limited access. Collection of participant identifiers is performed only to ensure compliance with governance procedures (eg confirmation of informed consent). Paper records with identifying information will be kept in a locked cabinet with limited access. Electronic identifying information will be kept in a separate trial database and will not be revealed as part of routine data analysis or in any data sharing exercise. Clinical information will not be released without the written permission of the participant. The Investigator and study site staff involved with this study will not disclose or use for any purpose other than performance of the study, any data, record, or other unpublished, confidential information disclosed to those individuals for the purpose of the study. Prior written agreement from the sponsor or its designee must be obtained for the disclosure of any said confidential information to other parties.

Data protection

All Investigators and study site staff involved with this study must comply with the requirements of the Data Protection Act 1998 and subsequent General Data Protection Regulations 2018 with regard to the collection, storage, processing and disclosure of personal information and will uphold the Act's core principles. Access to collated participant data will be restricted to those clinicians treating the participants, representatives of the sponsor(s) and representatives of regulatory authorities. Computers used to collate the data will have limited access measures via user names and passwords.

Published results will not contain any personal data that could allow identification of individual participants.

Sample size calculation

We plan to recruit 2500 women to the SCREENING phase and randomise 500 women in the TREATMENT phase. We anticipate that around 58 sites (largely UK NHS sites) will be involved.

Women with a short cervix are at higher risk of preterm birth and may benefit most from pessary treatment. We aim to treat women with a cervical length less than or equal to 35mm, which we believe to be around the 30th centile. Assuming this to be correct, a sample size of 1850 screened was initially identified to generate 555 eligible for randomisation in the TREATMENT PHASE. However, following re-analysis of data (masked to treatment allocation) in September 2017 after 29 months of screening (with data on 1214 women screened), we estimated that to randomise 550 women, the sample size of screening needed to increase to 2500. Although we will encourage women to participate in the screening phase only if they wish also to participate in the treatment phase, we have allowed for a further 10% drop out after screening, so that we now aim to randomise 500 women in the treatment phase.

We plan to randomise 500 women (250 in each group). We assume a relative risk of the primary obstetric outcome (spontaneous preterm labour leading to preterm birth before 34 weeks) in the pessary group of 0.6. We believe our relative risk reduction is conservative, given a relative risk of 0.49 for delivery before 32 weeks [10] and 0.47 for delivery before 34 weeks (21% v 42%) (Liem S, personal communication) in the ProTwin study. We anticipate that 35% of women in the control group will deliver before 34 weeks. We believe that this is a conservative estimate, given that a systematic review indicated that 34.9% of women with a cervical length of 35mm or less (when scanned at 20 weeks gestation) will deliver preterm before 32 weeks (Kindinger LM et al 2015). Assuming a baseline rate of 35% and a relative risk of 0.6, a sample size of 500 has 94% power to detect a difference at the 5% significance level. If the preterm birth rate before 34 weeks is only 30%, the power drops to 88%. Both allow for losses to follow up and imperfect compliance.
For the primary neonatal outcome, Liem showed an effect size of 0.42 at the child level, and an incidence of 24% in the control group[10]. We have powered our study for a relative risk of 0.6 for the primary neonatal outcome. Assuming prevalence rates as in Liem, our study would have 97% power.

In practice, our post screening groups of women with cervical length less than or equal to 35mm is probably a lower risk than in the Liem group (less than 38mm), given comparisons of the rate of preterm delivery in each of the control groups. Hence if we assume a lower rate of the neonatal primary outcome of (say) 18% we still have 88.4% power to detect a relative risk of 0.6 in the Arabin pessary group. Such a calculation assumes analysis at the child level is appropriate for the neonatal outcome.

For the subgroup of women with cervical length of less than or equal to 25mm, the anticipated rate of the primary obstetric outcome in the control group is 82/159 (51%) [12]. The study has 85% power to detect a relative risk of 0.6 in this group, with a sample size of 234 (25mm was the 14th centile in the To et al).

Patient and public involvement.
The importance of the research question was identified by two major groups, both involving PPI. The first was the NICE guideline on Multiple Pregnancy, published in 2011, which had embedded PPI The guideline generated the following research recommendation: “What interventions are effective in preventing spontaneous preterm birth in women with twin and triplet pregnancies, especially in those at high risk of preterm birth? The second group was the Clinical Study Group from the RCOG, which has extensive lay and patient involvement. “The use of the Arabin pessary for preventing preterm birth” is one of five research priorities identified in late 2012.

There was PPI involvement in the development of the protocol at grant submission stage, including in choice of the outcome measures. Specifically, the CEO of a charity focussing on preterm birth (Jane Denton, Multiple Births Foundation) was a co-applicants on this project was and involved in the study design.

Patients and the public are also involved in the trial steering committee for this study, which oversaw the conduct of the study: both through participation of individual patients and through participation of Keith Read from Tamba (The Twins and Multiple Births Association) and two individual patients. Although PPI were not directly involved in individual patient recruitment, they provided helpful comments on the patient information sheet used for this purpose. PPI were members of the ethics committee who assessed the research burden associated with participating in this study. Individual study participants will be sent a summary of study findings, when the main study is published. We also make use of our connections with the charities Tommy’s, the Multiple Births Foundation and the Twins and Multiple Births Association to disseminate information.

Ethics
STOPPIT-2 was approved by the South East of Scotland Research Ethics Committee 2 on 29th August 2014, reference number 14/SS/1031.

Dissemination
A study report will be prepared in accordance with GCP guidelines, and submitted to the funder (NIHR) for publication in the NIHR journals library. A summary report will be submitted for publication in a peer review journal. Data will be used for publication and presentation at scientific meetings. Authorship guidelines are attached at Appendix 4.

Access to data
The Chief Investigator (JEN) and all study statisticians (JN, GMacL and DC) will have full access to the final dataset data during analysis. Access will be provided to any other author on request. There are contractual limitations on disclosures.

Data deposition
We aim to deposit the data in a shared depository. We do not anticipate providing access to the statistical analysis code.

Protocol amendments
Version 1 of the STOPPIT 2 protocol dated 22nd July 2014 was approved by the ethics committee on 29th August 2014. A list of protocol amendments is included in an Appendix 5. It is not anticipated that

For peer review only

any further substantive amendments will be made, other than addition or closure of sites. Protocol amendments are notified to, and approved by the sponsor, funder and research ethics committee, and communicated to investigators after approval. The most recent approved version of the protocol is version 4, dated 10th January 2018. One final amendment of the protocol will be submitted to address minor updates made during the preparation of this protocol paper.

References

1. Nicolaides KH, Syngelaki A, Poon LC, de Paco Matallana C, Plasencia W, Molina FS, Picciarelli G, Tul N, Celik E, Lau TK, Conturso R. Cervical pessary placement for prevention of preterm birth in unselected twin pregnancies: a randomized controlled trial. American journal of obstetrics and gynecology. 2016 Jan;214(1):3 e1-9.
2. Liem S, Schuit E, Hegeman M, Bais J, de Boer K, Bloemenkamp K, Brons J, Duvekot H, Bijvank BN, Franssen M, Gaugler I, de Graaf I, Oudijk M, Papatsonis D, Pernet P, Porath M, Scheepers L, Sikkema M, Sporken J, Visser H, van Wijngaarden W, Woiski M, van Pampus M, Mol BW, Bekedam D. Cervical pessaries for prevention of preterm birth in women with a multiple pregnancy (ProTWIN): a multicentre, open-label randomised controlled trial. Lancet. 2013 Oct 19;382(9901):1341-9.
3. Berghella V, Dugoff L, Ludmir J. Prevention of preterm birth with pessary in twins (PoPPT): a randomized controlled trial. Ultrasound Obstet Gynecol. 2017 May;49(5):567-72.
4. Norman JE, Mackenzie F, Owen P, Mactier H, Hanretty K, Cooper S, Calder A, Mires G, Danielian P, Sturgiss S, MacLennan G, Tydeman G, Thornton S, Martin B, Thornton JG, Neilson JP, Norrie J. Progesterone for the prevention of preterm birth in twin pregnancy (STOPPIT): a randomised, double-blind, placebo-controlled study and meta-analysis. Lancet. 2009 Jun 13;373(9680):2034-40.
5. Berghella V, Odibo AO, To MS, Rust OA, Althuisius SM. Cerclage for short cervix on ultrasonography: meta-analysis of trials using individual patient-level data. Obstetrics and gynecology. 2005 Jul;106(1):181-9.
6. Mangham LJ, Petrou S, Doyle LW, Draper ES, Marlow N. The cost of preterm birth throughout childhood in England and wales. Pediatrics. 2009 Feb;123(2):e312-27.
7. Chang HH, Larson J, Blencowe H, Spong CY, Howson CP, Cairns-Smith S, Lackritz EM, Lee SK, Mason E, Serazin AC, Walani S, Simpson JL, Lawn JE. Preventing preterm births: analysis of trends and potential reductions with interventions in 39 countries with very high human development index. Lancet. 2012 Nov 15.
8. Norman JE, Shennan AH. Prevention of preterm birth-why can't we do any better? Lancet. 2012 Nov 15.
9. Abdel-Aleem H, Shaaban OM, Abdel-Aleem MA. Cervical pessary for preventing preterm birth. Cochrane database of systematic reviews (Online). 2013;5:CD007873.
10. Liem S. Pessaries in multiple pregnancy as a prevention of preterm birth (ProTWIN) a randomised controlled trial. American journal of obstetrics and gynecology. 2013;208:S2.
11. Goya M, de la Calle M, Pratcorona L, Merced C, Rodo C, Munoz B, Juan M, Serrano A, Llurba E, Higueras T, Carreras E, Cabero L, Group PE-TT. Cervical pessary to prevent preterm birth in women with twin gestation and sonographic short cervix: a multicenter randomized controlled trial (PECEP-Twins). American journal of obstetrics and gynecology. 2016 Feb;214(2):145-52.
12. To MS, Fonseca EB, Molina FS, Cacho AM, Nicolaides KH. Maternal characteristics and cervical length in the prediction of spontaneous early preterm delivery in twins. American journal of obstetrics and gynecology. [Research Support, Non-U.S. Gov't]. 2006 May;194(5):1360-5.
13. Kindinger LM, Poon LC, Cacciatore S, MacIntyre DA, Fox NS, Schuit E, Mol BW, Liem S, Lim AC, Serra V, Perales A, Hermans F, Darzi A, Bennett P, Nicolaides KH, Teoh TG. The effect of gestational age and cervical length measurements in the prediction of spontaneous preterm birth in twin pregnancies: an individual patient level meta-analysis. BJOG. 2016 May;123(6):877-84.
14. Villar J, Papageorghiou AT, Knight HE, Gravett MG, Iams J, Waller SA, Kramer M, Culhane JF, Barros FC, Conde-Agudelo A, Bhutta ZA, Goldenberg RL. The preterm birth syndrome: a prototype phenotypic classification. American journal of obstetrics and gynecology. [Research Support, Non-U.S. Gov't]. 2012 Feb;206(2):119-23.
15. Curtis L. Costs of Health and Social Care Personal Social Services Research Unit, University of Kent2013.
16. Department of Health. 2011-2012 NHS Reference Costs 2013.
17. Mason J. Qualitative researching. . London: Sage Publications Ltd; 2018.

For peer review only - http://bmjopen.bmj.com/site/about/guidelines.xhtml

18. Silverman D. Doing qualitative research: a practical handbook. London: Sage publications Ltd; 2000.

Author's contributions

All authors contributed to the intellectual content of this protocol. JEN and SW wrote the first draft, with the exception of the statistical analysis plan (which was written by DC, with input from GMacL and JN), health economics (written by JS) and the qualitative research (written by SCB). All authors contributed to further drafting and approved the final version.

Funding statement

This study was funded by the NIHR Health Technology Assessment Programme (project number 13/04/22).

Sponsor

The trial co-sponsors are University of Edinburgh and NHS Lothian, ACCORD, The Queen's Medical Research Institute, 47 Little France Crescent, Edinburgh, EH16 4TJ

Neither the study sponsor not the funder has or will have any role, responsibility or authority for study design, collection, management, analysis and interpretation of data, writing or the report, or decision to submit the report for publication.

Department of Health and Social Care disclaimer

The views expressed are those of the author(s) and not necessarily those of the NHS, the NIHR or the Department of Health and Social Care

Competing interests statement

The authors declare the following competing interests:
Jane Norman is a member of the HTA Maternal, Neonatal and Child health prioritisation panel. She has performed consultancy for the pharma companies LifeArc and Dilafor outside the scope of this work.

ST provides commercial consultancy advice and is a Trustee or advisor for funding organisations.

AS is on the HTA commissioning board and has received funding from Hologic (paid to institution) for preterm prediction studies (outside the scope of this work.)

Acknowledgements

We thank Katie Morris and Fiona Mackie, both of the University of Birmingham, for provision of the Figure, and the patient advisors who helped us with the study.

For peer review only

Appendix 1.

Statistical analysis plan

For peer review only

Appendix 2.

Trial steering committee charter and membership.

For peer review only

Appendix 3

Data monitoring committee charter and membership.

For peer review only

Appendix 4
Authorship guidelines

For peer review only

Figure

Forest plot

For peer review only

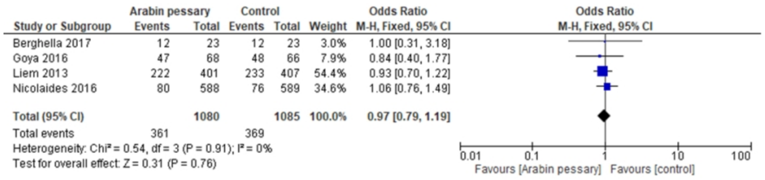

99x139mm (300 x 300 DPI)

STOPPIT-2

An open randomised trial of the Arabin pessary to prevent preterm birth in twin pregnancy, with health economics and acceptability – STOPPIT-2.

Statistical Analysis Plan
Treatment Phase
Version 1.0

Prepared by:

David Cooper

(CHaRT Statistician)

Signed: David J. Cooper

Date: 30.8.2018

Approved by:

Professor Jane Norman

(Chief Investigator)

Signed:

Date: 30th August 2018

Protocol version 5 23/8/2018

For peer review only - http://bmjopen.bmj.com/site/about/guidelines.xhtml

Contents

1 Study Objectives
1.1 Primary objective
1.2 Secondary objective

2 Study Design
2.1 Randomisation and blinding

3 Outcome Measures
3.1 Primary outcome
3.2 Secondary outcomes
3.3 Timing of outcome measurements
3.4 Adverse events

4. Sample Size and Power Calculations

5. Statistical Methods
5.1 Primary outcome
5.2 Secondary outcomes
5.3 Subgroup analyses
5.4 Missing data

6. Dummy Tables

7. References

For peer review only

List of Tables

1. Baseline clinical characteristics
2. Experience of device fitting
3. Experience of device through trial
4. Experience of device at removal
5. Primary obstetric outcome of birth before 34+0 weeks – overall and by subgroup of chorionicity and cervical length
6. Secondary obstetric outcomes – gestational age at delivery, duration of labour and duration of stay in hospital
7. Secondary obstetric outcomes – method of delivery, incidence of births before 28+0, 32+0, 34+0 and 37+0 weeks, adverse events
8. Primary composite neonatal outcome and incidence of individual components
9. Secondary neonatal outcomes – continuous outcomes
10. Secondary neonatal outcomes – categorical outcomes
11. Safety issues

For peer review only

1. Study Objectives

1.1 Primary objective

The primary objective of this study is to test if the Arabin cervical pessary reduces the risk of spontaneous preterm labour leading to preterm birth in women with a cervical length less than or equal to 35mm, who are expecting twins.

1.2 Secondary objectives

The secondary objectives of this study are to use the entire cohort of women undergoing cervical length scanning to identify the profile of cervical length measurement in women with twin pregnancy in the UK. The study also aims to obtain the positive and negative likelihood ratios of spontaneous preterm birth before 34 weeks gestation for a variety of cervical length thresholds. A separate plan will be created to describe this analysis.

For peer review only

2. Study Design

STOPPIT-2 is a multicentre, open label, randomised controlled trial of the Arabin pessary versus standard treatment (no additional treatment other than what would normally be received) in women with twin pregnancy.

The study has two phases:-
A *screening* phase in which women with a short cervix (cervical length of ≤ 35mm) will be identified;
A *treatment* phase in which women with a short cervix will be randomised to treatment with Arabin pessary or standard treatment.
Women will be seen in the antenatal clinic setting.

2.1 Randomisation and blinding

Randomisation will be carried out between 18 weeks + 0 days gestation and 20 weeks + 6 days gestation. Women with a short cervix (≤ 35mm) who agree to participate in the treatment phase of the study will be randomised to either treatment with the Arabin cervical pessary or to standard care (no additional treatment). Randomisation will be performed by the central randomisation facility based within CHaRT.

Randomisation will be minimised with a random element rather than stratified given the properties of minimisation in ensuring comparability between the groups. The minimisation variables are centre and chorionicity (monochorionic or dichorionic).

Neither the participant nor the investigator are blinded to which treatment has been allocated. The investigator will inform the woman of treatment allocation after randomisation.

3. Outcome Measures

3.1 Primary outcomes

- *Obstetric*: all births before 34 weeks + 0 days gestation following the spontaneous onset of labour. Pre-term prelabour rupture of membranes before 34 weeks with or without contractions is also included in the definition of spontaneous onset of labour.
- *Neonatal*: a composite of adverse outcomes including any death from trial entry to 28 days post expected date of delivery (miscarriage, stillbirth, neonatal or postneonatal death), periventricular

leukomalacia, early respiratory morbidity (defined as any need for supplemental oxygen >30%, CPAP [continuous positive airway pressure], or intra-tracheal ventilation or surfactant replacement therapy within the first week of life), intraventricular haemorrhage, necrotizing enterocolitis and proven sepsis. All these will be measured up to 28 days after the expected date of delivery determined by the first trimester ultrasound.

3.2 Secondary outcomes

Obstetric:

Key outcomes

- Mean gestation at delivery
- Adverse events: infection, cervical trauma
- Incidence of all births before 37+0 weeks gestation
- Acceptability of the pessary as determined by participant questionnaire
- Experience of pessary removal

Other outcomes

- Incidence of all births before each of 28+0, 32+0, and 34+0 weeks gestation
- Preterm birth before 34+0 weeks gestation preceded by preterm premature membrane rupture
- Incidence of all births before each of 28+0, 32+0, 34+0 and 37+0 weeks gestation preceded by spontaneous onset of labour
- Method of delivery (in three categories: spontaneous vaginal delivery or vaginal breech, forceps or ventouse, caesarean section)
- Duration of labour overall, and of each of the first and second stages of labour
- Duration of stay in hospital
- Other adverse events including haemorrhage, tachycardia, vaginal injury, other trauma
- Serious maternal adverse events up to 28 days after discharge from the hospital

Key *Neonatal secondary outcomes:*

- Incidence of each of the individual components of the primary neonatal outcome
- Median weight (g) of the newborn at birth
- Death of live born babies within the first 28 days after birth
- Discrete episodes of bloodstream or CNS infection (positive blood or CSF culture)
  - Within first 72 hours
  - Between 72 hours and discharge

Other neonatal outcomes

- Birth weight centile (for gestation) within 4 weeks after expected date of delivery
- Death of live born babies within the first 28 days after EDD
- Cord pH
- Apgar score at 1 and 5 minutes
- Need for resuscitation
- Need for surfactant administration

For peer review only

    - Bronchopulmonary dysplasia
    - Retinopathy of prematurity (stage 1 or more)
        - Retinopathy of prematurity requiring surgery (laser or cryotherapy)
    - Necrotising enterocolitis, (medical or surgical treatment of confirmed cases
    - Twin to twin transfusion (Quintero stage 1 or more)
- Days of respiratory support [either mechanical ventilation or CPAP]
- Days of oxygen therapy
- Daily level of care.
- Seizures requiring therapy.
- Hyperbilirubinaemia requiring exchange transfusion.
- Periventricular leukomalacia / echogenicity

4. Sample Size and Power Calculations

We plan to recruit 2500 women to the SCREENING phase and randomise 500 women in the TREATMENT phase. We anticipate that around 58 sites (largely UK NHS sites) will be involved.

We aim to treat women with a cervical length less than or equal to 35mm, which we believe to be around the 30th centile. Assuming this to be correct, a sample size of 1850 women screened was initially identified as the number of women required, to generate 555 women eligible for randomisation in the TREATMENT PHASE. However, re-analysis of data (masked to treatment allocation) in September 2017 after 29 months of screening (with data on 1214 women screened), we estimated that to randomise 550 women, the sample size of screening needed to increase to 2500. Although we will encourage women to participate in the screening phase only if they wish also to participate in the treatment phase, we have allowed for a further 10% drop out after screening, so that we aim to randomize 500 women in the treatment phase.

We plan to randomise 500 women of 500 (250 in each group). We assume a relative risk of the primary obstetric outcome (spontaneous preterm labour leading to preterm birth before 34 weeks) in the Arabin pessary group of 0.6. We believe our relative risk reduction is conservative, given a relative risk of 0.49 for delivery before 32 weeks [20] and 0.47 for delivery before 34 weeks (21% v 42%)(Liem S, personal communication) in the ProTwin study. We anticipate that 35% of women in the control group will deliver before 34 weeks. Again we believe that this is a conservative estimate, given that a systematic review indicated that 34.9% of women with a cervical length of 35mm or less (when scanned at 20 weeks gestation) will deliver preterm before 32 weeks (Kindinger LM et al 2015). Assuming a baseline rate of 35% and a relative risk of 0.6, a sample size of 500 has 94% power to detect a difference at the 5% significance level between the groups. If the preterm birth rate before 34 weeks is only 30%, power drops to 88%. Both allow for losses to follow up and imperfect compliance.
For the primary neonatal outcome, Liem showed an effect size of 0.42 at the child level, and an incidence of 24% in the control group. We have powered our study for a relative risk of 0.6 for the primary neonatal outcome. Assuming prevalence rates as in Liem, our study would have 97% power. In practice, our post screening groups of women with cervical length less than or equal to 35mm group is probably at somewhat lower risk than the Liem less than 38mm group (given comparisons of the rate of preterm delivery in each of the control groups). Hence if we assume a lower rate of the neonatal primary outcome of (say) 18% we still have 88.4% power to detect a relative risk of 0.6 in the Arabin pessary group. Such a calculation assumes analysis at the child level is appropriate for the neonatal outcome.

For the subgroup of women with cervical length of less than or equal to 25mm, the anticipated rate of the primary obstetric outcome in the control group is 82/159 (51%) [10]. The study has 85% power to detect a

For peer review only

relative risk of 0.6 in this group, with a sample size of 234 (25mm was the 14th centile in the To et al) 0.48 x sample size in < 30mm group).

5. Statistical Methods

The primary statistical analysis will be according to the intention to treat principle in which all participants will remain in their allocated group for analysis. An additional per protocol analysis will also be performed.

Statistical significance will be at the 5% level with corresponding 95% confidence intervals (CI) derived.

Randomised groups will be described at baseline and follow-up using means (with standard deviations), medians (with inter-quartile ranges) and counts (with percentages) where appropriate.

5.1 Primary outcomes

For the primary obstetric and neonatal outcomes, logistic regression with a fixed effect for the minimisation covariate chorionicity and a random effect for centre will be used to obtain the odds ratios of the treatment effect, along with the 95% confidence interval and associated p-value. In the case of the composite neonatal outcome the components will also be reported individually to show which elements are driving the primary outcome. In the case of skewed recruitment resulting in small centres a regional effect will be considered in place of a centre effect.

5.2 Secondary outcomes

Continuous secondary outcomes will be analysed using linear regression, adjusting for chorionicity. Binary categorical secondary outcomes will be analysed using logistic regression as per the primary outcomes.

Secondary outcomes with more than two categories will be analysed using multinomial logistic regression.

Analysis of fetal outcomes will allow for clustering within twins by fitting mother as a random effect in a mixed effects logistic regression model.

5.3 Subgroup analyses

Predefined subgroup analyses of the primary outcome by monochorionicity (no or yes), by cervical length (≤ 25mm) and also by cervical length ≤ 28mm will be undertaken.

A post-hoc comparison will also be performed between participants who had membrane rupture and those who did not.

In the subgroup analyses statistical significance will be at the 1% level with corresponding 99% confidence intervals (CI).

5.4 Missing Data

It is not anticipated that primary outcome data will be missing and if it is this is likely due to a miscarriage, stillbirth, neonatal or maternal death and therefore it will not be imputed.

For peer review only

6. Dummy Tables

Table 1: Baseline characteristics

Characteristic	Level	Arabin pessary N =	Standard treatment N =
Age	Mean (sd)		
	Min-Max		
Height	Mean(sd)		
	Min-Max		
Weight	Mean (sd)		
	Min-Max		
Cervical length	Mean (sd)		
	Min-Max		
Current Smoking	Yes, n (%)		
Current alcohol	Yes, n (%)		
Obstetric History			
Parity	>0, n (%)		
Miscarriage	>0, n (%)		
Medical conditions	>0, n (%)		
Hypertension	Yes, n (%)		
Insulin dependent diabetes	Yes, n (%)		
Respiratory disease	Yes, n (%)		
Cardiac disease	Yes, n (%)		
Neurological disease	Yes, n (%)		
Skin condition	Yes, n (%)		
Thrombophilia	Yes, n (%)		
Current Pregnancy			
Fetal anomaly scan: Twin 1	Normal – n (%)		
	Defined abnormality – n (%)		
	Uncertain abnormality – n (%)		
	Not done – n (%)		
Fetal anomaly scan: Twin 2	Normal – n (%)		
	Defined abnormality – n (%)		
	Uncertain abnormality – n (%)		
	Not done – n (%)		
Amniocentesis: Twin 1	Abnormal – n (%)		
Amniocentesis: Twin 2	Abnormal – n (%)		

For peer review only

Table 2: Experience of device fitting

**Arabin pessary**<br>**N =**	**At fitting**
Maternal experience of having device fitted – n (%)	
Painless	
Slightly uncomfortable	
Uncomfortable	
Very uncomfortable	
Worst pain imaginable	
Too painful to allow insertion	
Clinical team experience of fitting device – n (%)	
Easy	
Moderately easy	
Neither easy nor difficult	
Difficult	
Very difficult	
Impossible	

Table 3: Experience of device through trial

Arabin pessary<br>N =	36 week questionnaire
I could feel the pessary – n(%)	
Never	
A few times	
At least once every week	
Every day	
All the time	
I found the pessary uncomfortable – n(%)	
Never	
A few times	
At least once every week	
Every day	
All the time	
I found the pessary painful – n(%)	
Never	
A few times	
At least once every week	
Every day	
All the time	
I had vaginal discharge – n(%)	
Never	
A few times	
At least once every week	

For peer review only

Every day	
All the time	
I had vaginal bleeding – n(%)	
Never	
A few times	
At least once every week	
Every day	
All the time	
I had to use panty liners/sanitary towels because of the vaginal discharge/bleeding– n(%)	
Never	
A few times	
At least once every week	
Every day	
All the time	
The vaginal discharge was – n(%)	
The same as before the pessary	
A little more than before the pessary	
A lot more than before the pessary	
The vaginal bleeding was – n(%)	
The same as before the pessary	
A little more than before the pessary	
A lot more than before the pessary	
The vaginal discharge was – n(%)	
The same as in my last pregnancy	
A little more than in my last pregnancy	
A lot more than in my last pregnancy	
This is my first pregnancy	
The vaginal bleeding was – n(%)	
The same as in my last pregnancy	
A little more than in my last pregnancy	
A lot more than in my last pregnancy	
This is my first pregnancy	
Any other side effects experienced with the pessary	

Table 4: Experience of device removal

	**At removal**
Maternal experience of having device removed– n (%)	
Painless	
Slightly uncomfortable	
Uncomfortable	
Very uncomfortable	

For peer review only

Worst pain imaginable	
Clinical team experience of removing device – n (%)	
Easy	
Moderately easy	
Neither easy nor difficult	
Difficult	
Very difficult	

Table 5: Primary obstetric outcome of birth before 34+0 weeks – overall and by subgroup of chorionicity and cervical length

Outcome	Arabin pessary N =	Standard treatment N =	Odds ratio (95% CI)	p-value
All pregnancies				
Proportion of women delivering before 34 weeks – n (%)				*
Monochorionic pregnancy and cervical length ≤ 25mm				
Proportion of women delivering before 34 weeks – n (%)				
Dichorionic pregnancy and cervical length ≤ 25mm				
Proportion of women delivering before 34 weeks – n (%)				
Monochorionic pregnancy and cervical length ≤ 28mm				
Proportion of women delivering before 34 weeks – n (%)				
Dichorionic pregnancy and cervical length ≤ 28mm				
Proportion of women delivering before 34 weeks – n (%)				

* refers to p-value for proportion in Arabin pessary versus standard treatment group from logistic regression analysis adjusting for chorionicity

For peer review only

Table 6: Secondary obstetric outcomes – gestational age at delivery, duration of labour and duration of stay in hospital

**Outcome – mean (sd)**	**Arabin pessary N =**	**Standard treatment N =**	**Mean difference (99% CI)**	**p-value ***
Gestational age at delivery (weeks)				
Duration of labour stage 1 (mins)				
Duration of labour stage 2 (mins)				
Duration of labour overall (mins)				
Duration of hospital stay (days)				

* refers to p-value for proportion in Arabin pessary versus standard treatment group from linear regression analysis adjusting for chorionicity

For peer review only

Table 7: Secondary obstetric outcomes – method of delivery, incidence of births before 28+0, 32+0, 34+0 and 37+0 weeks, adverse events

**Outcome – n (%)**	**Arabin pessary N =**	**Standard treatment N =**	**Odds ratios (99% CI)**	**p-value ***
Method of delivery				
Spontaneous vaginal delivery				
Vaginal breech				
Forceps or ventouse				
Caesarian section				
Incidence of all births				
Before 28+0 weeks				
Before 32+0 weeks				
Before 34+0 weeks				
Before 37+0 weeks				
Incidence of births – preceded by spontaneous onset of labour				
Before 28+0 weeks				
Before 32+0 weeks				
Before 34+0 weeks				
Before 37+0 weeks				
Incidence of birth before 34+0 weeks preceded by PPMR				
Adverse events				
Infection				
Haemorrhage				
Tachycardia				
Significant maternal adverse event up to 28 days after discharge from hospital				

PPMR – preterm premature membrane rupture
* refers to p-value for proportion in Arabin pessary versus standard treatment group from logistic regression analysis adjusting for chorionicity or using proportional odds analysis

For peer review only

Table 8: Primary composite neonatal outcome and incidence of individual components

**Outcome – n (%)**	**Arabin pessary N =**	**Standard treatment N =**	**Odds ratio (95% CI)**	**p-value**
Composite neonatal outcome				
Individual components				
Stillbirth or neonatal death				
Periventricular leukomalacia				
Early respiratory morbidity				
Intraventricular haemorrhage				
Necrotizing enterocolitis				
Proven sepsis				

Data refer to all twins with the 95% CI and p-values adjusted for clustering within twins

For peer review only

Table 9: Secondary neonatal outcomes – continuous outcomes

**Outcome – mean (sd)**	**Arabin pessary N =**	**Standard treatment N =**	**Mean difference (99% CI)**	**p-value**
Birth weight centile within 4 weeks after expected d.o.d.				
Weight of newborn at birth				
Cord pH				
Agpar score at 1 minute				
Agpar score at 5 minutes				
Days of oxygen therapy				
Level of care days				

d.o.d. – date of delivery
Data refer to all twins with the 99% CI and p-values adjusted for clustering within twins

Table 10: Secondary neonatal outcomes – categorical outcomes

**Outcome – n (%)**	**Arabin pessary N =**	**Standard treatment N =**	**Odds ratio (99% CI)**	**p-value**
Need for resuscitation				
Fetal or neonatal death within the first 28 days after birth				
Need for surfactant administration				
Bronchopulmonary dysplasia				
Necrotising enterocolitis				
Discrete episodes of bloodstream or CNS infection				
Daily level of care				
Rate of major adverse neonatal outcomes before discharge from hospital				

Data refer to all twins with the 99% CI and p-values adjusted for clustering within twins

For peer review only

Table 11: Safety Issues – p-value from Fisher's Exact test on subjects

**Type**	**Arabin pessary N =**	**Standard treatment N =**	**p-value from exact test**
Mother died			
Intrauterine death			
Neonatal death			
Involved or prolonged inpatient maternal hospitalisation			
Involved persistent/significant maternal disability/incapacity			
Life threatening			
Chorioamnionitis or intrauterine infection			
Congenital anomaly/birth defect			

For peer review only

7. REFERENCES

1. To, M. S., E. B. Fonseca, F. S. Molina, A. M. Cacho and K. H. Nicolaides (2006). "Maternal characteristics and cervical length in the prediction of spontaneous early preterm delivery in twins." American journal of obstetrics and gynecology 194(5): 1360-1365.

2. Liem S et al (2013). "Cervical pessaries for prevention of preterm birth in women with a multiple pregnancy (ProTWIN): a multicentre, open-label randomised controlled trial". The Lancet 382(9901): 1341-1349.

3. Norman, J. E., F. Mackenzie, P. Owen, H. Mactier, K. Hanretty, S. Cooper, A. Calder, G. Mires, P. Danielian, S. Sturgiss, G. MacLennan, G. Tydeman, S. Thornton, B. Martin, J. G. Thornton, J. P. Neilson and J. Norrie (2009). "Progesterone for the prevention of preterm birth in twin pregnancy (STOPPIT): a randomised, double-blind, placebo-controlled study and meta-analysis." Lancet 373(9680): 2034-2040.

4. Kindinger LM, Poon LC, Cacciatore S, MacIntyre DA, Fox NS, Schuit E, Mol B, Liem S, Lim A, Serra V, Perales A, Hermans F, Darzi A, Bennett P, Nicolaides KH, Teoh TG (2015). The prediction of preterm birth in twin pregnancy: an individual patient level meta-analysis. BJOG in press.

For peer review only

STOPPIT-2 study

Trial Steering Committee Charter

Version 2.0 Date 22nd July 2015

**Study Title:**	An open randomised trial of the Arabin pessary to prevent preterm birth in twin pregnancy, with health economics and acceptability – STOPPIT-2.
**Chief Investigator:**	**Professor Jane Norman**
**REC Number:**	**14/SS/031**
**Sponsor:**	University of Edinburgh & NHS Lothian

For peer review only

Approval Signatures:

The following individuals, by providing their signatures, indicate their understanding of and willingness to comply with the roles and responsibilities assigned to them in this Charter.

1. TSC independent Chair:

________________	__________________	_ _ / _ _ /_ _ _ _
PRINT NAME	SIGNATURE	DATE

2. TSC independent Member:

________________	__________________	_ _ / _ _ /_ _ _ _
PRINT NAME	SIGNATURE	DATE

3. TSC independent Member:

________________	__________________	_ _ / _ _ /_ _ _ _
PRINT NAME	SIGNATURE	DATE

4. Chief Investigator:

________________	__________________	_ _ / _ _ /_ _ _ _
PRINT NAME	SIGNATURE	DATE

5. Trial Statistician:

________________	__________________	_ _ / _ _ /_ _ _ _
PRINT NAME	SIGNATURE	DATE

For peer review only

CONTENTS

CONTENTS 1
1. Introduction 3
2. Membership of the TSC 3
2.1. Members: 4
2.2. Agreements 6
2.3. Indemnity 6
2.4. Payments 6
3. Responsibilities 6
4. Interaction between TSC and other study committees 8
5. Scheduling, Quorum, and Organization of Meetings 8
5.1. Attendance at meetings 9
5.2. Non-attendance at meetings 9
5.3. Report for the TSC 9
5.4. Contents of the TSC Report 10
6. Decision making 10
6.1. Recommendations 10
6.2. TSC records 11
7. Trial closure 11
9. Publication 12
10. References: 12

For peer review only

For peer review only - http://bmjopen.bmj.com/site/about/guidelines.xhtml

Abbreviations and glossary

CI	Chief Investigator
DMC	Data Monitoring Committee
eCRF	Electronic case report form
GCP	Good clinical practice
ISRCTN	International standard randomised controlled trial number
TSC	Trial Steering Committee
PMG	Project Management Group

For peer review only

For peer review only - http://bmjopen.bmj.com/site/about/guidelines.xhtml

1. Introduction

The role of the Trial Steering Committee (TSC) is to act as the oversight body for this trial on behalf of the Sponsors and the Trial Funder and to ensure that the trial is conducted according to the guidelines for Good Clinical Practice (GCP), Research Governance Framework for Health and Social Care and all relevant regulations and local policies.

The background to this trial and the trial objectives are described in the protocol. The purpose of this document is to describe the membership, terms of reference, roles, responsibilities, authority, decision-making and relationships of the Trial Steering Committee (TSC) for this trial, including the timing of meetings, methods of providing information to and from the TSC, frequency and format of meetings and relationships with other trial committees. The charter also describes the procedures for ensuring confidentiality and proper communication to and from the TSC and an outline of the content of the reports to be provided to the TSC.

2. Membership of the TSC

The STOPPIT-2 Trial TSC is an independent multidisciplinary group that, collectively, have experience/expertise in the management of patients with condition(s) relevant to study and anticipated adverse effects and in the conduct and monitoring of randomised clinical trials. The role of the TSC is to provide oversight for the trial and provide advice through its independent Chair to the Project Management Group (PMG) on all aspects of the trial.

For peer review only

For peer review only - http://bmjopen.bmj.com/site/about/guidelines.xhtml

2.1. Members

(Names redacted from this version)

INDEPENDENT (VOTING) MEMBERS

TSC Independent Chair
Neonatologist

TSC Independent Member
Obstetrician

TSC Independent Member
Patient Advisory Group (PAG) Representative

TSC Independent Member
Statistician

TSC Independent Member:
Charity lead

TSC Independent Member
Patient Advisory Group (PAG) Representative

For peer review only

NON-INDEPENDENT MEMBERS

Representative of the Sponsor (Non-Voting)

Chief Investigator
Professor Jane Norman

The Queen's Medical Research Institute
47 Little France Crescent
Edinburgh
EH16 4TJ
Tel: 0131 242 2694

Email:jane.norman@ed.ac.uk;

The Facilitator (Non-Voting)

Members of the Project Management Group (PMG) who may be invited to attend as non-voting members:

Trial Manager

Trial Statistician

NB: Other members of the Project Management Group (PMG) are invited at the discretion of the TSC Chair.

FUNDERS CONTACT:
Programme manager

For peer review only

2.2. Agreements

TSC independent members will be asked to formally acknowledge their agreement to join the group by signing the TSC charter indicating that they:

(1) agree to be a member of the TSC

(2) agree with the contents of this Charter

(3) and have declared any potential conflicts of interest.

TSC members should not have any apparent financial, scientific or intellectual conflict of interest (see tables 1 and 2) that could prevent them from objectively reviewing the study protocol, interim and final data and giving advice to the PMG. TSC members should disclose to the Chair any other conflicts they consider relevant. Any members who develop significant conflicts of interest during the course of the trial should inform the Chair and may need to consider resigning from the TSC.

2.3. Indemnity

The University of Edinburgh insurance indemnifies TSC members for their work on the committee.

2.4. Payments

Standard travel and subsistence costs only will be paid to members of the TSC. Representatives of the Patient Advisory Group (PAG) may receive payments for their time in attending meetings of the TSC as appropriate to their role as a member of the PAG. No other payments or rewards will be given to members.

3. Responsibilities

The TSC members, on behalf of the Sponsor and Funder, will have overall responsibility for the conduct of the trial and for safeguarding the rights, safety and wellbeing of participants. The Chair has previous experience of serving on trial committees and experience of Chairing meetings, and will facilitate and summarise discussions. Responsibilities of the TSC include:

- Reviewing selection/recruitment/retention of participants and their management.
- Finalising and reviewing study protocol and other study documentation.
- Determine if amendments to the protocol or changes to study conduct are required and deciding on changes to these and to study conduct in general. Any changes to trial documentation or conduct must be notified to the TSC.

For peer review only - http://bmjopen.bmj.com/site/about/guidelines.xhtml

- Reviewing adherence to the protocol by Investigators and participants.
- Assessing the impact and relevance of external evidence.
- Assessing integrity and completeness of data collected.
- Monitoring the overall conduct of the trial, ensuring that it follows the standards set out in the guidelines of GCP, assessing the safety and efficacy of the interventions, recruitment figures and completion of trial assessments.
- Reviewing, commenting and making decisions on extension requests.
- Reviewing the recommendations of the DMC (Data Monitoring Committee) (if applicable) and/or other study committees and suggesting appropriate action to the PMG.
- Monitoring the progress of study/trial and deciding on appropriate action in order to maximise the chances of completing it within the agreed timelines.
- Considering new information relevant to the study e.g. results from other studies that may have a bearing to the conduct of the study and deciding on appropriate action.

Additional responsibilities may include:

1. Endorsing the annual report to the funder and ethics (if required).
2. Approving proposed protocol amendments or new trial sub-studies.
3. Approving requests for early release of (subsets of) data.
4. Approving external applications for the use of stored samples (if applicable).
5. Approving presentation of results during the trial or soon after closure.
6. Approving strategies to improve recruitment or follow-up.

For peer review only

4. Interaction between TSC and other study committees

The responsibilities of each trial committee are outlined in the protocol and in the respective Charters. The relationships between these groups are summarised in Figure 1 below.

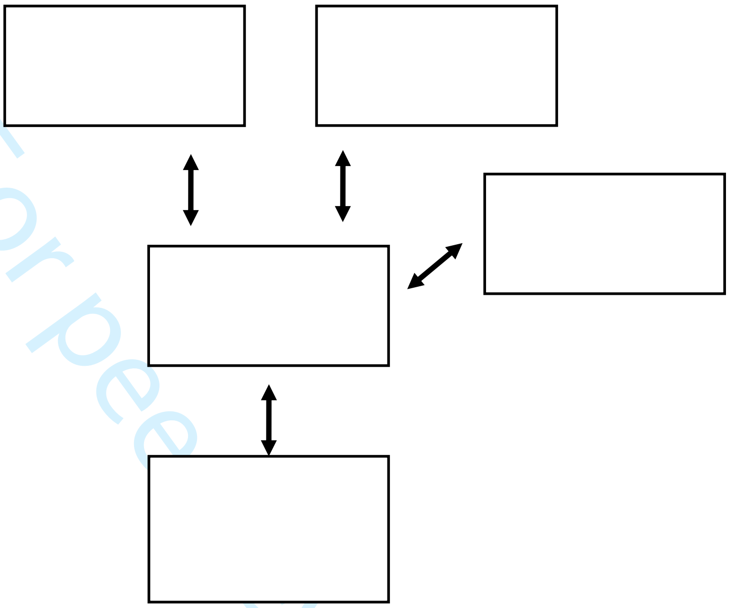

5. Scheduling, Quorum, and Organization of Meetings

The responsibility for calling and organising TSC meetings lies with the Chief Investigator in association with the Chair. The Chair, assisted by the Chief Investigator, is responsible for facilitating the meetings and summarising discussions. The Facilitator will be responsible for the organisation of meetings and should be copied into all communications with and between the TSC.

A meeting of the TSC is considered quorate if at least four independent members, including the Chair (unless otherwise agreed), take part, plus a member of the trial team.

The TSC will aim to meet in person at least yearly. The TSC should be available to provide independent advice as required, not just when meetings are scheduled. Major trial issues may need to be dealt with between meetings, by phone or by email. The TSC should maintain confidentiality of all information it receives. Members should not discuss confidential issues from their involvement in the study until the primary results have been published.

For peer review only

For peer review only - http://bmjopen.bmj.com/site/about/guidelines.xhtml

5.1. Attendance at meetings

Meetings will usually be limited to the TSC members (Section 2), observers from the sponsors, invited PMG members and the Facilitator. Other attendees may be invited for all or part of the meeting by the TSC. The observers are not members of the TSC but may be invited to provide expert input; other observers will be at the discretion of the TSC and the Facilitator but may include members of the PMG other than the CI.

Effort will be made to ensure that all members can attend. The Facilitator will work for a date that enables this. The CI must try to attend all meetings, especially if major actions are expected. Members who cannot attend in person should be encouraged to participate by teleconference. TSC members who will not be able to attend the meeting may pass comments to the TSC Chair via the Facilitator for consideration during the discussions. If, at short notice, any TSC members cannot attend then the TSC may still meet if at least four independent members, including the Chair (unless otherwise agreed), will be present, plus also a member of the trial team. If the TSC is considering a major action after such a meeting the TSC Chair should communicate with the absent members, including the CI, as soon after the meeting as possible to check they agree. If they do not, a further teleconference should be arranged with the full TSC.

5.2. Non-attendance at meetings

If an independent member does not attend a meeting or provide comments when requested between meetings, it will be ensured that the independent member is available for the next meeting. If an independent member does not attend the next meeting or provide comments when next requested, they will be asked if they wish to remain part of the TSC. If an independent member does not attend a third meeting, strong consideration will be given to replacing this member.

The TSC membership is for the duration of the trial. If any members leave the TSC, the PMG and TSC should provide recommendations for replacements promptly.

5.3. Report for the TSC

A report will be prepared by the trial manager on behalf of the PMG containing accrual and any matters affecting the trial. Additionally, the material may include a report *from* the DMC, requests *from* the PMG or draft publications. No trial outcome measure data will be presented by arm unless explicitly authorised by the DMC. Where relevant, accrual, compliance with follow-up and adherence to treatment may be presented by centre. The TSC will receive the report at least 1 week but preferably 2 weeks before any meetings. Different procedures may apply to teleconference meetings.

For peer review only - http://bmjopen.bmj.com/site/about/guidelines.xhtml

TSC members are expected to store securely copies of the reports to and from the TSC, agenda and minutes, as well as copies of communications between meetings. All documentation should be considered confidential.

5.4. Contents of the TSC Report

An outline example of the contents of the TSC report is given below:

- Major protocol amendments
- Patient screening
- Eligibility violations
- Protocol deviations/violations by investigators or participants
- Study accrual by month/total
- Completeness and quality of data collected/eCRF return
- Quality controls
- Safety reporting
- Any matters affecting the trial
- Compliance by patients to clinic visit
- Latest DMC recommendations

6. Decision making

All potential independent TSC members will have the opportunity to comment on the protocol as early as possible. Before recruitment begins the trial will have undergone review by the Sponsor/Funder (e.g. peer review for public sector trials), scrutiny by other trial committees and a research ethics committee. Therefore, if a potential independent TSC member has major reservations about the trial (e.g. the protocol, the logistics, ethical concerns) they should report these to the Chief Investigator and may decide not to accept the invitation to join. TSC members will be constructively critical of the ongoing trial, but also supportive of aims and methods of the trial.

6.1. Recommendations

On consideration of the information presented at these meetings, the TSC should provide recommendations of appropriate action in writing to the PMG who will be responsible for implementing any actions. The role of the Chair is to summarise discussions and encourage consensus. Therefore it is best for the chair to give his/ her own opinion last. It is important that the implications (ethical, statistical, practical and financial) for the trial be considered before any decision is made. Decisions and recommendations by the TSC should be unanimous, if not a vote may be taken.

The TSC should inform the PMG if:

For peer review only

- There are concerns about the safety of participants, recruitment, the integrity of trial data or adherence to the protocol.
- The TSC may also provide feedback to the DMC and where appropriate to the Sponsor/Funder.

The DMC may make recommendations to the TSC based on the interim data. Identification and circulation of published external evidence (e.g. from other trials/ systematic reviews) is not the responsibility of the TSC members; it is a responsibility of the PMG. However, the TSC should continue to be made aware of other data that may impact on the trial.

The TSC is the oversight body for the trial. However, the TSC should have good reason(s) before deciding not to accept requests from the PMG and recommendations from the DMC. If there are serious problems or concerns with the TSC decision following a DMC recommendation, a joint meeting of the TSC and DMC should be held. The information to be shown would depend upon the action proposed and each committee's concerns. Depending on the reason for the disagreement confidential data may have to be revealed to all or some of those attending such a meeting: this would be minimised where possible. The meeting will ideally be Chaired by an external expert who is not directly involved with the trial.

6.2. TSC records

The Minutes of the meeting including key points and actions will be prepared by the facilitator. The Facilitator will keep a central record of all minutes, reports and correspondence by the TSC in the Trial Master File. These minutes will describe the proceedings and include the recommendations of the TSC. All members of the TSC must agree the minutes and these will be signed off by the TSC Chair on behalf of all members. Minutes will be circulated to all TSC members, the PMG, the Sponsor and, if appropriate, the Trial Funder. Approved Minutes will also be filed in the Trial Master File.

7. Trial closure

The TSC, based on recommendations from the DMC, may recommend early termination of the trial or modification of the study design in the event of a clear accumulating data or on the basis of information available from other sources or on safety grounds. Possible decisions include:-

1. No action needed, trial continues as planned.

For peer review only - http://bmjopen.bmj.com/site/about/guidelines.xhtml

2. Early stopping due, for example, to overwhelming evidence benefit or evidence of harm of a treatment or external evidence (this should generally involve a recommendation from the DMC to unblind the TSC to these data).
3. Modifying target recruitment, or pre-analysis follow-up, based on any change to the assumptions underlying the original trial sample size calculation (but not on any emerging differences).
4. Sanctioning and/or proposing protocol changes.

9. Publication

Manuscripts that arise from the trial will be shared with the TSC and members will be able to comment. The TSC members and their affiliations will be acknowledged in reports of the trial.

10. References

The charter was developed using:

1. MRC Clinical Trials Unit template TSC Charter version 1.02, 13-Mar-2006
2. CCTU/GD018 Version No.1 Approved: 21/03/2013 http://www.cuh.org.uk/sites/default/files/research/CCTU_GD018%20Trial%20Steering%20Committee%20or%20Study%20Steering%20Committee%20Guidance.pdf accessed 24/04/2014
3. MRC Guidelines for GCP in clinical trials (1998) issued 01/Sep/2006 http://www.mrc.ac.uk/Utilities/Documentrecord/index.htm?d=MRC002416 accessed 24/04/2014
4. MHRA GCP Guide (2012)
5. Conroy et al.: Trial steering committees for randomised controlled trials: updating and redeveloping guidance and terms of reference informed by current practice and experience. Trials 2013 14 (Suppl 1):P128.

For peer review only

Table 1: Potential competing interests for independent members

- Stock ownership in any commercial companies involved
- Stock transaction in any commercial company involved (if previously holding stock)
- Consulting arrangements with the Sponsor/Funder
- Frequent speaking engagements on behalf of the intervention
- Career tied up in a product or technique assessed by trial
- Hands-on participation in the trial
- Involvement in the running of the trial
- Emotional involvement in the trial
- Intellectual conflict e.g. strong prior belief in the trial's experimental arm
- Involvement in regulatory issues relevant to the trial procedures
- Involvement in the writing up of the main trial results in the form of authorship

Table 2: Potential competing interests for non-independent members

- Stock ownership in any commercial companies involved
- Stock transaction in any commercial company involved (if previously holding stock)
- Consulting arrangements with the Sponsor/Funder
- Frequent speaking engagements on behalf of the intervention
- Intellectual conflict e.g. strong prior belief in the trial's experimental arm

For peer review only

For peer review only - http://bmjopen.bmj.com/site/about/guidelines.xhtml

Academic and Clinical Central Office for Research and Development

STOPPIT-2

Data Monitoring Committee Charter

**Study Title:**	An open randomised trial of the Arabin pessary to prevent preterm birth in twin pregnancy, with health economics and acceptability – STOPPIT-2.
**Chief Investigator:**	**Professor Jane Norman**
**REC number:**	**14/SS/031**
**Sponsor:**	University of Edinburgh & NHS Lothian

For peer review only

For peer review only - http://bmjopen.bmj.com/site/about/guidelines.xhtml

Approval Signatures:

The following individuals, by providing their signatures, indicate their understanding of and willingness to comply with the roles and responsibilities assigned to them in this Charter.

1. DMC Chair:

________________	________________	_ _/ _ _/_ _ _ _
PRINT NAME	SIGNATURE	DATE

2. DMC Member:

________________	________________	_ _/ _ _/_ _ _ _
PRINT NAME	SIGNATURE	DATE

3. DMC Member:

________________	________________	_ _/ _ _/_ _ _ _
PRINT NAME	SIGNATURE	DATE

4. Chief Investigator:

________________	________________	_ _/ _ _/_ _ _ _
PRINT NAME	SIGNATURE	DATE

5. [Trial/Unblinded] Statistician/Analyst:

________________	________________	_ _/ _ _/_ _ _ _
PRINT NAME	SIGNATURE	DATE

For peer review only

For peer review only - http://bmjopen.bmj.com/site/about/guidelines.xhtml

Table of Contents

1. Introduction 4
2. Primary responsibilities of the DMC 4
3. Membership of the DMC 4
   3.1 Members 4
   3.2 Conflicts of interest 5
4. Timing and purpose of the DMC meetings 5
   4.1 Organisational meeting and early safety/trial integrity reviews 5
   4.2 DMC meetings 6
5. Procedures to ensure confidentiality & proper communication 6
   5.1 Closed sessions 6
   5.2 Open sessions 7
   5.3 Open and Closed reports 7
   5.4 Minutes of the DMC meeting 7
   5.5 Recommendations to the Trial Steering Committee 8
6. Statistical monitoring guidelines 8
7. Content of the DMC's open and closed reports 9
   7.1 Open statistical report: outline 9
   7.2 Closed statistical report: outline 9
8. DMC Payments 10
9. References 10

For peer review only - http://bmjopen.bmj.com/site/about/guidelines.xhtml

1. Introduction

This Charter is for the Data Monitoring Committee (DMC) for the STOPPIT-2 An open randomised trial of the Arabin pessary to prevent preterm birth in twin pregnancy, with health economics and acceptability. This study aims to determine whether the Arabin cervical pessary prevents preterm birth in women with a twin pregnancy and a short cervix. Women with a twin pregnancy are at high risk of preterm labour. The extra risk of preterm labour in twin pregnancy is the main reason why twin babies are five times more likely to die, both within the first month or the first year of life. Preterm babies who survive are at increased risk of long term disability, and often require extensive and costly health care, so if preterm birth could be prevented in twins this would be extremely beneficial.

The Charter will define the primary responsibilities of the DMC, its membership, and the purpose and timing of its meetings. The Charter will also provide the procedures for ensuring confidentiality and proper communication, the statistical monitoring guidelines to be implemented by the DMC, and an outline of the content of the Open and Closed Reports that will be provided to the DMC.

2. Primary responsibilities of the DMC

The DMC will be responsible for safeguarding the interests of trial participants, assessing the safety of the interventions during the trial, reviewing external evidence with an impact on risk/benefit balance and for monitoring the overall conduct of the clinical trial. The DMC will provide recommendations about safety, modifying or continuing the trial to the Trial Steering Committee (TSC). To contribute to enhancing the integrity of the trial, the DMC may also formulate recommendations relating to the selection, recruitment, or retention of participants, or their management, or to improving their adherence to protocol-specified regimens and retention of participants, and the procedures for data management and quality control.

The DMC will be advisory to the TSC. The TSC will be responsible for promptly reviewing the DMC recommendations, to decide whether to continue or terminate the trial, and to determine whether amendments to the protocol or changes in study conduct are required.

3. Membership of the DMC

3.1 Members

The DMC is an independent multidisciplinary group consisting of clinicians, statisticians and methodologists that, collectively, have experience/expertise in the management of patients with the condition relevant to study and anticipated adverse effects and in the conduct and monitoring of

For peer review only - http://bmjopen.bmj.com/site/about/guidelines.xhtml

randomised clinical trials. University of Edinburgh insurance indemnifies DMC members for their work on the committee.

DMC: Chair (Independent statistician)

DMC: Independent Clinical Investigator (Obstetrician)

DMC: Independent Clinical Investigator (Neonatologist)

DMC: (non-Independent) statistician/analyst (TBC)

(Note – names removed in this version to be published)

3.2 Conflicts of interest

Independent DMC membership is restricted to individuals free of relevant significant conflicts of interest. The source of these conflicts may be financial, scientific or regulatory in nature. Individuals who fulfil any of the following criteria are automatically disqualified from membership: the study investigators, individuals with conflicts of interest as determined by the Chair of the DMC.

The DMC members will disclose to fellow members any consulting agreements or financial interests they have with Edinburgh University, NHS or the manufacturing license holder of an investigational medicinal product/medical device subject to investigation during the study. The DMC will be responsible for deciding whether these consulting agreements or financial interests materially impact their objectivity.

The DMC members will be responsible for advising fellow members of any changes in these consulting agreements and financial interests that occur during the course of the trial. Any DMC member who thinks they may have developed a relevant significant conflict of interest during the course of the trial must declare this. If the other DMC members consider it to be a relevant significant conflict of interest, the member should resign from the DMC.

DMC membership is normally for the duration of the clinical trial. If any member leaves the DMC during the course of the trial, the sponsor, in consultation with the TSC and/or investigators will promptly appoint their replacement.

4. Timing and purpose of the DMC meetings

4.1 Organisational meeting and early safety/trial integrity reviews

This charter was prepared early in the course of the trial (January 2015) by the trial sponsors, trial chief investigator, trial statistician, to be approved by the DMC at their first meeting. At the first meeting the DMC will provide an advisory review of scientific and ethical issues relating to study design and conduct, discuss the Charter for the role and functioning of the DMC, and the format

For peer review only - http://bmjopen.bmj.com/site/about/guidelines.xhtml

and content of the Open and Closed Reports that will be used to present trial results at future DMC meetings.

4.2 DMC meetings

The 1st meeting of the DMC has been scheduled before recruitment of the first participant, to discuss the protocol, the charter and the remit of the DMC, and to have the opportunity to clarify any aspects with the principal investigators, including the format of DMC reports from the Study Data Centre at the CHaRT Clinical Trials Unit.

Future meetings will be held at times to be determined by the DMC e.g. after a certain number of patients have been recruited, generally by teleconference, but face-to-face if necessary. The date of each meeting will be made available to the [unblinded/trial] statistician/analyst. The trial chief investigator and trial statistician/analyst will be available for an open session at the beginning of the meeting, and will be available at the end of the meeting to answer any urgent questions.

5. Procedures to ensure confidentiality & proper communication

To enhance the integrity and credibility of the trial, procedures will be implemented to ensure the DMC has sole access to emerging information from the clinical trial regarding safety data, aggregated by treatment arm. [An exception will be made to permit access to an unblinded statistician who will be responsible for creating the closed report and sending it to the DMC.] The sponsors will provide the chair of the DMC with information on any serious unexpected adverse device reactions to the study treatment, and will also be responsible for satisfying the standard requirements for reporting of relevant events to the appropriate authorities.

At the same time, procedures will be implemented to ensure proper communication is achieved between the DMC and the trial investigators and sponsor. To provide a forum for exchange of information among various parties that share responsibility for the successful conduct of the trial, a format for Open Sessions and Closed Sessions will be implemented. The intent of this format is to enable the DMC to preserve confidentiality of the unblinded results while at the same time providing opportunities for interaction between the DMC and others who have valuable insights into trial-related issues.

All materials, discussions and proceedings of the DMC are completely confidential.

5.1 Closed sessions

Sessions involving only DMC membership (but often including the unblinded statistician as well, as a non-voting member) called Closed Sessions will be held to allow discussion of confidential data from the clinical trial, including information about the safety of interventions. In order to ensure that the DMC will be fully informed in its primary mission of safeguarding the interest of participants, the DMC will be unblinded in its assessment of safety data. During these sessions, the DMC will

For peer review only - http://bmjopen.bmj.com/site/about/guidelines.xhtml

develop a consensus on its list of recommendations, including that relating to whether the trial should continue.

DMC members and all other participants in the closed section of DMC meetings and the production of unblinded reports are expected to maintain confidentiality, and will refrain from revealing to the TSC, or any other party, information that would lead to compromising the integrity of the trial unless such release is required to protect patient safety.

5.2 Open sessions

In order to allow the DMC to have adequate access to information provided by the trial investigators, or by members of the regulatory authorities, a joint session between these individuals and DMC members (called an Open Session) will be held before the Closed Session. If necessary, a further Open Session can be held, on request either in the middle or end of the Closed Session. Open sessions give the DMC an opportunity to query these individuals about issues that have arisen during their review in the initial Closed Session. With this format, important interactions are facilitated through which problems affecting trial integrity can be identified and resolved. These individuals will either be present at the DMC meeting or be provided a telephone link.

5.3 Open and Closed reports

For each DMC meeting, Open and Closed Reports will be provided (see Section 7 for outlines of the content of these reports). Open Reports, available to all who attend the DMC meeting, will include data on recruitment and pooled data on baseline characteristics, eligibility violations, completeness of follow-up and compliance. The trial manager and the trial statistician/analyst will supervise preparation of these Open Reports.

Closed Reports, available only to those attending the Closed Sessions of the DMC meeting, will include the same information plus adverse events by allocation. The trial statistician/analyst will produce the Closed Reports to the DMC.

The Open and Closed Reports should provide information that is as accurate as possible at the time of preparation, with follow-up that is as complete as possible to within approximately one month of the date of the DMC meeting. The Reports should be provided to DMC members at least one week prior to the date of the meeting.

5.4 Minutes of the DMC meeting

The [DMC Chair/other designated DMC member] will prepare minutes of the DMC meetings. Two sets will be prepared: The Open Minutes and the Closed Minutes.

The Open Minutes will describe the proceedings in the Open Session of the DMC meeting, and will summarize all recommendations by the DMC. Since these minutes will be circulated to the trial co-

For peer review only - http://bmjopen.bmj.com/site/about/guidelines.xhtml

For peer review only

chief investigators, it is essential that these minutes do not unblind any results, if the DMC is not recommending early termination.

The Closed Minutes will describe the proceedings from all sessions of the DMC meeting, including the listing of recommendations by the Committee. Because it is likely that these minutes will contain unblinded information, it is important that they are not made available to anyone outside the DMC. Rather, copies will be kept by [the DMC chair/other designated DMC member]. These will be sent to the trial manager and archived at the time of study closure.

5.5 Recommendations to the Trial Steering Committee

At each meeting of the DMC during the conduct of the trial, the DMC will make a recommendation to the TSC. Possible recommendations include:

- Trial continues as per protocol
- Early termination of the trial (or a recommendation for the TSC to review unblinded data), if significant safety issues arise
- Extending recruitment or extending follow-up

The recommendation(s) will be based primarily on safety considerations and will be guided by statistical monitoring guidelines defined in this Charter. Should the DMC decide to recommend early termination of the trial, a full vote of the independent DMC members will be required. All members of the DMC will be required for this to be quorate. In the event of a split vote, the decision will go with the majority vote, but a report should be provided to the TSC, detailing the split. This report should not include unblinded data unless deemed necessary by the DMC. This information should be forwarded to the trial chief investigator as rapidly as possible.

The TSC is jointly responsible with the DMC for safeguarding the interests of participants and for the conduct of the trial. Recommendations to amend the protocol or conduct of the study made by the DMC will be considered and either accepted or rejected by the TSC. The TSC will be responsible for deciding whether to continue or to stop the trial based on the DMC recommendations. The DMC will be notified of all changes to the protocol or to study conduct. The DMC concurrence will be sought on all substantive recommendations or changes to the protocol or study conduct prior to their implementation.

6. Statistical monitoring guidelines

During the period of recruitment into the study, information that is available on safety outcomes such as serious adverse device events believed to be related to treatment will be supplied, in strictest confidence, to all members of the DMC, along with any other analyses that the DMC may request.

For peer review only - http://bmjopen.bmj.com/site/about/guidelines.xhtml

7. Content of the DMC's open and closed reports

7.1 Open statistical report outline:

i. Report Information (milestones/gannt chart, date of data lock etc.)

ii. Protocol amendments and update on study documentation

iii. Site/central monitoring report

iv. Adherence to protocol (list of protocol deviations/violations)

v. Recruitment update/uptake rate

vi. Completeness of data collected (reporting missing data 'forms' overall and by site)

vii. Quality of data collected (reporting no. of queries/worst 'offenders' likewise)

viii. Safety reporting - list of Serious Adverse Events/SUSARs

ix. Withdrawals (i.e. adherence to intervention & retention)

   a. from intervention (plus reasons)

   b. from trial data collection i.e. losses to follow-up (plus reasons)

x. Other issues

xi. Recent relevant publications

7.2 Closed statistical report outline:

i. Report Information (date of data point/lock)

ii. Baseline comparability (for minimisation factors by group)

iii. Interim results for primary outcome(s) and selected secondary outcomes (either safety or process)

iv. Safety reporting – Unexpected Serious Adverse Device events/USADE (by group)

v. Safety reporting - list of Serious Adverse Events/SUSARs (to review event rates)

vi. Withdrawals (adherence to intervention & retention) (by group)

   i. from intervention (plus reasons)

   ii. trial data collection & follow-up (plus reasons)

For peer review only - http://bmjopen.bmj.com/site/about/guidelines.xhtml

8. DMC Payments

DMC members will be reimbursed for travel and accommodation. No other payments or rewards are anticipated but their contribution will be acknowledged where relevant in publications.

9. References

1. DAMOCLES Study Group. A proposed charter for clinical trial data monitoring committees: helping them to do their job well. The Lancet 2005; vol 365: 711-722.

For peer review only

For peer review only - http://bmjopen.bmj.com/site/about/guidelines.xhtml

STOPPIT 2 Publication guidelines

All relevant contributors will be acknowledged (by name where possible) in ensuing publications. Authorship will be assigned according to journal or ICMJE guidelines (see http://www.icmje.org/).

Briefly, ICMJE suggest that authorship credit should be based on: 1) substantial contributions to conception and design, acquisition of data, or analysis and interpretation of data; 2) drafting the article or revising it critically for important intellectual content; and 3) final approval of the version to be published. Authors should meet conditions 1, 2, and 3.

According to these guidelines therefore the sole activity of recruitment of participants or supervision of recruitment of participants, whilst crucially important, will qualify for contributorship, but is unlikely to qualify for authorship. The original grant applicants will qualify for authorship, as long as they continue to be involved in the study, and can also therefore satisfy conditions 2 and 3 above.

Final decisions on publication and authorship will be made by the trial steering committee.

For peer review only

Appendix 5 **PROTOCOL REVISIONS**

PROTOCOL VERSION	DATE	REASON FOR UPDATE	SUBSTANTIAL AMENDMENT NUMBER	SUMMARY OF CHANGES
2	02nd June 2015	Text reformatted and clarified, information updated<br>Questionnaires created and updated	1	**General updates/reformatting**<br>Clarification of secondary endpoints (obstetric/neonatal)<br>**Additional clinical information to be collected, to allow sensitivity analyses in future individual patient data meta-analyses**<br>Internal pilot phase interviews refined and made specific<br>Inclusion/exclusion criteria updated<br>Clarification of withdrawal<br>Pessary criteria updated<br>Ultrasound accreditation method updated<br>Qualitative interviews/questionnaires and Health Economics questionnaires included and information updated<br>PAG information updated<br>Study monitoring procedures updated<br>Trial Steering Committee details updated<br>Data Monitoring Committee details updated<br>Secondary end point definitions added (Appendix 5)
3	5th August 2015	Change of measurement which defines “short cervix” and confers eligibility for randomisation	2	Change of measurement which defines a short cervix from ≤ 30mm to ≤ 35mm. The anticipated population centile (≤ 30th centile) is unchanged.
4	10th January 2018	Clarification of text, modification of the data collection and safety	5	• Clarification of Co-enrolment<br>• Modification of data collection to include hospitals, which are not participating STOPPIT2 sites<br>• Safety reporting guidance modified for clarity.<br>• Increase in the number of participants from 1850 to 2500 for cervical length scanning to facilitate randomisation which remains at 500.

For peer review only

STANDARD PROTOCOL ITEMS: RECOMMENDATIONS FOR INTERVENTIONAL TRIALS

SPIRIT 2013 Checklist: Recommended items to address in a clinical trial protocol and related documents*

Section/item	Item No	Description
**Administrative information**		
Title	1	Descriptive title identifying the study design, population, interventions, and, if applicable, trial acronym Page 1
Trial registration	2a	Trial identifier and registry name. If not yet registered, name of intended registry Page 4
	2b	All items from the World Health Organization Trial Registration Data Set Page 4
Protocol version	3	Date and version identifier Page 12
Funding	4	Sources and types of financial, material, and other support Page 13
Roles and responsibilities	5a	Names, affiliations, and roles of protocol contributors Page 1
	5b	Name and contact information for the trial sponsor Page 13
	5c	Role of study sponsor and funders, if any, in study design; collection, management, analysis, and interpretation of data; writing of the report; and the decision to submit the report for publication, including whether they will have ultimate authority over any of these activities Page 13
	5d	Composition, roles, and responsibilities of the coordinating centre, steering committee, endpoint adjudication committee, data management team, and other individuals or groups overseeing the trial, if applicable (see Item 21a for data monitoring committee) Appendix for DMC
**Introduction**		
Background and rationale	6a	Description of research question and justification for undertaking the trial, including summary of relevant studies (published and unpublished) examining benefits and harms for each intervention Page 3-4
	6b	Explanation for choice of comparators Page 3-4
Objectives	7	Specific objectives or hypotheses Page 6

For peer review only

Trial design	8	Description of trial design including type of trial (eg, parallel group, crossover, factorial, single group), allocation ratio, and framework (eg, superiority, equivalence, noninferiority, exploratory) Page 4

Methods: Participants, interventions, and outcomes

Study setting	9	Description of study settings (eg, community clinic, academic hospital) and list of countries where data will be collected. Reference to where list of study sites can be obtained Page 6
Eligibility criteria	10	Inclusion and exclusion criteria for participants. If applicable, eligibility criteria for study centres and individuals who will perform the interventions (eg, surgeons, psychotherapists) Page 5
Interventions	11a	Interventions for each group with sufficient detail to allow replication, including how and when they will be administered Page 6
	11b	Criteria for discontinuing or modifying allocated interventions for a given trial participant (eg, drug dose change in response to harms, participant request, or improving/worsening disease) Page 7
	11c	Strategies to improve adherence to intervention protocols, and any procedures for monitoring adherence (eg, drug tablet return, laboratory tests) Page 8
	11d	Relevant concomitant care and interventions that are permitted or prohibited during the trial Page 6
Outcomes	12	Primary, secondary, and other outcomes, including the specific measurement variable (eg, systolic blood pressure), analysis metric (eg, change from baseline, final value, time to event), method of aggregation (eg, median, proportion), and time point for each outcome. Explanation of the clinical relevance of chosen efficacy and harm outcomes is strongly recommended Page 6
Participant timeline	13	Time schedule of enrolment, interventions (including any run-ins and washouts), assessments, and visits for participants. A schematic diagram is highly recommended (see Figure) Page 8
Sample size	14	Estimated number of participants needed to achieve study objectives and how it was determined, including clinical and statistical assumptions supporting any sample size calculations Page 11
Recruitment	15	Strategies for achieving adequate participant enrolment to reach target sample size Page 6

Methods: Assignment of interventions (for controlled trials)

Allocation:

Sequence generation	16a	Method of generating the allocation sequence (eg, computer-generated random numbers), and list of any factors for stratification. To reduce predictability of a random sequence, details of any planned restriction (eg, blocking) should be provided in a separate document that is unavailable to those who enrol participants or assign interventions Page 4
Allocation concealment mechanism	16b	Mechanism of implementing the allocation sequence (eg, central telephone; sequentially numbered, opaque, sealed envelopes), describing any steps to conceal the sequence until interventions are assigned Page 5
Implementation	16c	Who will generate the allocation sequence, who will enrol participants, and who will assign participants to interventions Page 4, 5, 6
Blinding (masking)	17a	Who will be blinded after assignment to interventions (eg, trial participants, care providers, outcome assessors, data analysts), and how Page 5
	17b	If blinded, circumstances under which unblinding is permissible, and procedure for revealing a participant's allocated intervention during the trial n/a

Methods: Data collection, management, and analysis

Data collection methods	18a	Plans for assessment and collection of outcome, baseline, and other trial data, including any related processes to promote data quality (eg, duplicate measurements, training of assessors) and a description of study instruments (eg, questionnaires, laboratory tests) along with their reliability and validity, if known. Reference to where data collection forms can be found, if not in the protocol Page 6
	18b	Plans to promote participant retention and complete follow-up, including list of any outcome data to be collected for participants who discontinue or deviate from intervention protocols Page 8
Data management	19	Plans for data entry, coding, security, and storage, including any related processes to promote data quality (eg, double data entry; range checks for data values). Reference to where details of data management procedures can be found, if not in the protocol Page 9
Statistical methods	20a	Statistical methods for analysing primary and secondary outcomes. Reference to where other details of the statistical analysis plan can be found, if not in the protocol Page 9
	20b	Methods for any additional analyses (eg, subgroup and adjusted analyses) Page 10
	20c	Definition of analysis population relating to protocol non-adherence (eg, as randomised analysis), and any statistical methods to handle missing data (eg, multiple imputation) Page 9

Methods: Monitoring

Data monitoring	21a	Composition of data monitoring committee (DMC); summary of its role and reporting structure; statement of whether it is independent from the sponsor and competing interests; and reference to where further details about its charter can be found, if not in the protocol. Alternatively, an explanation of why a DMC is not needed Page 10
	21b	Description of any interim analyses and stopping guidelines, including who will have access to these interim results and make the final decision to terminate the trial Page 9, 10
Harms	22	Plans for collecting, assessing, reporting, and managing solicited and spontaneously reported adverse events and other unintended effects of trial interventions or trial conduct Page 6
Auditing	23	Frequency and procedures for auditing trial conduct, if any, and whether the process will be independent from investigators and the sponsor Page 10

Ethics and dissemination

Research ethics approval	24	Plans for seeking research ethics committee/institutional review board (REC/IRB) approval Page 12
Protocol amendments	25	Plans for communicating important protocol modifications (eg, changes to eligibility criteria, outcomes, analyses) to relevant parties (eg, investigators, REC/IRBs, trial participants, trial registries, journals, regulators) Page 12
Consent or assent	26a	Who will obtain informed consent or assent from potential trial participants or authorised surrogates, and how (see Item 32) Page 9
	26b	Additional consent provisions for collection and use of participant data and biological specimens in ancillary studies, if applicable n/a
Confidentiality	27	How personal information about potential and enrolled participants will be collected, shared, and maintained in order to protect confidentiality before, during, and after the trial Page 10
Declaration of interests	28	Financial and other competing interests for principal investigators for the overall trial and each study site Page 14
Access to data	29	Statement of who will have access to the final trial dataset, and disclosure of contractual agreements that limit such access for investigators Page 11
Ancillary and post-trial care	30	Provisions, if any, for ancillary and post-trial care, and for compensation to those who suffer harm from trial participation Page 8

For peer review only

Dissemination policy	31a	Plans for investigators and sponsor to communicate trial results to participants, healthcare professionals, the public, and other relevant groups (eg, via publication, reporting in results databases, or other data sharing arrangements), including any publication restrictions Page 12
	31b	Authorship eligibility guidelines and any intended use of professional writers Page 12
	31c	Plans, if any, for granting public access to the full protocol, participant-level dataset, and statistical code Page 12
**Appendices**		
Informed consent materials	32	Model consent form and other related documentation given to participants and authorised surrogates Appendix tbc
Biological specimens	33	Plans for collection, laboratory evaluation, and storage of biological specimens for genetic or molecular analysis in the current trial and for future use in ancillary studies, if applicable n/a

*It is strongly recommended that this checklist be read in conjunction with the SPIRIT 2013 Explanation & Elaboration for important clarification on the items. Amendments to the protocol should be tracked and dated. The SPIRIT checklist is copyrighted by the SPIRIT Group under the Creative Commons "Attribution-NonCommercial-NoDerivs 3.0 Unported" license.

For peer review only

BMJ Open

Protocol for an open randomised trial of the (Arabin) pessary to prevent preterm birth in twin pregnancy, with health economics and acceptability – STOPPIT-2.

Journal:	*BMJ Open*
Manuscript ID	bmjopen-2018-026430.R1
Article Type:	Protocol
Date Submitted by the Author:	28-Sep-2018
Complete List of Authors:	Norman, Jane; University of Edinburgh<br>Norrie, John; Edinburgh Clinical Trials Unit, University of Edinburgh No. 9, Bioquarter<br>Maclennan, Graeme; University of Aberdeen, Health Services Research Unit<br>Cooper, David; University of Aberdeen, Health Services Research Unit<br>Whyte, Sonia; The University of Edinburgh<br>CunninghamBurley, Sarah; University of Edinbrugh, Public Health Sciences<br>Smith, Joel; University of Oxford, Health Economics Research Centre, Nuffield Department of Population Health<br>Shennan, Andrew; Kings College London, Maternal and Fetal Research Unit<br>Robson, Stephen; NIHR CRN North East and North Cumbria<br>Thornton, Steven; Queen Mary University of London<br>Kilby, Mark; Birmingham Women's and Children's NHS Foundation Trust<br>Marlow, Neil; University College London, Institute of Child Health<br>Stock, Sarah; University of Edinburgh MRC Centre for Reproductive Health, Tommy's Centre for Maternal and Fetal Health; University of Western Australia School of Women's and Infant's Health,<br>Bennett, Philip; Imperial College London Department of Surgery and Cancer<br>Denton, Jane; Bryan Multiple Births Centre, Director of the Multiple Births Foundation
**Primary Subject Heading**:	Obstetrics and gynaecology
Secondary Subject Heading:	Paediatrics
Keywords:	preterm birth, cervical pessary, twin pregnancy, preterm labour

SCHOLARONE™
Manuscripts

Title: Protocol for an open randomised trial of the (Arabin) pessary to prevent preterm birth in twin pregnancy, with health economics and acceptability – STOPPIT-2.

Authors: Norman Jane E[1], Norrie John [2], MacLennan Graeme[3], Cooper David[3], Whyte Sonia[1], Cunningham Burley Sarah C[4], Smith Joel BE[5], Shennan Andrew[6], Robson Stephen C[7], Thornton Steven[8], Kilby Mark D[9], Marlow Neil [10], Stock Sarah[4], Bennett Philip R[11], Denton Jane[12]

Affiliations
1. Tommy's Centre for Maternal and Fetal Health, MRC Centre for Maternal and Fetal Health, University of Edinburgh, Edinburgh, UK
2. Edinburgh Clinical Trials Unit, University of Edinburgh, Edinburgh, UK
3. Centre for Healthcare Randomised Trials, University of Aberdeen, Aberdeen, UK
4. Usher Institute, University of Edinburgh, Edinburgh, UK
5. Health Economics Research Centre (HERC), Nuffield Department of Population Health, University of Oxford, Oxford, UK
6. Tommy's Centre for Maternal and Fetal Health, King's College London, London, UK
7. Institute of Cellular Medicine, University of Newcastle, Newcastle, UK
8. Barts and the London School of Medicine and Dentistry, Queen Mary University of London, London, UK
9. Fetal Medicine Centre, Birmingham Women's & Children's Foundation Trust and College of Medical and Dental Science, University of Birmingham, Birmingham, UK
10. Institute for Women's Health, University College London, London, UK
11. Imperial College London, London, UK
12. Multiple Births Foundation, London, UK

Corresponding author:
Professor Jane E Norman
Tommy's Centre for Maternal and Fetal Health,
MRC Centre for Maternal and Fetal Health
University of Edinburgh
The Queen's Medical Research Institute
47 Little France Crescent
Edinburgh
EH16 4TJ
Tel: +44 (0) 131 242 2694
Email: jane.norman@ed.ac.uk

Keywords: preterm birth, cervical pessary, twin pregnancy, preterm labour

Word count (excluding title page, abstract, references, figures, tables): 6010

For peer review only

Abstract:

Introduction

The STOPPIT-2 study aims to determine the clinical utility of the Arabin cervical in preventing preterm birth in women with a twin pregnancy and a short cervix, about which there is current uncertainty. STOPPIT-2 will resolve uncertainty around effectiveness for women with a twin pregnancy and a cervical length of 35mm or less, define adverse effects, ascertain acceptability and estimate NHS costs and savings.

Methods

STOPPIT-2 is a pragmatic multicentre open label randomised controlled trial. Consenting women with twin pregnancy will have an transvaginal ultrasound scan of their cervical length performed between 18+0 to 20+6 weeks gestation by an accredited practitioner: women with a cervical length of ≤ 35mm will be eligible for inclusion in the treatment phase of the study. The intervention by the insertion of the Arabin cervical pessary will be compared with standard treatment (no treatment).

The primary outcomes are (obstetric) spontaneous onset of labour for the mother leading to delivery before 34 weeks gestation and (neonatal) a composite of specific adverse outcomes or death occurring up to the end of the first four weeks after the estimated date of delivery to either or both babies.

We plan to recruit 500 women in the treatment phase of the study. Assuming a treatment effect of 0.6, and background rates of 35% and 18% respectively for each of the primary outcomes, our study has 85% power to detect a difference between the intervention and the control groups.

Analysis

Data will be analysed on the intention to treat principle.

Ethics

STOPPIT-2 was approved by the South East Scotland Ethics Committee 02 on 29th August 2014, reference number 14/SS/1031 IRAS ID 159610.

Dissemination

Peer reviewed journals, presentations at national and international scientific meetings.

Registration.

ISRCTN and NCT websites on 3/12/14 and 9/9/14, ref nos ISRCTN98835694, NCT02235181.

For peer review only

Strengths and limitations of the study

Strengths

- Randomised multicentre trial
- Cervical length measured by accredited individuals
- Prespecified cervical length threshold for randomisation
- Larger sample size than previously studied

Limitations

- Inability to blind women or caregivers to treatment allocation

Introduction

Preterm birth is common in twin pregnancy. The rate of twinnng itself is increasing. Twinning is associated with significant death or serious morbidity for the babies but there are no effective treatments. In 2010, 52.7% of multiple births were preterm and there is a clear health need for prevention of preterm birth in twins. This excess of prematurity (compared with singletons) leads to neonatal and infant mortality rates of 14.2 and 18.8 per 1,000 live births respectively (over fivefold that of singletons). Progesterone appears ineffective in preventing preterm birth in twins [1] and cerclage appears harmful [2]. Prematurity is thought to account for over 70% of twin neonatal deaths and adversely affects fetal survivors, with increased risks of future respiratory problems, motor and sensory impairment, learning difficulties, and social and behavioural difficulties.

The costs to the NHS of prematurity in twins are considerable. Twins alone account for over 20% of neonatal unit cot stays, a significant excess given they comprise only 2% of all births. Together, the complications of preterm birth result in an estimated annual cost of £2.946 billion to the public purse in England and Wales (2006 prices) [3]. Twins make a major contribution to this. There is a clear expressed need for innovative interventions to reduce preterm birth in both high and low income countries [4, 5]. The 2011 NICE Multiple Pregnancy Guideline Group noted that bed rest at home or in hospital, progesterone, cervical cerclage and oral tocolytics are all ineffective at preventing preterm birth in twins, concluding that alternative effective interventions are urgently required. The guideline predated the publication of studies on pessary to prevent preterm birth in twins, and there was no attempt to evaluate effectiveness. The Cochrane review on "cervical pessary for preventing preterm birth" [6] identified only one randomised trial (in singletons) which "showed the beneficial effect of the cervical pessary in preventing preterm birth", and called for randomised trials to be performed in multiple pregnancy [6].

Four randomised trials have been reported efficacy of cervical pessaries to prevent preterm birth in twins. A summary forest plot on the efficacy of the pessary to prevent preterm birth (using the entire population randomised each study) is shown in the Figure. The first trial to be published was the ProTwin study, which randomised 813 women with twin pregnancy to treatment with an Arabin pessary (inserted between 16 and 20 weeks gestation) or to standard treatment[7]. Although the pessary did not reduce preterm birth or the primary neonatal composite outcome overall, both of these events were significantly reduced in the prespecified subgroup of women with a cervical length less than the 25th centile (n=143) with a RR (95% CI) of preterm birth before 32 weeks of 0.49 (0.24-0.97) and RR (95%) of composite neonatal outcome of 0.42 (0.19 - 0.91). A subsequent randomised study of1,180 unselected women showed no difference in the rate of the primary outcome of spontaneous birth <34 weeks (13.6% vs. 12.9%; relative risk 1.054, 95% confidence interval [CI] 0.787-1.413; p=0.722) [8]. A preplanned subgroup analysis of 214 women with a cervical length of ≤ 25 mm also showed no benefit of the pessary (hazard ratio, 1.256; 95% CI, 0.760- 2.074; P =0.374). These data contrast with a smaller study published the same year, showing that, in women with a cervical length of ≤ 25 mm, spontaneous preterm birth was less common in the pessary group 11/68 (16..2%) compared with the expectant management group (26/66 (39.4%)[9]. Lastly, the POPT study of women with twin pregnancy and a cervical length </= 30 mm at 18 + 0 to 27 + 6 weeks' gestation randomised 46 women and found no difference in the primary outcome of preterm birth < 34 weeks (39% vs 35%; relative risk, 1.13 (95% CI, 0.53-2.40)[10].

Taken together, these data suggests that the cervical pessary does not work in unselected women, but may work in women with a short cervix; (at higher risk of preterm birth); consequently STOPPIT-2 will test the efficacy of the Arabin pessary in women with a short cervix. A cervical length threshold for recruitment of less than or equal to the 30th centile was chosen to maximise the number of women who could benefit. When planning the trial, it was thought that the 30th centile for cervical length would

For peer review only - http://bmjopen.bmj.com/site/about/guidelines.xhtml

equate to a cervix of less than or equal to 30mm in length, based on a large UK study [11]. However, a meta-analysis published in the early phases of the pilot for STOPPIT-2 [12], and data from the first 20 women in STOPPIT-2 itself, suggested that the 30th centile of cervical length in twins at 18-21 weeks gestation is 35mm. A cervical length threshold of less than or equal to 35mm (which we assume to be the 30th centile) was therefore used for eligibility. The quality control measures of the Fetal Medicine Foundation (http://www.fetalmedicine.com), or the Perinatal Quality Foundation (https://clear.perinatalquality.org), are employed to ensure validity of cervical length measurements.

Research priorities identified by NICE include randomised trials to determine "[effective interventions] in preventing spontaneous preterm birth in women with twin[s] .., especially in those at high risk of preterm birth". Additionally, the Preterm Birth Clinical Study Group of the RCOG identified "Efficacy of the Arabin pessary" amongst its top five research priorities. We are aware of several other studies in active or planned recruitment comparing a cervical pessary with standard care for prevention of preterm birth or its consequences in women with a preterm birth including NCT03418311 (planned n=672), NCT03058536 (planned n=312), ACTRN12616000875404 (planned n=140), NCT02708264 (planned n=242), NCT02350231 (planned n=100), and NCT01334489 (planned n=352).

After publication of STOPPIT-2 results, we plan to collaborate in subsequent individual patient data meta-analyses and update the economic decision model in light of new evidence. If the cervical pessary is effective in reducing preterm birth in twin pregnancy, it could make a major contribution to human health and ensure a more efficient allocation of resources in the UK NHS. However, we need to determine whether it is effective (and safe), since the evidence to date is not strong or robust enough to support its adoption as a first line intervention.

Methods

Design

STOPPIT-2 is a multicentre open label randomised controlled trial of the Arabin pessary (CE marked Device) versus standard treatment in women with twin pregnancy recruited from NHS antenatal clinics. The study is in two phases – a SCREENING phase, in which women with a short cervix (cervical length of ≤ 35mm) are identified, and a TREATMENT phase, in to which women with a short cervix will be randomised to either treatment with Arabin pessary or Standard treatment. An internal pilot will be performed to assess recruitment rates and to determine feasibility.

Objectives

Primary Objective

The primary objective of this study is to test the hypothesis that the Arabin cervical pessary reduces spontaneous preterm birth in women with a twin pregnancy and a short cervix (less than or equal to 35mm), reducing adverse neonatal outcomes and health care costs. We will also address the hypothesis that the pessary is acceptable to pregnant women. Subgroup analyses will determine effectiveness in women with a cervical length less than or equal to 25mm, and in women with a dichorionic pregnancy.

Secondary Objectives

The secondary objectives are to confirm (using the entire cohort of women undergoing cervical length scanning) the profile of cervical length measurements in women with twin pregnancy in the UK, and the positive and negative likelihood ratios of a variety of cervical length thresholds to determine spontaneous preterm birth before 34 weeks gestation.

Randomisation

Randomisation to Arabin pessary or standard treatment is performed by the central randomisation facility at the study data centre using a web portal. The allocation sequence will be generated by computer. Participants will be assigned a unique study identifier and staff will be required to enter minimal patient details prior to randomisation. Randomisation will be minimised with a random element rather than stratified given the properties of minimisation in ensuring comparability between the groups. Minimisation variables include booking hospital, cervical length and chorionicity (mono or dichorionic) but are not limited to these. The study is open (i.e. not masked), so that both the

For peer review only - http://bmjopen.bmj.com/site/about/guidelines.xhtml

participant and the Investigator will know which treatment has been allocated. The investigator will inform the woman of treatment allocation after randomisation.

Population

We will recruit pregnant women who meet the study eligibility criteria and who are booked for delivery at one of the participating sites. Case notes will be reviewed by the local clinical or research teams prior to recruitment. We anticipate that all eligible women expecting twins and who are attending for antenatal care in each of the sites will be invited to participate. Eligible women will normally be informed of the study at routine antenatal appointments. The following inclusion and exclusion criteria will apply.

Inclusion criteria (all must apply)

- Presenting with twin pregnancy (monochorionic or dichorionic).
- Gestation established by scan at ≤16 weeks according to NICE guidelines.
- Aged 16 years or older
- Wishing to participate in both the SCREENING and RANDOMISATION phase of the study
- Known chorionicity (as defined by first trimester ultrasound screening)

Exclusion criteria – screening phase (none must apply)

- Unable to give written informed consent
- Known significant congenital structural or chromosomal fetal anomaly at the time of inclusion
- Existing or planned cervical cerclage in the current pregnancy
- Existing or planned (prior to 20+6 weeks gestation) treatment for twin to twin transfusion syndrome in the current pregnancy
- Suspected or proven rupture of the fetal membranes at the time of recruitment
- Bulging fetal membranes at the time of recruitment
- Singleton pregnancy or higher order multiple pregnancies and/or women who have had any fetal death (i.e. fetal heart, previously detected) in the index pregnancy (prior to randomisation).
- Known sensitivity, contraindication or intolerance to silicone
- Involved in a clinical trial of an investigational medicinal product (CTIMP), phase 1 study or investigating a treatment for the prevention of pre-term birth
- Monochorionic monoamniotic pregnancy
- Heavy bleeding due to a low lying placenta prior to randomisation

Inclusion criteria – screening phase

- Cervical length ≤ 35mm at 18+0 – 20+6 weeks gestation confirmed by an accredited clinician

Exclusion criteria – treatment phase (none must apply)

- Cervical length > 35mm at 18+0 – 20+6 weeks gestation.
- Cervical length not measured at 18+0 – 20+6 weeks gestation.
- Bulging fetal membranes at the time of pessary insertion.
- Suspected or proven rupture of the fetal membranes at the time of pessary insertion.

To assess eligibility for the TREATMENT phase, all women who fulfil the inclusion criteria for the SCREENING phase will undergo ultrasound measurement of cervical length between 18+0 and 20+6 weeks gestation by an accredited operator (see later). If convenient, cervical length measurement may be done at the time of the routine fetal anomaly scan. In centres where cervical length measurements are part of routine clinical care women may be consented after the cervical length and will not be asked to have a further TV scan, provided:

- The transvaginal scan measurement was taken by an accredited operator (completed CLEAR [USA] or Fetal Medicine Foundation cervical length training) and is delegated by the PI
- The TV scan was conducted between 18+0 and 20+6 weeks gestation,
- The woman is eligible (as per the above inclusion/ exclusion criteria) and the pregnancy is not ≥ 21 weeks gestation.

Images of the cervical length measurements will be anonymised and uploaded to the eCRF.

For peer review only

Women with a cervical length of ≤ 35mm will continue to the TREATMENT phase, following verbal reconfirmation of intent to participate and randomised to either the intervention or the comparator group, described below.

Enrolment and adherence of participating centres
We will recruit from UK NHS and European hospitals. Sufficient centres will be involved to ensure that our planned sample size is achieved. Regular meetings will be held with clinicians and research staff from recruiting centres to maintain study enthusiasm and to achieve our planned sample size.

Intervention
The intervention is the Arabin pessary, inserted through the vagina and around the cervix between 18+0 and 20+6 weeks gestation, and given in addition to standard care. Video instructions for pessary insertion are available on the Arabin website[1]. There are no prohibited co-treatments. A trained member of the local research team will insert the pessary.

Comparator
The comparator is standard care. There are no prohibited co-treatments.

Outcomes (Women recruited to screening and treatment phase)
There are two primary outcomes, an obstetric and a neonatal primary outcome.

- Obstetric primary outcome: all births before 34+0 weeks following the spontaneous onset of labour. Preterm prelabour rupture of membranes < 34 weeks with or without contractions will also be included in this definition of spontaneous onset of labour [13]. Iatrogenic delivery due to maternal or fetal conditions will not be considered to fulfil the criteria for the primary outcome.
- Neonatal primary outcome: the primary neonatal outcome is a composite of adverse outcomes including stillbirth or neonatal death, periventricular leukomalacia, early respiratory morbidity (defined as any need for supplemental oxygen >30%, CPAP [continuous positive airway pressure] or intra-tracheal ventilation or surfactant replacement therapy within the first week of life), intraventricular haemorrhage, necrotising enterocolitis and proven sepsis all measured up to 28 days after the expected date of delivery.

Obstetric
Key outcomes

- Mean gestation at delivery
- Adverse events: infection, cervical trauma
- Incidence of all births before 37+0 weeks gestation
- Acceptability of the pessary as determined by participant questionnaire
- Experience of pessary removal

Other outcomes

- Incidence of all births before each of 28+0, 32+0, and 34+0 weeks gestation
- Preterm birth before 34 weeks gestation preceded by preterm premature membrane rupture
- Incidence of all births before 28, 32, 34 and 37 weeks preceded by the spontaneous onset of labour
- Method of delivery (in three categories: spontaneous vaginal delivery or vaginal breech, forceps or ventouse, caesarean section)
- Duration of labour overall, and of each of the first and second stages of labour
- Duration of stay in hospital
- Other adverse events including haemorrhage, tachycardia, vaginal injury, other trauma
- Serious maternal adverse events up to 28 days after discharge from the hospital

Key Neonatal secondary outcomes:

- Incidence of each of the individual components of the primary neonatal outcome
- Median weight (g) of the newborn at birth
- Death of live born babies within the first 28 days after birth
- Discrete episodes of bloodstream or CNS infection (positive blood or CSF culture)

---
[1] https://dr-arabin.de/produkt/arabin-cerclage-pessary-perforated/?lang=en

For peer review only

- Within first 72 hours
- Between 72 hours and discharge

Other neonatal outcomes

- Birth weight centile (for gestation) within 4 weeks after expected date of delivery
- Death of live born babies within the first 28 days after EDD
- Cord pH
- Apgar score at 1 and 5 minutes
- Need for resuscitation
- Need for surfactant administration
- Bronchopulmonary dysplasia
- Retinopathy of prematurity (stage 1 or more)
- Retinopathy of prematurity requiring surgery (laser or cryotherapy)
- Necrotising enterocolitis, (medical or surgical treatment of confirmed cases
- Twin to twin transfusion (Quintero stage 1 or more)
- Days of respiratory support [either mechanical ventilation or CPAP]
- Days of oxygen therapy
- Daily level of care.
- Seizures requiring therapy.
- Hyperbilirubinaemia requiring exchange transfusion.
- Periventricular leukomalacia / echogenicity

Other outcomes include:

- Acceptability and experience of the pessary as determined by participant questionnaire issued at 36 weeks gestation
- The joint distribution of the cumulative hospital costs and the primary clinical outcome.
- Cervical length measurements for all participants to determine the likelihood ratios for short cervix and spontaneous preterm birth.

We will also collect information on use of the following therapies to allow sensitivity analyses in future individual patient data meta-analyses

- Progestogens (IM or vaginal) after 16 weeks gestation
- Cervical suture post randomisation
- Tocolytics
- Steroids
- Magnesium sulphate
- Antibiotics
- Arabin pessary in the screened only women

Outcomes (women recruited to screening phase only).
For women recruited to the screening phase but are not randomised we will collect maternal and neonatal outcomes as described above. We will not collect information on co-treatments (other than those described above), adverse events, acceptability or costs.

Criteria for discontinuing intervention
Participation is voluntary. A woman has the right to discontinue or withdraw at any time for any reason. The Investigator also has the ability to discontinue a participant. The reason and circumstances will be documented in the electronic case report form. Unless a participant asks to completely withdraw from the study, those who discontinue treatment will be asked to continue to participate in study visits, to facilitate analysis under the intention to treat principle.

If treatment is suspended or stopped due to a serious adverse reaction, the local Principal Investigator will arrange for follow-up visits or telephone calls until the event has resolved or stabilised or until the study has ended, whichever is sooner. In order to avoid bias and/or missing adverse events, outcome data will be collected (including data from the patient record) and used in the analysis.

We will note whether participants have adhered to the treatment assignation. In the intervention arm, we will define adherence as women who have a pessary in situ at 28 weeks gestation (whether or not

For peer review only

it has been out and been reinserted during that time). Women who deliver before 28 weeks, will count as adherent if they had their pessary until 7 days before delivery. Those in the standard care arm will be considered adherent unless they had a pessary inserted.

Visits

Study visits will be conducted at approximately four weekly intervals (plus or minus one week) following randomisation until 36 weeks, when the pessary will be removed. The study visits will be carried out on those randomised to the pessary and those undergoing standard treatment. Study visits could be face to face or by phone or other means of communication, e.g. via letter or email. Pregnancy and fetal well-being will be reviewed at these visits. Additionally, we will collect the following outcomes:

- Any pregnancy complications (adverse events) including bleeding.
- Experience of the pessary during the previous 4 weeks (if applicable)
- Hospital admissions (Serious Adverse Events) – reasons and duration

Pessary removal

The pessary will be removed at 35^{+0} - 36^{+6} weeks gestation. The participant will be asked to complete a short questionnaire about her experience and (if appropriate) of having the pessary. For those allocated to pessary treatment, a vaginal examination will be conducted and the pessary removed. Information will be collected on the ease with which removal was achieved. If the pessary is removed before 35 weeks, we will record the indication.

Ancillary and post trial care

Care of participants in parallel to and beyond the trial will be provided within the UK National Health service. There will be no no-fault compensation for those who suffer harm from trial participation.

Qualitative data

A nested qualitative study involving pregnant women will explore their views and experiences of methods of recruitment and the consent process, their understanding and expectations of trial participation, including randomisation and the screening component, and experiences of the intervention. We will interview women who:

- Consented, were screened but found to have a long cervix (up to 10)
- Consented, were screened, have a short cervix and were randomised and allocated to the control group (up to 10)
- Consented, were screened, have a short cervix and were randomised and allocated to the treatment group (up to 20).

Up to 15 interviews will be conducted with a range of health care professionals involved in the trial in order to explore their views and experiences of the process.

Baseline characteristics (screening and treatment phase)

In addition to the outcomes, we will collect the following baseline characteristics on all women:
Age
Height
Weight
Cervical length
Current Smoking
Current alcohol

Obstetric History: Parity, Miscarriage
Medical conditions: Hypertension, Insulin dependent diabetes, Respiratory disease, Cardiac disease Neurological disease, Skin condition, Thrombophilia, Current Pregnancy
Fetal anomaly scan: Twin 1, Twin 2 (whether performed and the result)
Amniocentesis: Twin 1, Twin 2 (whether performed and the result)

Data collection forms are available on the STOPPIT-2 website at
https://w3.abdn.ac.uk/hsru/STOPPIT2/Public/DownloadPage.aspx

For peer review only

Consent

Written informed consent will be obtained from all participating women. Model information sheet and consent forms are available on the study website at https://w3.abdn.ac.uk/hsru/STOPPIT2/Public/DownloadPage.aspx. Women will be given information about the study by the investigator team at each study site, invited to consider participation and will be given adequate time (at least 24 hours) to read the information sheet. If the woman waives this opportunity but still wishes to participate, consent may be taken after a shorter time. Details of the consent process, including the name of the doctor confirming eligibility and the member of the study team taking consent will be recorded in the woman's notes and in the eCRF. Women undergoing ultrasound measurement of the cervix will be asked to complete a consent form prior to the procedure, which will cover both SCREENING and TREATMENT phases of the study. This will minimise inconvenience to the women (reducing the number of visits required to the hospital) and minimise the time undergoing vaginal examination. We will seek permission for future long term follow up of the women and babies via record linkage into national databases (health, social, and educational) although such follow up is beyond the scope of the study described here.

Data Analysis (SCREENING phase data)

We will use cervical length measurements at baseline screening, gestation at delivery, whether delivery was preceded by spontaneous onset of labour and / or preterm premature membrane rupture to determine positive and negative likelihood ratios for spontaneous preterm birth before 34 weeks for a variety of cervical length thresholds. Women treated with the pessary will be excluded from this analysis.

Data Analysis (TREATMENT phase data)

The statistical analysis will be according to the intention to treat principle; all participants will remain in their allocated group for analysis. Statistical significance will be at the 5% level with corresponding 95% confidence intervals (CI) derived. Randomised groups will be described at baseline and follow-up using mean (standard deviation), median (inter-quartile range) and counts (with percentages) where appropriate.

For the primary obstetric and neonatal outcomes, logistic regression with a fixed effect for the minimisation covariate chorionicity and a random effect for centre will be used to obtain the odds ratios of the treatment effect, along with the 95% confidence interval and associated p-value. In the case of the composite neonatal outcome the components will also be reported individually to show which elements are driving the primary outcome. In the case of skewed recruitment resulting in small centres a regional effect will be considered in place of a centre effect.

Continuous secondary outcomes will be analysed using linear regression, adjusting for chorionicity. Binary categorical secondary outcomes will be analysed using logistic regression as per the primary outcomes.

Secondary outcomes with more than two categories will be analysed using multinomial logistic regression.

Analysis of fetal outcomes will allow for clustering within twins by fitting mother as a random effect in a mixed effects logistic regression model.

We will undertake predefined subgroup analyses of the primary outcome by monochorionicity, cervical length (≤ 25mm and ≤ 28mm. In the subgroup analyses, statistical significance will be at the 1% level with corresponding 99% confidence intervals (CI).

It is not anticipated that primary outcome data will be missing. If data are missing, this is likely to be due to a miscarriage, stillbirth, neonatal or maternal death and therefore it will not be imputed.

A post-hoc comparison will also be performed between participants who had membrane rupture and those who did not.

A per protocol analysis (including those in the intervention arm who are adherent, and excluding those in the control arm who have a pessary inserted) will also be performed.

For peer review only

No formal interim analyses are planned.

The statistical analysis plan is included as Appendix 1 to this paper.

Data management

The primary source of data arise from medical notes. These data are entered directly from source, by study site staff, into the eCRF (electronic case report form). The eCRF has been developed in accordance with CHaRT software development standards at the University of Aberdeen. Data entry is carried out remotely by authorised study personnel. Accuracy of entry and the storage of source documentation is the responsibility site study personnel. Validation checks will be set up and run in real time on the eCRF. Checks will include missing data, out of range values, illogical entries and invalid responses. Users will be informed of data issues when the eCRF page is saved providing users with the opportunity to correct the data immediately, where possible. The eCRF system will generate a missing data query for all items not completed at the time. Cross form checks comparing data items across different forms will be created, where possible or written as reports.

Data Analysis (Health economic data)

The economic component will consider the joint distribution of the cumulative hospital costs and the primary clinical outcome from the perspective of the UK NHS.NHS. Generalised linear models will be used to estimate the costs between the two treatment groups. The cost estimates and primary clinical outcome will be combined within a cost-effectiveness analysis to calculate the incremental cost per preterm birth prevented. The length of stay distributions will be valued using a per diem unit cost derived from NHS reference costs[13]. Monetary values will be attached to the labour costs for healthcare practitioners associated with each treatment pathway using standard NHS pay and price estimates[14, 15]. No discounting of hospital costs and clinical outcomes will be undertaken for the within-trial economic component. Flexible parametric regression models will be used to explore the dependency between cost and the primary clinical outcome as well as heterogeneous treatment effects within a sensitivity analysis to inform a decision-analytic model. AA decision model will be developed to estimate the longer term resource consequences and health outcomes of preterm birth following twin pregnancy. Model parameter uncertainty will be addressed using probabilistic sensitivity analysis summarised using the cost-effectiveness acceptability curve at differing willingness to pay thresholds..

Data Analysis (Qualitative research)

The nested qualitative interview study will be analysed using an inductive cross-sectional thematic approach described by Mason[16]. This approach involves applying indexing coding to the entire data set providing the means to identify patterns and themes in the data from each arm of the study. The use of qualitative software will also help to facilitate a collaborative approach to the analysis thereby ensuring consistency and rigour[17]. This method has been chosen as it will help to identify how women experience all aspects of the study in the context of their current pregnancy that builds up a descriptive and conceptual interpretation of the findings that maintains the integrity of each research participant's accounts. The same analytical approach will be taken for the data generated through interviews with health care professionals.

Study monitoring

On site, trial monitoring will not be conducted on behalf of the Co-Sponsors. Remote monitoring to verify eligibility, consent, staff training and data entry quality will be performed. The research involves the use of a CE marked device used for its intended purpose. Audits may be carried out by individual site R&D departments as per the Research Governance Framework, these audits are independent of the sponsors.

A trial steering committee (TSC) and a data monitoring committee (DMC) will be appointed. The membership and charter for each is included in Appendices 2 and 3. The committees will meet not less than annually, or more often if issue arise. There are no formal stopping rules: the TSC and DMC charters state that "The TSC, based on recommendations from the DMC, may recommend early termination of the trial or modification of the study design in the event of a clear accumulating data or on the basis of information available from other sources or on safety grounds". "Recommendations to amend the protocol or conduct of the study made by the DMC will be considered and either accepted

For peer review only

or rejected by the TSC. The TSC will be responsible for deciding whether to continue or to stop the trial based on the DMC recommendations".

Confidentiality of data

All records such as laboratory specimens, questionnaires and evaluation forms, will be identified in a manner designed to maintain participant confidentiality. All records will be kept in a secure storage area with limited access. Collection of participant identifiers is performed only to ensure compliance with governance procedures (eg confirmation of informed consent). Paper records with identifying information will be kept in a locked cabinet with limited access. Electronic identifying information will be kept in a separate trial database and will not be revealed as part of routine data analysis or in any data sharing exercise. Clinical information will not be released without the written permission of the participant. The Investigator and study site staff involved with this study will not disclose or use for any purpose other than performance of the study, any data, record, or other unpublished, confidential information disclosed to those individuals for the purpose of the study. Prior written agreement from the sponsor or its designee must be obtained for the disclosure of any said confidential information to other parties.

Data protection

All Investigators and study site staff involved with this study must comply with the requirements of the Data Protection Act 1998 and subsequent General Data Protection Regulations 2018 with regard to the collection, storage, processing and disclosure of personal information and will uphold the Act's core principles. Access to collated participant data will be restricted to those clinicians treating the participants, representatives of the sponsor(s) and representatives of regulatory authorities. Computers used to collate the data will have limited access measures via user names and passwords.

Published results will not contain any personal data that could allow identification of individual participants.

Sample size calculation

We plan to recruit 2500 women to the SCREENING phase and randomise 500 women in the TREATMENT phase. We anticipate that around 58 sites (largely UK NHS sites) will be involved.

Women with a short cervix are at higher risk of preterm birth and may benefit most from pessary treatment. We aim to treat women with a cervical length less than or equal to 35mm, which we believe to be around the 30th centile. Assuming this to be correct, a sample size of 1850 screened was initially identified to generate 555 eligible for randomisation in the TREATMENT PHASE. However, following re-analysis of data (masked to treatment allocation) in September 2017 after 29 months of screening (with data on 1214 women screened), we estimated that to randomise 550 women, the sample size of screening needed to increase to 2500. Although we will encourage women to participate in the screening phase only if they wish also to participate in the treatment phase, we have allowed for a further 10% drop out after screening, so that we now aim to randomise 500 women in the treatment phase.

We plan to randomise 500 women (250 in each group). We assume a relative risk of the primary obstetric outcome (spontaneous preterm labour leading to preterm birth before 34 weeks) in the pessary group of 0.6. We believe our relative risk reduction is conservative, given a relative risk of 0.49 for delivery before 32 weeks[7] and 0.47 for delivery before 34 weeks (21% v 42%) (Liem S, personal communication) in the ProTwin study. We anticipate that 35% of women in the control group will deliver before 34 weeks. We believe that this is a conservative estimate, given that a systematic review indicated that 34.9% of women with a cervical length of 35mm or less (when scanned at 20 weeks gestation) will deliver preterm before 32 weeks[12]. Assuming a baseline rate of 35% and a relative risk of 0.6, a sample size of 500 has 94% power to detect a difference at the 5% significance level. If the preterm birth rate before 34 weeks is only 30%, the power drops to 88%. Both allow for losses to follow up and imperfect compliance.
For the primary neonatal outcome, Liem showed an effect size of 0.42 at the child level, and an incidence of 24% in the control group[7]. We have powered our study for a relative risk of 0.6 for the primary neonatal outcome. Assuming prevalence rates as in Liem, our study would have 97% power.

For peer review only

In practice, our post screening groups of women with cervical length less than or equal to 35mm is probably a lower risk than in the Liem group (less than 38mm), given comparisons of the rate of preterm delivery in each of the control groups. Hence if we assume a lower rate of the neonatal primary outcome of (say) 18% we still have 88.4% power to detect a relative risk of 0.6 in the Arabin pessary group. Such a calculation assumes analysis at the child level is appropriate for the neonatal outcome.

For the subgroup of women with cervical length of less than or equal to 25mm, the anticipated rate of the primary obstetric outcome in the control group is 82/159 (51%) [11]. The study has 85% power to detect a relative risk of 0.6 in this group, with a sample size of 234 (25mm was the 14th centile in the To et al).

Patient and public involvement.
The importance of the research question was identified by two major groups, both involving PPI. The first was the NICE guideline on Multiple Pregnancy, published in 2011, which had embedded PPI The guideline generated the following research recommendation: "What interventions are effective in preventing spontaneous preterm birth in women with twin and triplet pregnancies, especially in those at high risk of preterm birth? The second group was the Clinical Study Group from the RCOG, which has extensive lay and patient involvement. "The use of the Arabin pessary for preventing preterm birth" is one of five research priorities identified in late 2012.

There was PPI involvement in the development of the protocol at grant submission stage, including in choice of the outcome measures. Specifically, the CEO of a charity focussing on preterm birth (Jane Denton, Multiple Births Foundation) was a co-applicants on this project was and involved in the study design.

Patients and the public are also involved in the trial steering committee for this study, which oversaw the conduct of the study: both through participation of individual patients and through participation of Keith Read from Tamba (The Twins and Multiple Births Association) and two individual patients. Although PPI were not directly involved in individual patient recruitment, they provided helpful comments on the patient information sheet used for this purpose. PPI were members of the ethics committee who assessed the research burden associated with participating in this study. Individual study participants will be sent a summary of study findings, when the main study is published. We also make use of our connections with the charities Tommy's, the Multiple Births Foundation and the Twins and Multiple Births Association to disseminate information.

Ethics
STOPPIT-2 was approved by the South East of Scotland Research Ethics Committee 2 on 29th August 2014, reference number 14/SS/1031.

Dissemination
A study report will be prepared in accordance with GCP guidelines, and submitted to the funder (NIHR) for publication in the NIHR journals library. A summary report will be submitted for publication in a peer review journal. Data will be used for publication and presentation at scientific meetings. Authorship guidelines are attached at Appendix 4.

Access to data
The Chief Investigator (JEN) and all study statisticians (JN, GMacL and DC) will have full access to the final dataset data during analysis. Access will be provided to any other author on request. There are contractual limitations on disclosures.

Data deposition
We aim to deposit the data in a shared depository. We do not anticipate providing access to the statistical analysis code.

Protocol amendments
Version 1 of the STOPPIT 2 protocol dated 22nd July 2014 was approved by the ethics committee on 29th August 2014. A list of protocol amendments is included in an Appendix 5. It is not anticipated that

For peer review only

any further substantive amendments will be made, other than addition or closure of sites. Protocol amendments are notified to, and approved by the sponsor, funder and research ethics committee, and communicated to investigators after approval. The most recent approved version of the protocol is version 4, dated 10th January 2018. One final amendment of the protocol will be submitted to address minor updates made during the preparation of this protocol paper.

References

1. Norman JE, Mackenzie F, Owen P, Mactier H, Hanretty K, Cooper S, Calder A, Mires G, Danielian P, Sturgiss S, MacLennan G, Tydeman G, Thornton S, Martin B, Thornton JG, Neilson JP, Norrie J. Progesterone for the prevention of preterm birth in twin pregnancy (STOPPIT): a randomised, double-blind, placebo-controlled study and meta-analysis. Lancet. 2009 Jun 13;373(9680):2034-40.
2. Berghella V, Odibo AO, To MS, Rust OA, Althuisius SM. Cerclage for short cervix on ultrasonography: meta-analysis of trials using individual patient-level data. Obstetrics and gynecology. 2005 Jul;106(1):181-9.
3. Mangham LJ, Petrou S, Doyle LW, Draper ES, Marlow N. The cost of preterm birth throughout childhood in England and wales. Pediatrics. 2009 Feb;123(2):e312-27.
4. Chang HH, Larson J, Blencowe H, Spong CY, Howson CP, Cairns-Smith S, Lackritz EM, Lee SK, Mason E, Serazin AC, Walani S, Simpson JL, Lawn JE. Preventing preterm births: analysis of trends and potential reductions with interventions in 39 countries with very high human development index. Lancet. 2012 Nov 15.
5. Norman JE, Shennan AH. Prevention of preterm birth-why can't we do any better? Lancet. 2012 Nov 15.
6. Abdel-Aleem H, Shaaban OM, Abdel-Aleem MA. Cervical pessary for preventing preterm birth. Cochrane database of systematic reviews (Online). 2013;5:CD007873.
7. Liem S, Schuit E, Hegeman M, Bais J, de Boer K, Bloemenkamp K, Brons J, Duvekot H, Bijvank BN, Franssen M, Gaugler I, de Graaf I, Oudijk M, Papatsonis D, Pernet P, Porath M, Scheepers L, Sikkema M, Sporken J, Visser H, van Wijngaarden W, Woiski M, van Pampus M, Mol BW, Bekedam D. Cervical pessaries for prevention of preterm birth in women with a multiple pregnancy (ProTWIN): a multicentre, open-label randomised controlled trial. Lancet. 2013 Oct 19;382(9901):1341-9.
8. Nicolaides KH, Syngelaki A, Poon LC, de Paco Matallana C, Plasencia W, Molina FS, Picciarelli G, Tul N, Celik E, Lau TK, Conturso R. Cervical pessary placement for prevention of preterm birth in unselected twin pregnancies: a randomized controlled trial. American journal of obstetrics and gynecology. 2016 Jan;214(1):3 e1-9.
9. Goya M, de la Calle M, Pratcorona L, Merced C, Rodo C, Munoz B, Juan M, Serrano A, Llurba E, Higueras T, Carreras E, Cabero L, Group PE-TT. Cervical pessary to prevent preterm birth in women with twin gestation and sonographic short cervix: a multicenter randomized controlled trial (PECEP-Twins). American journal of obstetrics and gynecology. 2016 Feb;214(2):145-52.
10. Berghella V, Dugoff L, Ludmir J. Prevention of preterm birth with pessary in twins (PoPPT): a randomized controlled trial. Ultrasound Obstet Gynecol. 2017 May;49(5):567-72.
11. To MS, Fonseca EB, Molina FS, Cacho AM, Nicolaides KH. Maternal characteristics and cervical length in the prediction of spontaneous early preterm delivery in twins. American journal of obstetrics and gynecology. 2006 May;194(5):1360-5.
12. Kindinger LM, Poon LC, Cacciatore S, MacIntyre DA, Fox NS, Schuit E, Mol BW, Liem S, Lim AC, Serra V, Perales A, Hermans F, Darzi A, Bennett P, Nicolaides KH, Teoh TG. The effect of gestational age and cervical length measurements in the prediction of spontaneous preterm birth in twin pregnancies: an individual patient level meta-analysis. BJOG. 2016 May;123(6):877-84.
13. Villar J, Papageorghiou AT, Knight HE, Gravett MG, Iams J, Waller SA, Kramer M, Culhane JF, Barros FC, Conde-Agudelo A, Bhutta ZA, Goldenberg RL. The preterm birth syndrome: a prototype phenotypic classification. American journal of obstetrics and gynecology. 2012 Feb;206(2):119-23.
14. Curtis L. Costs of Health and Social Care Personal Social Services Research Unit, University of Kent 2013.
15. 2011-2012. Department of Health NHS reference costs. 2011-2012 NHS Reference Costs 2013.
16. Mason J. Qualitative researching. London: Sage Publications Ltd; 2018.
17. Silverman D. Doing qualitative research: a practical handbook. London: Sage publications Ltd; 2000.

For peer review only - http://bmjopen.bmj.com/site/about/guidelines.xhtml

Author's contributions
All authors (JEN, JN, GMacL, DC, SW, SCB, JBES, AS, SCR, ST, MDK, NM, SS, PRB, and JD) contributed to the intellectual content of this protocol. JEN and SW wrote the first draft, with the exception of the statistical analysis plan (which was written by DC, with input from GMacL and JN), health economics (written by JBES) and the qualitative research (written by SCB). All authors (JEN, JN, GMacL, DC, SW, SCB, JBES, AS, SCR, ST, MDK, NM, SS, PRB, and JD) contributed to further drafting and approved the final version.

Funding statement
This study was funded by the NIHR Health Technology Assessment Programme (project number 13/04/22).

Sponsor
The trial co-sponsors are University of Edinburgh and NHS Lothian, ACCORD, The Queen's Medical Research Institute, 47 Little France Crescent, Edinburgh, EH16 4TJ

Neither the study sponsor not the funder has or will have any role, responsibility or authority for study design, collection, management, analysis and interpretation of data, writing or the report, or decision to submit the report for publication.

Department of Health and Social Care disclaimer
The views expressed are those of the author(s) and not necessarily those of the NHS, the NIHR or the Department of Health and Social Care

Competing interests statement
The authors declare the following competing interests:
Jane Norman is a member of the HTA Maternal, Neonatal and Child health prioritisation panel. She has performed consultancy for the pharma companies LifeArc and Dilafor outside the scope of this work.

ST provides commercial consultancy advice and is a Trustee or advisor for funding organisations.

AS is on the HTA commissioning board and has received funding from Hologic (paid to institution) for preterm prediction studies (outside the scope of this work.)

Acknowledgements
We thank Katie Morris and Fiona Mackie, both of the University of Birmingham, for provision of the Figure, and the patient advisors who helped us with the study.

For peer review only

Appendix 1.

Statistical analysis plan

For peer review only

Appendix 2.

Trial steering committee charter and membership.

For peer review only

Appendix 3

Data monitoring committee charter and membership.

For peer review only

Appendix 4
Authorship guidelines

For peer review only

Figure

Forest plot

For peer review only

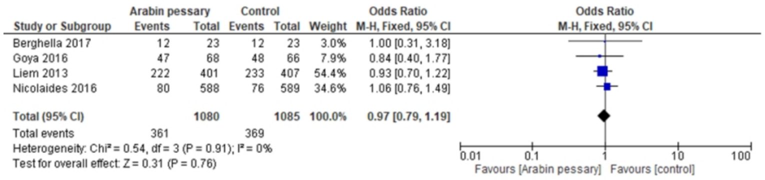

99x139mm (300 x 300 DPI)

STOPPIT-2

An open randomised trial of the Arabin pessary to prevent preterm birth in twin pregnancy, with health economics and acceptability – STOPPIT-2.

Statistical Analysis Plan
Treatment Phase
Version 1.0

Prepared by:
David Cooper
(CHaRT Statistician)
Signed: David J. Cooper
Date: 30.8.2018

Approved by:
Professor Jane Norman
(Chief Investigator)
Signed: [signature]
Date: 30th August 2018

Protocol version 5 23/8/2018

Contents

1 Study Objectives
1.1 Primary objective
1.2 Secondary objective

2 Study Design
2.1 Randomisation and blinding

3 Outcome Measures
3.1 Primary outcome
3.2 Secondary outcomes
3.3 Timing of outcome measurements
3.4 Adverse events

4. Sample Size and Power Calculations

5. Statistical Methods
5.1 Primary outcome
5.2 Secondary outcomes
5.3 Subgroup analyses
5.4 Missing data

6. Dummy Tables

7. References

For peer review only

List of Tables

1. Baseline clinical characteristics
2. Experience of device fitting
3. Experience of device through trial
4. Experience of device at removal
5. Primary obstetric outcome of birth before 34+0 weeks – overall and by subgroup of chorionicity and cervical length
6. Secondary obstetric outcomes – gestational age at delivery, duration of labour and duration of stay in hospital
7. Secondary obstetric outcomes – method of delivery, incidence of births before 28+0, 32+0, 34+0 and 37+0 weeks, adverse events
8. Primary composite neonatal outcome and incidence of individual components
9. Secondary neonatal outcomes – continuous outcomes
10. Secondary neonatal outcomes – categorical outcomes
11. Safety issues

For peer review only

1. Study Objectives

1.1 Primary objective

The primary objective of this study is to test if the Arabin cervical pessary reduces the risk of spontaneous preterm labour leading to preterm birth in women with a cervical length less than or equal to 35mm, who are expecting twins.

1.2 Secondary objectives

The secondary objectives of this study are to use the entire cohort of women undergoing cervical length scanning to identify the profile of cervical length measurement in women with twin pregnancy in the UK. The study also aims to obtain the positive and negative likelihood ratios of spontaneous preterm birth before 34 weeks gestation for a variety of cervical length thresholds. A separate plan will be created to describe this analysis.

2. Study Design

STOPPIT-2 is a multicentre, open label, randomised controlled trial of the Arabin pessary versus standard treatment (no additional treatment other than what would normally be received) in women with twin pregnancy.

The study has two phases:-

A *screening* phase in which women with a short cervix (cervical length of ≤ 35mm) will be identified;

A *treatment* phase in which women with a short cervix will be randomised to treatment with Arabin pessary or standard treatment.

Women will be seen in the antenatal clinic setting.

2.1 Randomisation and blinding

Randomisation will be carried out between 18 weeks + 0 days gestation and 20 weeks + 6 days gestation. Women with a short cervix (≤ 35mm) who agree to participate in the treatment phase of the study will be randomised to either treatment with the Arabin cervical pessary or to standard care (no additional treatment). Randomisation will be performed by the central randomisation facility based within CHaRT.

Randomisation will be minimised with a random element rather than stratified given the properties of minimisation in ensuring comparability between the groups. The minimisation variables are centre and chorionicity (monochorionic or dichorionic).

Neither the participant nor the investigator are blinded to which treatment has been allocated. The investigator will inform the woman of treatment allocation after randomisation.

3. Outcome Measures

3.1 Primary outcomes

- *Obstetric*: all births before 34 weeks + 0 days gestation following the spontaneous onset of labour. Pre-term prelabour rupture of membranes before 34 weeks with or without contractions is also included in the definition of spontaneous onset of labour.
- *Neonatal*: a composite of adverse outcomes including any death from trial entry to 28 days post expected date of delivery (miscarriage, stillbirth, neonatal or postneonatal death), periventricular

For peer review only

leukomalacia, early respiratory morbidity (defined as any need for supplemental oxygen >30%, CPAP [continuous positive airway pressure], or intra-tracheal ventilation or surfactant replacement therapy within the first week of life), intraventricular haemorrhage, necrotizing enterocolitis and proven sepsis. All these will be measured up to 28 days after the expected date of delivery determined by the first trimester ultrasound.

3.2 Secondary outcomes

Obstetric:

Key outcomes

- Mean gestation at delivery
- Adverse events: infection, cervical trauma
- Incidence of all births before 37+0 weeks gestation
- Acceptability of the pessary as determined by participant questionnaire
- Experience of pessary removal

Other outcomes

- Incidence of all births before each of 28+0, 32+0, and 34+0 weeks gestation
- Preterm birth before 34+0 weeks gestation preceded by preterm premature membrane rupture
- Incidence of all births before each of 28+0, 32+0, 34+0 and 37+0 weeks gestation preceded by spontaneous onset of labour
- Method of delivery (in three categories: spontaneous vaginal delivery or vaginal breech, forceps or ventouse, caesarean section)
- Duration of labour overall, and of each of the first and second stages of labour
- Duration of stay in hospital
- Other adverse events including haemorrhage, tachycardia, vaginal injury, other trauma
- Serious maternal adverse events up to 28 days after discharge from the hospital

Key *Neonatal secondary outcomes:*

- Incidence of each of the individual components of the primary neonatal outcome
- Median weight (g) of the newborn at birth
- Death of live born babies within the first 28 days after birth
- Discrete episodes of bloodstream or CNS infection (positive blood or CSF culture)
  - Within first 72 hours
  - Between 72 hours and discharge

Other neonatal outcomes

- Birth weight centile (for gestation) within 4 weeks after expected date of delivery
- Death of live born babies within the first 28 days after EDD
- Cord pH
- Apgar score at 1 and 5 minutes
- Need for resuscitation
- Need for surfactant administration

For peer review only

  - Bronchopulmonary dysplasia
  - Retinopathy of prematurity (stage 1 or more)
    - Retinopathy of prematurity requiring surgery (laser or cryotherapy)
  - Necrotising enterocolitis, (medical or surgical treatment of confirmed cases
  - Twin to twin transfusion (Quintero stage 1 or more)
- Days of respiratory support [either mechanical ventilation or CPAP]
- Days of oxygen therapy
- Daily level of care.
- Seizures requiring therapy.
- Hyperbilirubinaemia requiring exchange transfusion.
- Periventricular leukomalacia / echogenicity

4. Sample Size and Power Calculations

We plan to recruit 2500 women to the SCREENING phase and randomise 500 women in the TREATMENT phase. We anticipate that around 58 sites (largely UK NHS sites) will be involved.

We aim to treat women with a cervical length less than or equal to 35mm, which we believe to be around the 30th centile. Assuming this to be correct, a sample size of 1850 women screened was initially identified as the number of women required, to generate 555 women eligible for randomisation in the TREATMENT PHASE. However, re-analysis of data (masked to treatment allocation) in September 2017 after 29 months of screening (with data on 1214 women screened), we estimated that to randomise 550 women, the sample size of screening needed to increase to 2500. Although we will encourage women to participate in the screening phase only if they wish also to participate in the treatment phase, we have allowed for a further 10% drop out after screening, so that we aim to randomize 500 women in the treatment phase.

We plan to randomise 500 women of 500 (250 in each group). We assume a relative risk of the primary obstetric outcome (spontaneous preterm labour leading to preterm birth before 34 weeks) in the Arabin pessary group of 0.6. We believe our relative risk reduction is conservative, given a relative risk of 0.49 for delivery before 32 weeks [20] and 0.47 for delivery before 34 weeks (21% v 42%)(Liem S, personal communication) in the ProTwin study. We anticipate that 35% of women in the control group will deliver before 34 weeks. Again we believe that this is a conservative estimate, given that a systematic review indicated that 34.9% of women with a cervical length of 35mm or less (when scanned at 20 weeks gestation) will deliver preterm before 32 weeks (Kindinger LM et al 2015). Assuming a baseline rate of 35% and a relative risk of 0.6, a sample size of 500 has 94% power to detect a difference at the 5% significance level between the groups. If the preterm birth rate before 34 weeks is only 30%, power drops to 88%. Both allow for losses to follow up and imperfect compliance.
For the primary neonatal outcome, Liem showed an effect size of 0.42 at the child level, and an incidence of 24% in the control group. We have powered our study for a relative risk of 0.6 for the primary neonatal outcome. Assuming prevalence rates as in Liem, our study would have 97% power. In practice, our post screening groups of women with cervical length less than or equal to 35mm group is probably at somewhat lower risk than the Liem less than 38mm group (given comparisons of the rate of preterm delivery in each of the control groups). Hence if we assume a lower rate of the neonatal primary outcome of (say) 18% we still have 88.4% power to detect a relative risk of 0.6 in the Arabin pessary group. Such a calculation assumes analysis at the child level is appropriate for the neonatal outcome.

For the subgroup of women with cervical length of less than or equal to 25mm, the anticipated rate of the primary obstetric outcome in the control group is 82/159 (51%) [10]. The study has 85% power to detect a

For peer review only

relative risk of 0.6 in this group, with a sample size of 234 (25mm was the 14th centile in the To et al) 0.48 x sample size in < 30mm group).

5. Statistical Methods

The primary statistical analysis will be according to the intention to treat principle in which all participants will remain in their allocated group for analysis. An additional per protocol analysis will also be performed.

Statistical significance will be at the 5% level with corresponding 95% confidence intervals (CI) derived.

Randomised groups will be described at baseline and follow-up using means (with standard deviations), medians (with inter-quartile ranges) and counts (with percentages) where appropriate.

5.1 Primary outcomes

For the primary obstetric and neonatal outcomes, logistic regression with a fixed effect for the minimisation covariate chorionicity and a random effect for centre will be used to obtain the odds ratios of the treatment effect, along with the 95% confidence interval and associated p-value. In the case of the composite neonatal outcome the components will also be reported individually to show which elements are driving the primary outcome. In the case of skewed recruitment resulting in small centres a regional effect will be considered in place of a centre effect.

5.2 Secondary outcomes

Continuous secondary outcomes will be analysed using linear regression, adjusting for chorionicity. Binary categorical secondary outcomes will be analysed using logistic regression as per the primary outcomes.

Secondary outcomes with more than two categories will be analysed using multinomial logistic regression.

Analysis of fetal outcomes will allow for clustering within twins by fitting mother as a random effect in a mixed effects logistic regression model.

5.3 Subgroup analyses

Predefined subgroup analyses of the primary outcome by monochorionicity (no or yes), by cervical length (≤ 25mm) and also by cervical length ≤ 28mm will be undertaken.

A post-hoc comparison will also be performed between participants who had membrane rupture and those who did not.

In the subgroup analyses statistical significance will be at the 1% level with corresponding 99% confidence intervals (CI).

5.4 Missing Data

It is not anticipated that primary outcome data will be missing and if it is this is likely due to a miscarriage, stillbirth, neonatal or maternal death and therefore it will not be imputed.

For peer review only

6. Dummy Tables

Table 1: Baseline characteristics

Characteristic	Level	Arabin pessary N =	Standard treatment N =
Age	Mean (sd)		
	Min-Max		
Height	Mean(sd)		
	Min-Max		
Weight	Mean (sd)		
	Min-Max		
Cervical length	Mean (sd)		
	Min-Max		
Current Smoking	Yes, n (%)		
Current alcohol	Yes, n (%)		
Obstetric History			
Parity	>0, n (%)		
Miscarriage	>0, n (%)		
Medical conditions	>0, n (%)		
Hypertension	Yes, n (%)		
Insulin dependent diabetes	Yes, n (%)		
Respiratory disease	Yes, n (%)		
Cardiac disease	Yes, n (%)		
Neurological disease	Yes, n (%)		
Skin condition	Yes, n (%)		
Thrombophilia	Yes, n (%)		
Current Pregnancy			
Fetal anomaly scan: Twin 1	Normal – n (%)		
	Defined abnormality – n (%)		
	Uncertain abnormality – n (%)		
	Not done – n (%)		
Fetal anomaly scan: Twin 2	Normal – n (%)		
	Defined abnormality – n (%)		
	Uncertain abnormality – n (%)		
	Not done – n (%)		
Amniocentesis: Twin 1	Abnormal – n (%)		
Amniocentesis: Twin 2	Abnormal – n (%)		

For peer review only

Table 2: Experience of device fitting

**Arabin pessary**<br>**N =**	**At fitting**
Maternal experience of having device fitted – n (%)	
Painless	
Slightly uncomfortable	
Uncomfortable	
Very uncomfortable	
Worst pain imaginable	
Too painful to allow insertion	
Clinical team experience of fitting device – n (%)	
Easy	
Moderately easy	
Neither easy nor difficult	
Difficult	
Very difficult	
Impossible	

Table 3: Experience of device through trial

Arabin pessary<br>N =	36 week questionnaire
I could feel the pessary – n(%)	
Never	
A few times	
At least once every week	
Every day	
All the time	
I found the pessary uncomfortable – n(%)	
Never	
A few times	
At least once every week	
Every day	
All the time	
I found the pessary painful – n(%)	
Never	
A few times	
At least once every week	
Every day	
All the time	
I had vaginal discharge – n(%)	
Never	
A few times	
At least once every week	

For peer review only

Every day	
All the time	
I had vaginal bleeding – n(%)	
Never	
A few times	
At least once every week	
Every day	
All the time	
I had to use panty liners/sanitary towels because of the vaginal discharge/bleeding– n(%)	
Never	
A few times	
At least once every week	
Every day	
All the time	
The vaginal discharge was – n(%)	
The same as before the pessary	
A little more than before the pessary	
A lot more than before the pessary	
The vaginal bleeding was – n(%)	
The same as before the pessary	
A little more than before the pessary	
A lot more than before the pessary	
The vaginal discharge was – n(%)	
The same as in my last pregnancy	
A little more than in my last pregnancy	
A lot more than in my last pregnancy	
This is my first pregnancy	
The vaginal bleeding was – n(%)	
The same as in my last pregnancy	
A little more than in my last pregnancy	
A lot more than in my last pregnancy	
This is my first pregnancy	
Any other side effects experienced with the pessary	

Table 4: Experience of device removal

	**At removal**
Maternal experience of having device removed– n (%)	
Painless	
Slightly uncomfortable	
Uncomfortable	
Very uncomfortable	

For peer review only

Worst pain imaginable	
Clinical team experience of removing device – n (%)	
Easy	
Moderately easy	
Neither easy nor difficult	
Difficult	
Very difficult	

Table 5: Primary obstetric outcome of birth before 34+0 weeks – overall and by subgroup of chorionicity and cervical length

**Outcome**	**Arabin pessary N =**	**Standard treatment N =**	**Odds ratio (95% CI)**	**p-value**
All pregnancies				
Proportion of women delivering before 34 weeks – n (%)				*
Monochorionic pregnancy and cervical length ≤ 25mm				
Proportion of women delivering before 34 weeks – n (%)				
Dichorionic pregnancy and cervical length ≤ 25mm				
Proportion of women delivering before 34 weeks – n (%)				
Monochorionic pregnancy and cervical length ≤ 28mm				
Proportion of women delivering before 34 weeks – n (%)				
Dichorionic pregnancy and cervical length ≤ 28mm				
Proportion of women delivering before 34 weeks – n (%)				

* refers to p-value for proportion in Arabin pessary versus standard treatment group from logistic regression analysis adjusting for chorionicity

For peer review only

Table 6: Secondary obstetric outcomes – gestational age at delivery, duration of labour and duration of stay in hospital

**Outcome – mean (sd)**	**Arabin pessary N =**	**Standard treatment N =**	**Mean difference (99% CI)**	**p-value ***
Gestational age at delivery (weeks)				
Duration of labour stage 1 (mins)				
Duration of labour stage 2 (mins)				
Duration of labour overall (mins)				
Duration of hospital stay (days)				

* refers to p-value for proportion in Arabin pessary versus standard treatment group from linear regression analysis adjusting for chorionicity

For peer review only

Table 7: Secondary obstetric outcomes – method of delivery, incidence of births before 28+0, 32+0, 34+0 and 37+0 weeks, adverse events

**Outcome – n (%)**	**Arabin pessary N =**	**Standard treatment N =**	**Odds ratios (99% CI)**	**p-value ***
Method of delivery				
Spontaneous vaginal delivery				
Vaginal breech				
Forceps or ventouse				
Caesarian section				
Incidence of all births				
Before 28+0 weeks				
Before 32+0 weeks				
Before 34+0 weeks				
Before 37+0 weeks				
Incidence of births – preceded by spontaneous onset of labour				
Before 28+0 weeks				
Before 32+0 weeks				
Before 34+0 weeks				
Before 37+0 weeks				
Incidence of birth before 34+0 weeks preceded by PPMR				
Adverse events				
Infection				
Haemorrhage				
Tachycardia				
Significant maternal adverse event up to 28 days after discharge from hospital				

PPMR – preterm premature membrane rupture
* refers to p-value for proportion in Arabin pessary versus standard treatment group from logistic regression analysis adjusting for chorionicity or using proportional odds analysis

For peer review only

Table 8: Primary composite neonatal outcome and incidence of individual components

**Outcome – n (%)**	**Arabin pessary N =**	**Standard treatment N =**	**Odds ratio (95% CI)**	**p-value**
Composite neonatal outcome				
Individual components				
Stillbirth or neonatal death				
Periventricular leukomalacia				
Early respiratory morbidity				
Intraventricular haemorrhage				
Necrotizing enterocolitis				
Proven sepsis				

Data refer to all twins with the 95% CI and p-values adjusted for clustering within twins

For peer review only

Table 9: Secondary neonatal outcomes – continuous outcomes

**Outcome – mean (sd)**	**Arabin pessary N =**	**Standard treatment N =**	**Mean difference (99% CI)**	**p-value**
Birth weight centile within 4 weeks after expected d.o.d.				
Weight of newborn at birth				
Cord pH				
Agpar score at 1 minute				
Agpar score at 5 minutes				
Days of oxygen therapy				
Level of care days				

d.o.d. – date of delivery
Data refer to all twins with the 99% CI and p-values adjusted for clustering within twins

Table 10: Secondary neonatal outcomes – categorical outcomes

**Outcome – n (%)**	**Arabin pessary N =**	**Standard treatment N =**	**Odds ratio (99% CI)**	**p-value**
Need for resuscitation				
Fetal or neonatal death within the first 28 days after birth				
Need for surfactant administration				
Bronchopulmonary dysplasia				
Necrotising enterocolitis				
Discrete episodes of bloodstream or CNS infection				
Daily level of care				
Rate of major adverse neonatal outcomes before discharge from hospital				

Data refer to all twins with the 99% CI and p-values adjusted for clustering within twins

For peer review only

Table 11: Safety Issues – p-value from Fisher's Exact test on subjects

**Type**	**Arabin pessary N =**	**Standard treatment N =**	**p-value from exact test**
Mother died			
Intrauterine death			
Neonatal death			
Involved or prolonged inpatient maternal hospitalisation			
Involved persistent/significant maternal disability/incapacity			
Life threatening			
Chorioamnionitis or intrauterine infection			
Congenital anomaly/birth defect			

For peer review only

7. REFERENCES

1. To, M. S., E. B. Fonseca, F. S. Molina, A. M. Cacho and K. H. Nicolaides (2006). "Maternal characteristics and cervical length in the prediction of spontaneous early preterm delivery in twins." American journal of obstetrics and gynecology 194(5): 1360-1365.

2. Liem S et al (2013). "Cervical pessaries for prevention of preterm birth in women with a multiple pregnancy (ProTWIN): a multicentre, open-label randomised controlled trial". The Lancet 382(9901): 1341-1349.

3. Norman, J. E., F. Mackenzie, P. Owen, H. Mactier, K. Hanretty, S. Cooper, A. Calder, G. Mires, P. Danielian, S. Sturgiss, G. MacLennan, G. Tydeman, S. Thornton, B. Martin, J. G. Thornton, J. P. Neilson and J. Norrie (2009). "Progesterone for the prevention of preterm birth in twin pregnancy (STOPPIT): a randomised, double-blind, placebo-controlled study and meta-analysis." Lancet 373(9680): 2034-2040.

4. Kindinger LM, Poon LC, Cacciatore S, MacIntyre DA, Fox NS, Schuit E, Mol B, Liem S, Lim A, Serra V, Perales A, Hermans F, Darzi A, Bennett P, Nicolaides KH, Teoh TG (2015). The prediction of preterm birth in twin pregnancy: an individual patient level meta-analysis. BJOG in press.

For peer review only

STOPPIT-2 study

Trial Steering Committee Charter

Version 2.0 Date 22nd July 2015

**Study Title:**	An open randomised trial of the Arabin pessary to prevent preterm birth in twin pregnancy, with health economics and acceptability – STOPPIT-2.
**Chief Investigator:**	**Professor Jane Norman**
**REC Number:**	**14/SS/031**
**Sponsor:**	University of Edinburgh & NHS Lothian

For peer review only

Approval Signatures:

The following individuals, by providing their signatures, indicate their understanding of and willingness to comply with the roles and responsibilities assigned to them in this Charter.

1. TSC independent Chair:

________________	________________	_ _ / _ _ /_ _ _ _
PRINT NAME	SIGNATURE	DATE

2. TSC independent Member:

________________	________________	_ _ / _ _ /_ _ _ _
PRINT NAME	SIGNATURE	DATE

3. TSC independent Member:

________________	________________	_ _ / _ _ /_ _ _ _
PRINT NAME	SIGNATURE	DATE

4. Chief Investigator:

________________	________________	_ _ / _ _ /_ _ _ _
PRINT NAME	SIGNATURE	DATE

5. Trial Statistician:

________________	________________	_ _ / _ _ /_ _ _ _
PRINT NAME	SIGNATURE	DATE

For peer review only

CONTENTS

CONTENTS.. 1
1. Introduction 3
2. Membership of the TSC.. 3
2.1. Members:.. 4
2.2. Agreements 6
2.3. Indemnity.. 6
2.4. Payments.. 6
3. Responsibilities 6
4. Interaction between TSC and other study committees.. 8
5. Scheduling, Quorum, and Organization of Meetings.. 8
5.1. Attendance at meetings 9
5.2. Non-attendance at meetings.. 9
5.3. Report for the TSC.. 9
5.4. Contents of the TSC Report.. 10
6. Decision making 10
6.1. Recommendations.. 10
6.2. TSC records.. 11
7. Trial closure.. 11
9. Publication.. 12
10. References: 12

For peer review only

Abbreviations and glossary

CI	Chief Investigator
DMC	Data Monitoring Committee
eCRF	Electronic case report form
GCP	Good clinical practice
ISRCTN	International standard randomised controlled trial number
TSC	Trial Steering Committee
PMG	Project Management Group

For peer review only

For peer review only - http://bmjopen.bmj.com/site/about/guidelines.xhtml

1. Introduction

The role of the Trial Steering Committee (TSC) is to act as the oversight body for this trial on behalf of the Sponsors and the Trial Funder and to ensure that the trial is conducted according to the guidelines for Good Clinical Practice (GCP), Research Governance Framework for Health and Social Care and all relevant regulations and local policies.

The background to this trial and the trial objectives are described in the protocol. The purpose of this document is to describe the membership, terms of reference, roles, responsibilities, authority, decision-making and relationships of the Trial Steering Committee (TSC) for this trial, including the timing of meetings, methods of providing information to and from the TSC, frequency and format of meetings and relationships with other trial committees. The charter also describes the procedures for ensuring confidentiality and proper communication to and from the TSC and an outline of the content of the reports to be provided to the TSC.

2. Membership of the TSC

The STOPPIT-2 Trial TSC is an independent multidisciplinary group that, collectively, have experience/expertise in the management of patients with condition(s) relevant to study and anticipated adverse effects and in the conduct and monitoring of randomised clinical trials. The role of the TSC is to provide oversight for the trial and provide advice through its independent Chair to the Project Management Group (PMG) on all aspects of the trial.

For peer review only

For peer review only - http://bmjopen.bmj.com/site/about/guidelines.xhtml

2.1. Members

(Names redacted from this version)

INDEPENDENT (VOTING) MEMBERS

TSC Independent Chair
Neonatologist

TSC Independent Member
Obstetrician

TSC Independent Member
Patient Advisory Group (PAG) Representative

TSC Independent Member
Statistician

TSC Independent Member:
Charity lead

TSC Independent Member
Patient Advisory Group (PAG) Representative

For peer review only

NON-INDEPENDENT MEMBERS

Representative of the Sponsor (Non-Voting)

Chief Investigator
Professor Jane Norman

The Queen's Medical Research Institute
47 Little France Crescent
Edinburgh
EH16 4TJ
Tel: 0131 242 2694

Email:jane.norman@ed.ac.uk;

The Facilitator (Non-Voting)

Members of the Project Management Group (PMG) who may be invited to attend as non-voting members:

Trial Manager

Trial Statistician

NB: Other members of the Project Management Group (PMG) are invited at the discretion of the TSC Chair.

FUNDERS CONTACT:

Programme manager

For peer review only

2.2. Agreements

TSC independent members will be asked to formally acknowledge their agreement to join the group by signing the TSC charter indicating that they:

(1) agree to be a member of the TSC

(2) agree with the contents of this Charter

(3) and have declared any potential conflicts of interest.

TSC members should not have any apparent financial, scientific or intellectual conflict of interest (see tables 1 and 2) that could prevent them from objectively reviewing the study protocol, interim and final data and giving advice to the PMG. TSC members should disclose to the Chair any other conflicts they consider relevant. Any members who develop significant conflicts of interest during the course of the trial should inform the Chair and may need to consider resigning from the TSC.

2.3. Indemnity

The University of Edinburgh insurance indemnifies TSC members for their work on the committee.

2.4. Payments

Standard travel and subsistence costs only will be paid to members of the TSC. Representatives of the Patient Advisory Group (PAG) may receive payments for their time in attending meetings of the TSC as appropriate to their role as a member of the PAG. No other payments or rewards will be given to members.

3. Responsibilities

The TSC members, on behalf of the Sponsor and Funder, will have overall responsibility for the conduct of the trial and for safeguarding the rights, safety and wellbeing of participants. The Chair has previous experience of serving on trial committees and experience of Chairing meetings, and will facilitate and summarise discussions. Responsibilities of the TSC include:

- Reviewing selection/recruitment/retention of participants and their management.
- Finalising and reviewing study protocol and other study documentation.
- Determine if amendments to the protocol or changes to study conduct are required and deciding on changes to these and to study conduct in general. Any changes to trial documentation or conduct must be notified to the TSC.

For peer review only

- Reviewing adherence to the protocol by Investigators and participants.
- Assessing the impact and relevance of external evidence.
- Assessing integrity and completeness of data collected.
- Monitoring the overall conduct of the trial, ensuring that it follows the standards set out in the guidelines of GCP, assessing the safety and efficacy of the interventions, recruitment figures and completion of trial assessments.
- Reviewing, commenting and making decisions on extension requests.
- Reviewing the recommendations of the DMC (Data Monitoring Committee) (if applicable) and/or other study committees and suggesting appropriate action to the PMG.
- Monitoring the progress of study/trial and deciding on appropriate action in order to maximise the chances of completing it within the agreed timelines.
- Considering new information relevant to the study e.g. results from other studies that may have a bearing to the conduct of the study and deciding on appropriate action.

Additional responsibilities may include:

1. Endorsing the annual report to the funder and ethics (if required).
2. Approving proposed protocol amendments or new trial sub-studies.
3. Approving requests for early release of (subsets of) data.
4. Approving external applications for the use of stored samples (if applicable).
5. Approving presentation of results during the trial or soon after closure.
6. Approving strategies to improve recruitment or follow-up.

For peer review only

4. Interaction between TSC and other study committees

The responsibilities of each trial committee are outlined in the protocol and in the respective Charters. The relationships between these groups are summarised in Figure 1 below.

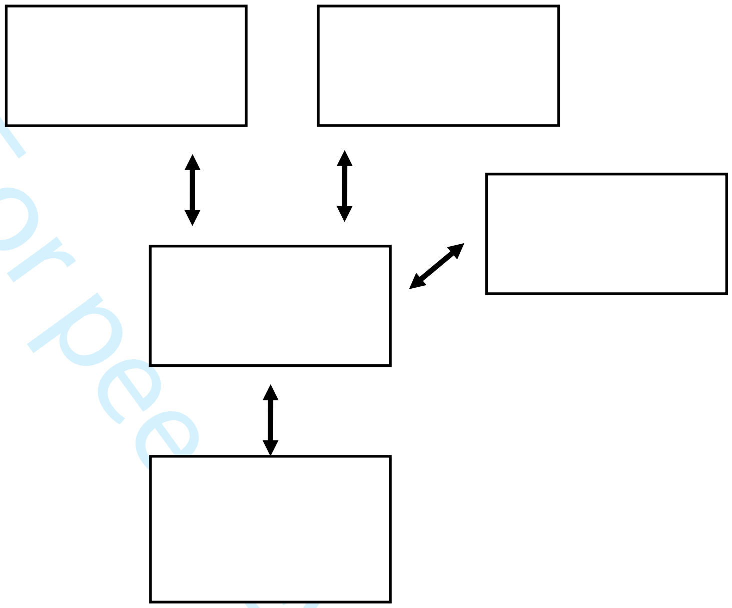

5. Scheduling, Quorum, and Organization of Meetings

The responsibility for calling and organising TSC meetings lies with the Chief Investigator in association with the Chair. The Chair, assisted by the Chief Investigator, is responsible for facilitating the meetings and summarising discussions. The Facilitator will be responsible for the organisation of meetings and should be copied into all communications with and between the TSC.

A meeting of the TSC is considered quorate if at least four independent members, including the Chair (unless otherwise agreed), take part, plus a member of the trial team.

The TSC will aim to meet in person at least yearly. The TSC should be available to provide independent advice as required, not just when meetings are scheduled. Major trial issues may need to be dealt with between meetings, by phone or by email. The TSC should maintain confidentiality of all information it receives. Members should not discuss confidential issues from their involvement in the study until the primary results have been published.

For peer review only - http://bmjopen.bmj.com/site/about/guidelines.xhtml

5.1. Attendance at meetings

Meetings will usually be limited to the TSC members (Section 2), observers from the sponsors, invited PMG members and the Facilitator. Other attendees may be invited for all or part of the meeting by the TSC. The observers are not members of the TSC but may be invited to provide expert input; other observers will be at the discretion of the TSC and the Facilitator but may include members of the PMG other than the CI.

Effort will be made to ensure that all members can attend. The Facilitator will work for a date that enables this. The CI must try to attend all meetings, especially if major actions are expected. Members who cannot attend in person should be encouraged to participate by teleconference. TSC members who will not be able to attend the meeting may pass comments to the TSC Chair via the Facilitator for consideration during the discussions. If, at short notice, any TSC members cannot attend then the TSC may still meet if at least four independent members, including the Chair (unless otherwise agreed), will be present, plus also a member of the trial team. If the TSC is considering a major action after such a meeting the TSC Chair should communicate with the absent members, including the CI, as soon after the meeting as possible to check they agree. If they do not, a further teleconference should be arranged with the full TSC.

5.2. Non-attendance at meetings

If an independent member does not attend a meeting or provide comments when requested between meetings, it will be ensured that the independent member is available for the next meeting. If an independent member does not attend the next meeting or provide comments when next requested, they will be asked if they wish to remain part of the TSC. If an independent member does not attend a third meeting, strong consideration will be given to replacing this member.

The TSC membership is for the duration of the trial. If any members leave the TSC, the PMG and TSC should provide recommendations for replacements promptly.

5.3. Report for the TSC

A report will be prepared by the trial manager on behalf of the PMG containing accrual and any matters affecting the trial. Additionally, the material may include a report *from* the DMC, requests *from* the PMG or draft publications. No trial outcome measure data will be presented by arm unless explicitly authorised by the DMC. Where relevant, accrual, compliance with follow-up and adherence to treatment may be presented by centre. The TSC will receive the report at least 1 week but preferably 2 weeks before any meetings. Different procedures may apply to teleconference meetings.

For peer review only - http://bmjopen.bmj.com/site/about/guidelines.xhtml

TSC members are expected to store securely copies of the reports to and from the TSC, agenda and minutes, as well as copies of communications between meetings. All documentation should be considered confidential.

5.4. Contents of the TSC Report

An outline example of the contents of the TSC report is given below:

- Major protocol amendments
- Patient screening
- Eligibility violations
- Protocol deviations/violations by investigators or participants
- Study accrual by month/total
- Completeness and quality of data collected/eCRF return
- Quality controls
- Safety reporting
- Any matters affecting the trial
- Compliance by patients to clinic visit
- Latest DMC recommendations

6. Decision making

All potential independent TSC members will have the opportunity to comment on the protocol as early as possible. Before recruitment begins the trial will have undergone review by the Sponsor/Funder (e.g. peer review for public sector trials), scrutiny by other trial committees and a research ethics committee. Therefore, if a potential independent TSC member has major reservations about the trial (e.g. the protocol, the logistics, ethical concerns) they should report these to the Chief Investigator and may decide not to accept the invitation to join. TSC members will be constructively critical of the ongoing trial, but also supportive of aims and methods of the trial.

6.1. Recommendations

On consideration of the information presented at these meetings, the TSC should provide recommendations of appropriate action in writing to the PMG who will be responsible for implementing any actions. The role of the Chair is to summarise discussions and encourage consensus. Therefore it is best for the chair to give his/ her own opinion last. It is important that the implications (ethical, statistical, practical and financial) for the trial be considered before any decision is made. Decisions and recommendations by the TSC should be unanimous, if not a vote may be taken.

The TSC should inform the PMG if:

For peer review only - http://bmjopen.bmj.com/site/about/guidelines.xhtml

For peer review only

- There are concerns about the safety of participants, recruitment, the integrity of trial data or adherence to the protocol.
- The TSC may also provide feedback to the DMC and where appropriate to the Sponsor/Funder.

The DMC may make recommendations to the TSC based on the interim data. Identification and circulation of published external evidence (e.g. from other trials/ systematic reviews) is not the responsibility of the TSC members; it is a responsibility of the PMG. However, the TSC should continue to be made aware of other data that may impact on the trial.

The TSC is the oversight body for the trial. However, the TSC should have good reason(s) before deciding not to accept requests from the PMG and recommendations from the DMC. If there are serious problems or concerns with the TSC decision following a DMC recommendation, a joint meeting of the TSC and DMC should be held. The information to be shown would depend upon the action proposed and each committee's concerns. Depending on the reason for the disagreement confidential data may have to be revealed to all or some of those attending such a meeting: this would be minimised where possible. The meeting will ideally be Chaired by an external expert who is not directly involved with the trial.

6.2. TSC records

The Minutes of the meeting including key points and actions will be prepared by the facilitator. The Facilitator will keep a central record of all minutes, reports and correspondence by the TSC in the Trial Master File. These minutes will describe the proceedings and include the recommendations of the TSC. All members of the TSC must agree the minutes and these will be signed off by the TSC Chair on behalf of all members. Minutes will be circulated to all TSC members, the PMG, the Sponsor and, if appropriate, the Trial Funder. Approved Minutes will also be filed in the Trial Master File.

7. Trial closure

The TSC, based on recommendations from the DMC, may recommend early termination of the trial or modification of the study design in the event of a clear accumulating data or on the basis of information available from other sources or on safety grounds. Possible decisions include:-

1. No action needed, trial continues as planned.

For peer review only

For peer review only - http://bmjopen.bmj.com/site/about/guidelines.xhtml

2. Early stopping due, for example, to overwhelming evidence benefit or evidence of harm of a treatment or external evidence (this should generally involve a recommendation from the DMC to unblind the TSC to these data).
3. Modifying target recruitment, or pre-analysis follow-up, based on any change to the assumptions underlying the original trial sample size calculation (but not on any emerging differences).
4. Sanctioning and/or proposing protocol changes.

9. Publication

Manuscripts that arise from the trial will be shared with the TSC and members will be able to comment. The TSC members and their affiliations will be acknowledged in reports of the trial.

10. References

The charter was developed using:

1. MRC Clinical Trials Unit template TSC Charter version 1.02, 13-Mar-2006
2. CCTU/GD018 Version No.1 Approved: 21/03/2013 http://www.cuh.org.uk/sites/default/files/research/CCTU_GD018%20Trial%20Steering%20Committee%20or%20Study%20Steering%20Committee%20Guidance.pdf accessed 24/04/2014
3. MRC Guidelines for GCP in clinical trials (1998) issued 01/Sep/2006 http://www.mrc.ac.uk/Utilities/Documentrecord/index.htm?d=MRC002416 accessed 24/04/2014
4. MHRA GCP Guide (2012)
5. Conroy et al.: Trial steering committees for randomised controlled trials: updating and redeveloping guidance and terms of reference informed by current practice and experience. Trials 2013 14 (Suppl 1):P128.

For peer review only

Table 1: Potential competing interests for independent members

- Stock ownership in any commercial companies involved
- Stock transaction in any commercial company involved (if previously holding stock)
- Consulting arrangements with the Sponsor/Funder
- Frequent speaking engagements on behalf of the intervention
- Career tied up in a product or technique assessed by trial
- Hands-on participation in the trial
- Involvement in the running of the trial
- Emotional involvement in the trial
- Intellectual conflict e.g. strong prior belief in the trial’s experimental arm
- Involvement in regulatory issues relevant to the trial procedures
- Involvement in the writing up of the main trial results in the form of authorship

Table 2: Potential competing interests for non-independent members

- Stock ownership in any commercial companies involved
- Stock transaction in any commercial company involved (if previously holding stock)
- Consulting arrangements with the Sponsor/Funder
- Frequent speaking engagements on behalf of the intervention
- Intellectual conflict e.g. strong prior belief in the trial’s experimental arm

For peer review only

Academic and Clinical Central Office for Research and Development

STOPPIT-2

Data Monitoring Committee Charter

**Study Title:**	An open randomised trial of the Arabin pessary to prevent preterm birth in twin pregnancy, with health economics and acceptability – STOPPIT-2.
**Chief Investigator:**	**Professor Jane Norman**
**REC number:**	**14/SS/031**
**Sponsor:**	University of Edinburgh & NHS Lothian

For peer review only

For peer review only - http://bmjopen.bmj.com/site/about/guidelines.xhtml

Approval Signatures:

The following individuals, by providing their signatures, indicate their understanding of and willingness to comply with the roles and responsibilities assigned to them in this Charter.

1. DMC Chair:

____________________	____________________	_ _/ _ _/_ _ _ _
PRINT NAME	SIGNATURE	DATE

2. DMC Member:

____________________	____________________	_ _/ _ _/_ _ _ _
PRINT NAME	SIGNATURE	DATE

3. DMC Member:

____________________	____________________	_ _/ _ _/_ _ _ _
PRINT NAME	SIGNATURE	DATE

4. Chief Investigator:

____________________	____________________	_ _/ _ _/_ _ _ _
PRINT NAME	SIGNATURE	DATE

5. [Trial/Unblinded] Statistician/Analyst:

____________________	____________________	_ _/ _ _/_ _ _ _
PRINT NAME	SIGNATURE	DATE

For peer review only

For peer review only - http://bmjopen.bmj.com/site/about/guidelines.xhtml

Table of Contents

1. Introduction 4
2. Primary responsibilities of the DMC 4
3. Membership of the DMC 4
3.1 Members 4
3.2 Conflicts of interest 5
4. Timing and purpose of the DMC meetings 5
4.1 Organisational meeting and early safety/trial integrity reviews 5
4.2 DMC meetings 6
5. Procedures to ensure confidentiality & proper communication 6
5.1 Closed sessions 6
5.2 Open sessions 7
5.3 Open and Closed reports 7
5.4 Minutes of the DMC meeting 7
5.5 Recommendations to the Trial Steering Committee 8
6. Statistical monitoring guidelines 8
7. Content of the DMC's open and closed reports 9
7.1 Open statistical report: outline 9
7.2 Closed statistical report: outline 9
8. DMC Payments 10
9. References 10

For peer review only

For peer review only - http://bmjopen.bmj.com/site/about/guidelines.xhtml

1. Introduction

This Charter is for the Data Monitoring Committee (DMC) for the STOPPIT-2 An open randomised trial of the Arabin pessary to prevent preterm birth in twin pregnancy, with health economics and acceptability. This study aims to determine whether the Arabin cervical pessary prevents preterm birth in women with a twin pregnancy and a short cervix. Women with a twin pregnancy are at high risk of preterm labour. The extra risk of preterm labour in twin pregnancy is the main reason why twin babies are five times more likely to die, both within the first month or the first year of life. Preterm babies who survive are at increased risk of long term disability, and often require extensive and costly health care, so if preterm birth could be prevented in twins this would be extremely beneficial.

The Charter will define the primary responsibilities of the DMC, its membership, and the purpose and timing of its meetings. The Charter will also provide the procedures for ensuring confidentiality and proper communication, the statistical monitoring guidelines to be implemented by the DMC, and an outline of the content of the Open and Closed Reports that will be provided to the DMC.

2. Primary responsibilities of the DMC

The DMC will be responsible for safeguarding the interests of trial participants, assessing the safety of the interventions during the trial, reviewing external evidence with an impact on risk/benefit balance and for monitoring the overall conduct of the clinical trial. The DMC will provide recommendations about safety, modifying or continuing the trial to the Trial Steering Committee (TSC). To contribute to enhancing the integrity of the trial, the DMC may also formulate recommendations relating to the selection, recruitment, or retention of participants, or their management, or to improving their adherence to protocol-specified regimens and retention of participants, and the procedures for data management and quality control.

The DMC will be advisory to the TSC. The TSC will be responsible for promptly reviewing the DMC recommendations, to decide whether to continue or terminate the trial, and to determine whether amendments to the protocol or changes in study conduct are required.

3. Membership of the DMC

3.1 Members

The DMC is an independent multidisciplinary group consisting of clinicians, statisticians and methodologists that, collectively, have experience/expertise in the management of patients with the condition relevant to study and anticipated adverse effects and in the conduct and monitoring of

For peer review only - http://bmjopen.bmj.com/site/about/guidelines.xhtml

randomised clinical trials. University of Edinburgh insurance indemnifies DMC members for their work on the committee.

DMC: Chair (Independent statistician)

DMC: Independent Clinical Investigator (Obstetrician)

DMC: Independent Clinical Investigator (Neonatologist)

DMC: (non-Independent) statistician/analyst (TBC)

(Note – names removed in this version to be published)

3.2 Conflicts of interest

Independent DMC membership is restricted to individuals free of relevant significant conflicts of interest. The source of these conflicts may be financial, scientific or regulatory in nature. Individuals who fulfil any of the following criteria are automatically disqualified from membership: the study investigators, individuals with conflicts of interest as determined by the Chair of the DMC.

The DMC members will disclose to fellow members any consulting agreements or financial interests they have with Edinburgh University, NHS or the manufacturing license holder of an investigational medicinal product/medical device subject to investigation during the study. The DMC will be responsible for deciding whether these consulting agreements or financial interests materially impact their objectivity.

The DMC members will be responsible for advising fellow members of any changes in these consulting agreements and financial interests that occur during the course of the trial. Any DMC member who thinks they may have developed a relevant significant conflict of interest during the course of the trial must declare this. If the other DMC members consider it to be a relevant significant conflict of interest, the member should resign from the DMC.

DMC membership is normally for the duration of the clinical trial. If any member leaves the DMC during the course of the trial, the sponsor, in consultation with the TSC and/or investigators will promptly appoint their replacement.

4. Timing and purpose of the DMC meetings

4.1 Organisational meeting and early safety/trial integrity reviews

This charter was prepared early in the course of the trial (January 2015) by the trial sponsors, trial chief investigator, trial statistician, to be approved by the DMC at their first meeting. At the first meeting the DMC will provide an advisory review of scientific and ethical issues relating to study design and conduct, discuss the Charter for the role and functioning of the DMC, and the format

For peer review only - http://bmjopen.bmj.com/site/about/guidelines.xhtml

and content of the Open and Closed Reports that will be used to present trial results at future DMC meetings.

4.2 DMC meetings

The 1st meeting of the DMC has been scheduled before recruitment of the first participant, to discuss the protocol, the charter and the remit of the DMC, and to have the opportunity to clarify any aspects with the principal investigators, including the format of DMC reports from the Study Data Centre at the CHaRT Clinical Trials Unit.

Future meetings will be held at times to be determined by the DMC e.g. after a certain number of patients have been recruited, generally by teleconference, but face-to-face if necessary. The date of each meeting will be made available to the [unblinded/trial] statistician/analyst. The trial chief investigator and trial statistician/analyst will be available for an open session at the beginning of the meeting, and will be available at the end of the meeting to answer any urgent questions.

5. Procedures to ensure confidentiality & proper communication

To enhance the integrity and credibility of the trial, procedures will be implemented to ensure the DMC has sole access to emerging information from the clinical trial regarding safety data, aggregated by treatment arm. [An exception will be made to permit access to an unblinded statistician who will be responsible for creating the closed report and sending it to the DMC.] The sponsors will provide the chair of the DMC with information on any serious unexpected adverse device reactions to the study treatment, and will also be responsible for satisfying the standard requirements for reporting of relevant events to the appropriate authorities.

At the same time, procedures will be implemented to ensure proper communication is achieved between the DMC and the trial investigators and sponsor. To provide a forum for exchange of information among various parties that share responsibility for the successful conduct of the trial, a format for Open Sessions and Closed Sessions will be implemented. The intent of this format is to enable the DMC to preserve confidentiality of the unblinded results while at the same time providing opportunities for interaction between the DMC and others who have valuable insights into trial-related issues.

All materials, discussions and proceedings of the DMC are completely confidential.

5.1 Closed sessions

Sessions involving only DMC membership (but often including the unblinded statistician as well, as a non-voting member) called Closed Sessions will be held to allow discussion of confidential data from the clinical trial, including information about the safety of interventions. In order to ensure that the DMC will be fully informed in its primary mission of safeguarding the interest of participants, the DMC will be unblinded in its assessment of safety data. During these sessions, the DMC will

For peer review only - http://bmjopen.bmj.com/site/about/guidelines.xhtml

develop a consensus on its list of recommendations, including that relating to whether the trial should continue.

DMC members and all other participants in the closed section of DMC meetings and the production of unblinded reports are expected to maintain confidentiality, and will refrain from revealing to the TSC, or any other party, information that would lead to compromising the integrity of the trial unless such release is required to protect patient safety.

5.2 Open sessions

In order to allow the DMC to have adequate access to information provided by the trial investigators, or by members of the regulatory authorities, a joint session between these individuals and DMC members (called an Open Session) will be held before the Closed Session. If necessary, a further Open Session can be held, on request either in the middle or end of the Closed Session. Open sessions give the DMC an opportunity to query these individuals about issues that have arisen during their review in the initial Closed Session. With this format, important interactions are facilitated through which problems affecting trial integrity can be identified and resolved. These individuals will either be present at the DMC meeting or be provided a telephone link.

5.3 Open and Closed reports

For each DMC meeting, Open and Closed Reports will be provided (see Section 7 for outlines of the content of these reports). Open Reports, available to all who attend the DMC meeting, will include data on recruitment and pooled data on baseline characteristics, eligibility violations, completeness of follow-up and compliance. The trial manager and the trial statistician/analyst will supervise preparation of these Open Reports.

Closed Reports, available only to those attending the Closed Sessions of the DMC meeting, will include the same information plus adverse events by allocation. The trial statistician/analyst will produce the Closed Reports to the DMC.

The Open and Closed Reports should provide information that is as accurate as possible at the time of preparation, with follow-up that is as complete as possible to within approximately one month of the date of the DMC meeting. The Reports should be provided to DMC members at least one week prior to the date of the meeting.

5.4 Minutes of the DMC meeting

The [DMC Chair/other designated DMC member] will prepare minutes of the DMC meetings. Two sets will be prepared: The Open Minutes and the Closed Minutes.

The Open Minutes will describe the proceedings in the Open Session of the DMC meeting, and will summarize all recommendations by the DMC. Since these minutes will be circulated to the trial co-

For peer review only - http://bmjopen.bmj.com/site/about/guidelines.xhtml

chief investigators, it is essential that these minutes do not unblind any results, if the DMC is not recommending early termination.

The Closed Minutes will describe the proceedings from all sessions of the DMC meeting, including the listing of recommendations by the Committee. Because it is likely that these minutes will contain unblinded information, it is important that they are not made available to anyone outside the DMC. Rather, copies will be kept by [the DMC chair/other designated DMC member]. These will be sent to the trial manager and archived at the time of study closure.

5.5 Recommendations to the Trial Steering Committee

At each meeting of the DMC during the conduct of the trial, the DMC will make a recommendation to the TSC. Possible recommendations include:

- Trial continues as per protocol
- Early termination of the trial (or a recommendation for the TSC to review unblinded data), if significant safety issues arise
- Extending recruitment or extending follow-up

The recommendation(s) will be based primarily on safety considerations and will be guided by statistical monitoring guidelines defined in this Charter. Should the DMC decide to recommend early termination of the trial, a full vote of the independent DMC members will be required. All members of the DMC will be required for this to be quorate. In the event of a split vote, the decision will go with the majority vote, but a report should be provided to the TSC, detailing the split. This report should not include unblinded data unless deemed necessary by the DMC. This information should be forwarded to the trial chief investigator as rapidly as possible.

The TSC is jointly responsible with the DMC for safeguarding the interests of participants and for the conduct of the trial. Recommendations to amend the protocol or conduct of the study made by the DMC will be considered and either accepted or rejected by the TSC. The TSC will be responsible for deciding whether to continue or to stop the trial based on the DMC recommendations. The DMC will be notified of all changes to the protocol or to study conduct. The DMC concurrence will be sought on all substantive recommendations or changes to the protocol or study conduct prior to their implementation.

6. Statistical monitoring guidelines

During the period of recruitment into the study, information that is available on safety outcomes such as serious adverse device events believed to be related to treatment will be supplied, in strictest confidence, to all members of the DMC, along with any other analyses that the DMC may request.

For peer review only - http://bmjopen.bmj.com/site/about/guidelines.xhtml

7. Content of the DMC's open and closed reports

7.1 Open statistical report outline:

i. Report Information (milestones/gannt chart, date of data lock etc.)

ii. Protocol amendments and update on study documentation

iii. Site/central monitoring report

iv. Adherence to protocol (list of protocol deviations/violations)

v. Recruitment update/uptake rate

vi. Completeness of data collected (reporting missing data 'forms' overall and by site)

vii. Quality of data collected (reporting no. of queries/worst 'offenders' likewise)

viii. Safety reporting - list of Serious Adverse Events/SUSARs

ix. Withdrawals (i.e. adherence to intervention & retention)

   a. from intervention (plus reasons)

   b. from trial data collection i.e. losses to follow-up (plus reasons)

x. Other issues

xi. Recent relevant publications

7.2 Closed statistical report outline:

i. Report Information (date of data point/lock)

ii. Baseline comparability (for minimisation factors by group)

iii. Interim results for primary outcome(s) and selected secondary outcomes (either safety or process)

iv. Safety reporting – Unexpected Serious Adverse Device events/USADE (by group)

v. Safety reporting - list of Serious Adverse Events/SUSARs (to review event rates)

vi. Withdrawals (adherence to intervention & retention) (by group)

   i. from intervention (plus reasons)

   ii. trial data collection & follow-up (plus reasons)

For peer review only - http://bmjopen.bmj.com/site/about/guidelines.xhtml

8. DMC Payments

DMC members will be reimbursed for travel and accommodation. No other payments or rewards are anticipated but their contribution will be acknowledged where relevant in publications.

9. References

1. DAMOCLES Study Group. A proposed charter for clinical trial data monitoring committees: helping them to do their job well. The Lancet 2005; vol 365: 711-722.

For peer review only

For peer review only - http://bmjopen.bmj.com/site/about/guidelines.xhtml

STOPPIT 2 Publication guidelines

All relevant contributors will be acknowledged (by name where possible) in ensuing publications. Authorship will be assigned according to journal or ICMJE guidelines (see http://www.icmje.org/).

Briefly, ICMJE suggest that authorship credit should be based on: 1) substantial contributions to conception and design, acquisition of data, or analysis and interpretation of data; 2) drafting the article or revising it critically for important intellectual content; and 3) final approval of the version to be published. Authors should meet conditions 1, 2, and 3.

According to these guidelines therefore the sole activity of recruitment of participants or supervision of recruitment of participants, whilst crucially important, will qualify for contributorship, but is unlikely to qualify for authorship. The original grant applicants will qualify for authorship, as long as they continue to be involved in the study, and can also therefore satisfy conditions 2 and 3 above.

Final decisions on publication and authorship will be made by the trial steering committee.

For peer review only

Appendix 5 **PROTOCOL REVISIONS**

PROTOCOL VERSION	DATE	REASON FOR UPDATE	SUBSTANTIAL AMENDMENT NUMBER	SUMMARY OF CHANGES
2	02nd June 2015	Text reformatted and clarified, information updated<br>Questionnaires created and updated	1	**General updates/reformatting**<br>Clarification of secondary endpoints (obstetric/neonatal)<br>**Additional clinical information to be collected, to allow sensitivity analyses in future individual patient data meta-analyses**<br>Internal pilot phase interviews refined and made specific<br>Inclusion/exclusion criteria updated<br>Clarification of withdrawal<br>Pessary criteria updated<br>Ultrasound accreditation method updated<br>Qualitative interviews/questionnaires and Health Economics questionnaires included and information updated<br>PAG information updated<br>Study monitoring procedures updated<br>Trial Steering Committee details updated<br>Data Monitoring Committee details updated<br>Secondary end point definitions added (Appendix 5)
3	5th August 2015	Change of measurement which defines "short cervix" and confers eligibility for randomisation	2	Change of measurement which defines a short cervix from ≤ 30mm to ≤ 35mm.<br>The anticipated population centile (≤ 30th centile) is unchanged.
4	10th January 2018	Clarification of text, modification of the data collection and safety	5	• Clarification of Co-enrolment<br>• Modification of data collection to include hospitals, which are not participating STOPPIT2 sites<br>• Safety reporting guidance modified for clarity.<br>• Increase in the number of participants from 1850 to 2500 for cervical length scanning to facilitate randomisation which remains at 500.

SPIRIT

STANDARD PROTOCOL ITEMS: RECOMMENDATIONS FOR INTERVENTIONAL TRIALS

SPIRIT 2013 Checklist: Recommended items to address in a clinical trial protocol and related documents*

Section/item	Item No	Description
**Administrative information**		
Title	1	Descriptive title identifying the study design, population, interventions, and, if applicable, trial acronym Page 1
Trial registration	2a	Trial identifier and registry name. If not yet registered, name of intended registry Page 4
	2b	All items from the World Health Organization Trial Registration Data Set Page 4
Protocol version	3	Date and version identifier Page 12
Funding	4	Sources and types of financial, material, and other support Page 13
Roles and responsibilities	5a	Names, affiliations, and roles of protocol contributors Page 1
	5b	Name and contact information for the trial sponsor Page 13
	5c	Role of study sponsor and funders, if any, in study design; collection, management, analysis, and interpretation of data; writing of the report; and the decision to submit the report for publication, including whether they will have ultimate authority over any of these activities Page 13
	5d	Composition, roles, and responsibilities of the coordinating centre, steering committee, endpoint adjudication committee, data management team, and other individuals or groups overseeing the trial, if applicable (see Item 21a for data monitoring committee) Appendix for DMC
**Introduction**		
Background and rationale	6a	Description of research question and justification for undertaking the trial, including summary of relevant studies (published and unpublished) examining benefits and harms for each intervention Page 3-4
	6b	Explanation for choice of comparators Page 3-4
Objectives	7	Specific objectives or hypotheses Page 6

For peer review only

Trial design	8	Description of trial design including type of trial (eg, parallel group, crossover, factorial, single group), allocation ratio, and framework (eg, superiority, equivalence, noninferiority, exploratory) Page 4

Methods: Participants, interventions, and outcomes

Study setting	9	Description of study settings (eg, community clinic, academic hospital) and list of countries where data will be collected. Reference to where list of study sites can be obtained Page 6
Eligibility criteria	10	Inclusion and exclusion criteria for participants. If applicable, eligibility criteria for study centres and individuals who will perform the interventions (eg, surgeons, psychotherapists) Page 5
Interventions	11a	Interventions for each group with sufficient detail to allow replication, including how and when they will be administered Page 6
	11b	Criteria for discontinuing or modifying allocated interventions for a given trial participant (eg, drug dose change in response to harms, participant request, or improving/worsening disease) Page 7
	11c	Strategies to improve adherence to intervention protocols, and any procedures for monitoring adherence (eg, drug tablet return, laboratory tests) Page 8
	11d	Relevant concomitant care and interventions that are permitted or prohibited during the trial Page 6
Outcomes	12	Primary, secondary, and other outcomes, including the specific measurement variable (eg, systolic blood pressure), analysis metric (eg, change from baseline, final value, time to event), method of aggregation (eg, median, proportion), and time point for each outcome. Explanation of the clinical relevance of chosen efficacy and harm outcomes is strongly recommended Page 6
Participant timeline	13	Time schedule of enrolment, interventions (including any run-ins and washouts), assessments, and visits for participants. A schematic diagram is highly recommended (see Figure) Page 8
Sample size	14	Estimated number of participants needed to achieve study objectives and how it was determined, including clinical and statistical assumptions supporting any sample size calculations Page 11
Recruitment	15	Strategies for achieving adequate participant enrolment to reach target sample size Page 6

Methods: Assignment of interventions (for controlled trials)

Allocation:

Sequence generation	16a	Method of generating the allocation sequence (eg, computer-generated random numbers), and list of any factors for stratification. To reduce predictability of a random sequence, details of any planned restriction (eg, blocking) should be provided in a separate document that is unavailable to those who enrol participants or assign interventions Page 4
Allocation concealment mechanism	16b	Mechanism of implementing the allocation sequence (eg, central telephone; sequentially numbered, opaque, sealed envelopes), describing any steps to conceal the sequence until interventions are assigned Page 5
Implementation	16c	Who will generate the allocation sequence, who will enrol participants, and who will assign participants to interventions Page 4, 5, 6
Blinding (masking)	17a	Who will be blinded after assignment to interventions (eg, trial participants, care providers, outcome assessors, data analysts), and how Page 5
	17b	If blinded, circumstances under which unblinding is permissible, and procedure for revealing a participant's allocated intervention during the trial n/a

Methods: Data collection, management, and analysis

Data collection methods	18a	Plans for assessment and collection of outcome, baseline, and other trial data, including any related processes to promote data quality (eg, duplicate measurements, training of assessors) and a description of study instruments (eg, questionnaires, laboratory tests) along with their reliability and validity, if known. Reference to where data collection forms can be found, if not in the protocol Page 6
	18b	Plans to promote participant retention and complete follow-up, including list of any outcome data to be collected for participants who discontinue or deviate from intervention protocols Page 8
Data management	19	Plans for data entry, coding, security, and storage, including any related processes to promote data quality (eg, double data entry; range checks for data values). Reference to where details of data management procedures can be found, if not in the protocol Page 9
Statistical methods	20a	Statistical methods for analysing primary and secondary outcomes. Reference to where other details of the statistical analysis plan can be found, if not in the protocol Page 9
	20b	Methods for any additional analyses (eg, subgroup and adjusted analyses) Page 10
	20c	Definition of analysis population relating to protocol non-adherence (eg, as randomised analysis), and any statistical methods to handle missing data (eg, multiple imputation) Page 9

Methods: Monitoring

Data monitoring	21a	Composition of data monitoring committee (DMC); summary of its role and reporting structure; statement of whether it is independent from the sponsor and competing interests; and reference to where further details about its charter can be found, if not in the protocol. Alternatively, an explanation of why a DMC is not needed Page 10
	21b	Description of any interim analyses and stopping guidelines, including who will have access to these interim results and make the final decision to terminate the trial Page 9, 10
Harms	22	Plans for collecting, assessing, reporting, and managing solicited and spontaneously reported adverse events and other unintended effects of trial interventions or trial conduct Page 6
Auditing	23	Frequency and procedures for auditing trial conduct, if any, and whether the process will be independent from investigators and the sponsor Page 10

Ethics and dissemination

Research ethics approval	24	Plans for seeking research ethics committee/institutional review board (REC/IRB) approval Page 12
Protocol amendments	25	Plans for communicating important protocol modifications (eg, changes to eligibility criteria, outcomes, analyses) to relevant parties (eg, investigators, REC/IRBs, trial participants, trial registries, journals, regulators) Page 12
Consent or assent	26a	Who will obtain informed consent or assent from potential trial participants or authorised surrogates, and how (see Item 32) Page 9
	26b	Additional consent provisions for collection and use of participant data and biological specimens in ancillary studies, if applicable n/a
Confidentiality	27	How personal information about potential and enrolled participants will be collected, shared, and maintained in order to protect confidentiality before, during, and after the trial Page 10
Declaration of interests	28	Financial and other competing interests for principal investigators for the overall trial and each study site Page 14
Access to data	29	Statement of who will have access to the final trial dataset, and disclosure of contractual agreements that limit such access for investigators Page 11
Ancillary and post-trial care	30	Provisions, if any, for ancillary and post-trial care, and for compensation to those who suffer harm from trial participation Page 8

For peer review only

Dissemination policy	31a	Plans for investigators and sponsor to communicate trial results to participants, healthcare professionals, the public, and other relevant groups (eg, via publication, reporting in results databases, or other data sharing arrangements), including any publication restrictions Page 12
	31b	Authorship eligibility guidelines and any intended use of professional writers Page 12
	31c	Plans, if any, for granting public access to the full protocol, participant-level dataset, and statistical code Page 12
**Appendices**		
Informed consent materials	32	Model consent form and other related documentation given to participants and authorised surrogates Appendix tbc
Biological specimens	33	Plans for collection, laboratory evaluation, and storage of biological specimens for genetic or molecular analysis in the current trial and for future use in ancillary studies, if applicable n/a

*It is strongly recommended that this checklist be read in conjunction with the SPIRIT 2013 Explanation & Elaboration for important clarification on the items. Amendments to the protocol should be tracked and dated. The SPIRIT checklist is copyrighted by the SPIRIT Group under the Creative Commons "Attribution-NonCommercial-NoDerivs 3.0 Unported" license.

For peer review only
