# Supplementary material for: Open randomised trial of the (Arabin) pessary to prevent preterm birth in twin pregnancy with health economics and acceptability: STOPPIT-2—a study protocol
Source: BMJ Open. 2018 Dec 6;8(12):e026430. doi: 10.1136/bmjopen-2018-026430 (PMC6286620; doi:10.1136/bmjopen-2018-026430)
Supplement: Reviewer comments [file bmjopen-2018-026430.reviewer_comments.pdf]

PEER REVIEW HISTORY

BMJ Open publishes all reviews undertaken for accepted manuscripts. Reviewers are asked to complete a checklist review form (**http://bmjopen.bmj.com/site/about/resources/checklist.pdf**) and are provided with free text boxes to elaborate on their assessment. These free text comments are reproduced below.

ARTICLE DETAILS

**TITLE (PROVISIONAL)**	Protocol for an open randomised trial of the (Arabin) pessary to prevent preterm birth in twin pregnancy, with health economics and acceptability – STOPPIT-2.
**AUTHORS**	Norman, Jane; Norrie, John; Maclennan, Graeme; Cooper, David; Whyte, Sonia; CunninghamBurley, Sarah; Smith, Joel; Shennan, Andrew; Robson, Stephen; Thornton, Steven; Kilby, Mark; Marlow, Neil; Stock, Sarah Jane; Bennett, Philip; Denton, Jane

This article was not externally reviewed at BMJ Open. Protocols that have been independently assessed prior to submission to BMJ Open are usually fast-tracked to publication on the grounds that further substantial changes will not be possible. This independent assessment will usually be external, independent review for both a substantial grant award from a non-commercial or government funder and ethics approval.
